# Comparison of methods for tuning machine learning model hyper-parameters: with application to predicting high-need high-cost health care users

**DOI:** 10.1186/s12874-025-02561-x

**Published:** 2025-05-15

**Authors:** Christopher Meaney, Xuesong Wang, Jun Guan, Therese A. Stukel

**Affiliations:** 1 Department of Family and Community Medicine, University of Toronto, Toronto, Canada; 2 ICES, Toronto, Canada; 3 Institute for Health, Policy, Management and Evaluation, University of Toronto, Toronto, Canada

**Keywords:** Supervised machine learning, Clinical predictive modelling, Prediction model, Hyper-parameter optimization (HPO), Hyper-parameter tuning (HPT), Extreme gradient boosting classifier

## Abstract

**Background:**

Supervised machine learning is increasingly being used to estimate clinical predictive models. Several supervised machine learning models involve hyper-parameters, whose values must be judiciously specified to ensure adequate predictive performance.

**Objective:**

To compare several (nine) hyper-parameter optimization (HPO) methods, for tuning the hyper-parameters of an extreme gradient boosting model, with application to predicting high-need high-cost health care users.

**Methods:**

Extreme gradient boosting models were estimated using a randomly sampled training dataset. Models were separately trained using nine different HPO methods: 1) random sampling, 2) simulated annealing, 3) quasi-Monte Carlo sampling, 4-5) two variations of Bayesian hyper-parameter optimization via tree-Parzen estimation, 6-7) two implementations of Bayesian hyper-parameter optimization via Gaussian processes, 8) Bayesian hyper-parameter optimization via random forests, and 9) the covariance matrix adaptation evolutionary strategy. For each HPO method, we estimated 100 extreme gradient boosting models at different hyper-parameter configurations; and evaluated model performance using an AUC metric on a randomly sampled validation dataset. Using the best model identified by each HPO method, we evaluated generalization performance in terms of discrimination and calibration metrics on a randomly sampled held-out test dataset (internal validation) and a temporally independent dataset (external validation).

**Results:**

The extreme gradient boosting model estimated using default hyper-parameter settings had reasonable discrimination (AUC=0.82) but was not well calibrated. Hyper-parameter tuning using any HPO algorithm/sampler improved model discrimination (AUC=0.84), resulted in models with near perfect calibration, and consistently identified features predictive of high-need high-cost health care users.

**Conclusions:**

In our study, all HPO algorithms resulted in similar gains in model performance relative to baseline models. This finding likely relates to our study dataset having a large sample size, a relatively small number of features, and a strong signal to noise ratio; and would likely apply to other datasets with similar characteristics.

**Supplementary Information:**

The online version contains supplementary material available at 10.1186/s12874-025-02561-x.

## Background

Hyper-parameter optimization (HPO) is a sub-field of machine learning focused on identifying a tuple of model-specific hyper-parameters that maximize model performance. Our study comparatively evaluates how different HPO methods can be used to tune the hyper-parameters of a supervised extreme gradient boosting model, aiming to predict high-need high-cost health care users.

Hyper-parameter optimization has been well studied by the computer science and machine learning research communities. Hutter et al. [17] comprehensively covers HPO methods. Bischl et al. [9] review several HPO methods, including: methods based on (pseudo-) random search and other stochastic sampling processes, Bayesian optimization methods relying on surrogate models, and evolutionary strategies based on biological concepts (e.g. mutation, cross-over, selection, etc.). Yu et al. [27] review HPO methods and their applications in machine learning. Bergstra et al. [6, 7] compare the performance of several hyperopt samplers on the HPOlib benchmarks [8]. Putatunda et al. [21] compare hyperopt samplers for tuning extreme gradient boosting prediction models across several datasets. Kegl et al. [18] review and compare modern HPO methods for tuning predictive models developed on tabular datasets. Shekhar et al. [22] survey and compare the performance of several modern HPO methods for learning predictive models for various datasets. The past literature suggests that research problems/objectives (e.g. classification, regression, unsupervised learning, etc.) and study dataset characteristics (e.g. sample size, number of features, signal-to-noise ratio, etc.) seem to influence the relative performance of HPO methods investigated. Our study adds to this body of literature, comparatively evaluating the performance of several HPO methods, when used to estimate an extreme gradient boosting model for predicting high-need high-cost health care users (using a dataset characterized by a large sample size, a small number of features, and a strong signal-to-noise ratio).

In the biomedical research community, the updated TRIPOD-AI reporting guidelines [12] have explicitly stated researchers developing clinical predictive models should transparently report on all methods used to tune model hyper-parameters. We note that several previous reporting guidelines for clinical predictive models did not explicitly mention hyper-parameter optimization/tuning [11]. Research on the completeness of reporting of clinical prediction modelling studies similarly found little evidence that HPO methods were being used [1]. We identified a single article using a Monte Carlo simulation study to investigate impacts of HPO methods on clinical predictive models [13]; that said, the study focused on scenarios involving small sample sizes and a small number of features. Hence, study findings may not generalize to clinical prediction models estimated on larger datasets (e.g. health administrative datasets, electronic health record datasets, datasets from large patient reported surveys). Using a series of PubMed searches we identified ~ 45k peer-reviewed publications, in the past 5 years, focusing on supervised machine learning for estimating clinical prediction models; however, < 200 of these articles explicitly mentioned hyper-parameter optimization/tuning methods in their manuscript title, abstract, or PubMed index terms (Supplementary Material 1). Our study aims to address a knowledge gap resulting from a paucity of research on HPO methods in the context of clinical predictive modelling. Our study synthesizes important literature focusing on hyper-parameter optimization/tuning; we showcase several modern HPO software/methods; and we compare the performance of HPO methods on a single benchmark dataset, used to predict high-need high-cost health care users (which we think is exemplary of many large tabular datasets used in clinical predictive modelling).

We structure the remainder of our manuscript as follows. The “Methods” section begins by reviewing the extreme gradient boosting model and model-specific hyper-parameters. Next, we review HPO methods used in this study: 1) methods based on probabilistic processes (e.g. random search, simulated annealing, and quasi Monte Carlo sampling), 2) Bayesian optimization models using surrogate models (e.g. the tree-Parzen estimator, Gaussian process models, and Bayesian optimization with random forests), and 3) models based on evolutionary strategies. Following, we discuss our high-need high-cost dataset, outcome/feature variables, and sample characteristics. Finally, we discuss our HPO study design and metrics used to comparatively evaluate the performance of HPO methods, used to estimate extreme gradient boosting models, for predicting high-need high-cost health care users. The “Results” section compares HPO methods in terms of discrimination/calibration metrics, feature importance rankings, and the computational time required to run the HPO experiments. The “Discussion” section summarizes and contextualizes the findings of our research study. We end by highlighting some strengths/limitations of our research study and suggesting areas for future investigation.

## Methods

### Extreme gradient boosting model

Our study focused on tuning the hyper-parameters of an extreme gradient boosting model for predicting a binary outcome, denoting high-need high-cost health care status. Details regarding the theory and computation of gradient boosting are discussed in Friedman [14] and Chen et al. [10]. The extreme gradient boosting model has performed well in many empirical predictive tasks; however, it is associated with several hyper-parameters that must be judiciously tuned for the resulting supervised machine learning model to have optimal performance.

We used the Python XGBoost implementation of the extreme gradient boosting classifier (https://xgboost.readthedocs.io/en/stable/python/python_api.html). Extreme gradient boosting model hyper-parameters and their default values are presented in Table 1. The tuning range (support) for extreme gradient boosting hyper-parameters are also described in Table 1. Choices regarding tuning range are governed by the measurement scale and support of the specific hyper-parameter under consideration.

**Table 1:** Extreme gradient boosting model hyper-parameters, default values, and tuning ranges for hyper-parameter optimization experiments

Model	Hyper-parameter	Abbreviation	Default Value	Tuning Range/Support
Extreme Gradient Boosting Model	Number of Boosting Rounds	“trees”	10	DiscreteUniform(100…1000)
	Learning Rate	“lr”	0.3	ContinuousUniform(0,1)
	Maximum Tree Depth	“depth”	6	DiscreteUniform(1…25)
	Minimum Leaf Weight	“cw”	1	DiscreteUniform(1…10)
	Gamma Regularization	“gamma”	None	ContinuousUniform(0,5)
	Alpha Regularization	“alpha”	0	ContinuousUniform(0,1)
	Lambda Regularization	“lambda”	1	ContinuousUniform(0,1)
	Row Sample Fraction	“rowsample”	1	ContinuousUniform(0,1)
	Column Sample Fraction	“colsample”	1	ContinuousUniform(0,1)

### Hyper-parameter optimization

Hyper-parameter optimization algorithms seek to identify an optimal set of hyper-parameters (λ) that optimize an objective function - $f(\lambda )$ - corresponding to a user-selected evaluation metric [9, 17]. The choice of evaluation metric is problem dependent. Metric choice will govern whether HPO is viewed as a minimization/maximization problem. In our study we focus on an AUC metric suitable for evaluating the performance of binary prediction models. We note that other metric functions exist for evaluating binary prediction models (e.g. binomial/cross-entropy loss, F1 score, etc.). Formally, hyper-parameter tuning methods attempt to optimize the objective below:

$${\lambda }^{*}= \text{arg}\underset{\lambda \in\Lambda }{\text{max}}\ f(\lambda )$$

- $\lambda$ is a particular hyper-parameter configuration. We note that $\lambda$ is a J-dimensional tuple $\lambda \equiv ({\lambda }_{1},{\lambda }_{2},...,{\lambda }_{J})$. We use ${\lambda }_{j}$ to denote the j^th^ hyper-parameter in the tuple.
- $\Lambda$ defines the search space (support) of the hyperparameters. In our study, $\Lambda$ is a product space over (bounded) continuous and discrete variables.
- $f(\lambda )$ denotes the metric function employed in the HPO experiment, which evaluates model performance at a given hyper-parameter configuration ($\lambda \in\Lambda$). In our study, we use an AUC metric for evaluating the performance of our binary prediction model – and HPO is cast as an AUC maximization problem.
- ${\lambda }^{*}$ is the optimal hyper-parameter configuration that yields the best model performance (with respect to the user defined metric function).
- We assume s= 1…S trials in our HPO experiment. ${\lambda }^{s}$ denotes the hyper-parameter configuration used in the s^th^ trial of the HPO experiment. In this study, we budget S= 100 trials for each HPO method being comparatively evaluated.

### Hyper-parameter optimization algorithms

#### Hyperopt: random sampling

Candidate hyper-parameter configurations are randomly and independently sampled from univariate probability distributions (e.g. bounded discrete/continuous distributions) [5].

$${\lambda }_{j} \sim {P}_{j}({\Lambda }_{j})$$

${\lambda }_{j}$ denotes a realization of a specific hyper-parameter (j^th^ element of the tuple). ${P}_{j}({\Lambda }_{j})$ is the probability distribution from which ${\lambda }_{j}$ is randomly drawn/sampled.

#### Hyperopt: simulated annealing

Simulated annealing treats hyper-parameter search as an energy minimization problem, where the metric function ($f(\lambda )$) represents energy, and solutions are perturbed stochastically until an optimum is identified. The algorithm accepts worse solutions with the following probability [19]:

$$P\left(\Delta f, T\right)= \text{exp}\left(-\frac{\Delta f}{ T}\right)$$

$\Delta f=f\left({\lambda }^{s+1}\right)- f\left({\lambda }^{s}\right)$, is the change in the objective function value between successive HPO trials. T is the annealing temperature (a meta-parameter of the HPO algorithm). Initially, simulated annealing explores the hyper-parameter space broadly by accepting poor solutions with high probability, but as T cools, the acceptance rate of worse solutions decreases, allowing the method to converge.

#### Hyperopt: tree-structured Parzen estimator

The tree-Parzen estimator is a Bayesian optimization algorithm that models the conditional probability distribution $P({\lambda }^{s+1} | ({f}({\lambda }^{s}),\ { f}({\lambda }^{s-1}),\dots ,\ {f}({\lambda }^{1}))$ instead of modeling $f(\lambda )$ directly. The tree-Parzen estimator approach divides observed hyper-parameter configurations into two sets:

- ${g}$: configurations where the metric value exceeds a threshold: i.e. $f\left(\lambda \right)>{f}^{*}$.
- $\mathcal{L}$: configurations where the metric value is less than a threshold: i.e. $f\left(\lambda \right) \le {f}^{*}$.

The tree-Parzen estimator fits two nonparametric kernel density estimators to approximate P(λ |${g}$) and P(λ |$\mathcal{L}$), respectively. The algorithm chooses new hyper-parameter configurations to maximize expected improvement, which can be shown proportional to maximizing the expression below: [4]:

$${\lambda }^{*}=\text{arg}\underset{\lambda \in\Lambda }{\text{max}}\frac{P\left(\lambda \right|{g})}{P(\uplambda |\mathcal{L})}$$

#### Hyperopt: adaptive tree-structured Parzen estimator

The adaptive tree-Parzen estimator extends the standard tree-Parzen estimator by dynamically adjusting the trade-off between exploration and exploitation based on optimization progress. Early in the search, it favors exploration to gain a broad understanding of the search space, while later it prioritizes exploitation by focusing more on high-performing regions. This is achieved by dynamically varying the threshold ${(f}^{*})$ throughout the course of the hyper-parameter optimization experiment [4].

#### Optuna: Gaussian Process

Gaussian Process (GP) based hyper-parameter optimization attempts to fit a surrogate model for the HPO metric function ($f(\lambda )$) using a distribution over functions [23]. The Gaussian Process used in HPO is defined as:

$$GP(\mu \left(\lambda \right), k(\lambda ,{\lambda }^{\prime}))$$

Where $\mu \left(\lambda \right)$ is the GP mean function, and $, k(\lambda ,{\lambda }^{\prime})$ is the GP covariance/kernel function. The GP mean and covariance functions are updated using past hyper-parameter configurations. Given past evaluations of the metric function, GP-based optimization uses an acquisition function (e.g., expected improvement, upper confidence bound, probability of improvement, etc.) to determine which hyper-parameter configuration to sample next.

#### Optuna: Quasi-Monte Carlo sampler

Quasi Monte Carlo methods improve on random search by using low-discrepancy sequences instead of purely random samples (e.g. Sobol sequences, and related processes). These sequences fill the search space more uniformly than independent random draws, reducing variance in HPO metric function evaluations [5].

#### Optuna: CMA-ES (Covariance Matrix Adaptation Evolution Strategy)

CMA-ES is an evolutionary algorithm for hyper-parameter optimization that iteratively updates the parameters of a multivariate normal (MVN) distribution, $MVN(\mu (\lambda ),\Sigma (\lambda ))$. The mean $\mu (\lambda )$ represents the center of promising hyper-parameter configurations, while the covariance matrix $\Sigma (\lambda )$ captures dependencies between hyper-parameters, allowing the algorithm to model correlations and adapt the search distribution over successive trials of the HPO experiment.

CMA-ES follows an evolutionary process: in each iteration, the CMA-ES algorithm selects the best-performing hyper-parameter configurations (analogous to survival/fitness) and updates the MVN mean and covariance (similar to recombination and mutation). These adaptive updates refine the HPO search space, enabling CMA-ES to efficiently explore and sample better-performing hyper-parameter sets [15, 16].

#### Scikit-Optimize (skopt): Gaussian Process

SkOpt uses a Gaussian process (GP) surrogate model for hyper-parameter optimization. SkOpt supports flexible kernel selection and acquisition function tuning, adapting to different search spaces. SkOpt efficiently balances exploration and exploitation in hyper-parameter search.

#### SMAC3: Bayesian optimization with random forests

SMAC3 is a Bayesian optimization framework for hyper-parameter tuning that uses random forests to estimate a surrogate model of the HPO metric function [20]. SMAC3 builds an ensemble of regression trees to estimate performance, making it robust in high-dimensional and discrete spaces. The expected improvement acquisition function balances exploration and exploitation to efficiently select the next hyper-parameter configuration.

### Description of the high-need high-cost dataset: outcome and feature variables

Total individual health care costs were calculated for all Ontario adult residents > 18 years old separately during fiscal year 2017 and fiscal year 2019 using provincial health administrative data. Individual health conditions were captured over the previous two fiscal years. Total costs were dichotomized into a binary outcome, denoting whether an individual was in the top-5 percentile of the empirical cost distribution, separately for fiscal year 2017 and fiscal year 2019.

Our study is restricted to older adults aged > 65 years (*n*= 2,321,168). Median age was 73 years old (IQR: 68–80 years) and 54.6% of the sample was female. A total of 345,503 (15.3%) of older Ontario residents experienced costs in the top-5 percentile during fiscal year 2017.

The 2019 population contained 2,483,064 residents aged > 65 years. Of these, 366,219 (14.7%) were in the top-5 percentile of the empirical cost distribution during fiscal year 2019. Demographic and clinical characteristics of health care users were similar in 2017 and 2019.

Subject matter experts from medicine, epidemiology, and biostatistics purposefully selected features for inclusion in our prediction models that they hypothesized to be drivers of high-need high-cost health care utilization. The dataset consists of approximately 104 features, resulting in a clinical tabular dataset whose design matrix contained 143 columns (Supplementary Material 2). The features measure patient socio-demographic characteristics, diagnoses with physical/mental health conditions, past hospitalizations and other previous healthcare utilization. Most of the variables are binary valued, denoting the presence/absence of a health condition or health care utilization. Few variables are categorical. Two continuous measures exist in the dataset (i.e. age in years, and frailty index score). All variables were hypothesized to have a connection with high-need high-cost health care utilization, and as such the dataset is characterized by a strong signal to noise ratio. The ratio of samples-to-features is large, as is the ratio of samples-to-events (i.e. high-need high-cost health care users).

### Study design and evaluation metrics for hyper-parameter optimization experiments

For each of the nine HPO methods being compared, we budget S= 100 trials for each HPO experiment. A single trial (s= 1…S) in our experiment involves: 1) identification of a tuple of hyper-parameters, 2) optimization of the extreme gradient boosting model at the particular hyper-parameter configuration on the training dataset (n.b. the same training dataset is used for estimating the extreme gradient boosting model under each sampled hyper-parameter configuration), and 3) estimation of an AUC evaluation metric on the held-out validation dataset (n.b. the same validation dataset is used for AUC estimation and selection of an optimal tuple of hyper-parameters which maximizes model performance). Given nine HPO methods, we conduct nine HPO experiments, each with a budget of 100 trials. We train 900 extreme gradient boosting models and estimate 900 replicates of the AUC evaluation metric. We note that the budget of S= 100 trials (for each HPO method) is an arbitrary choice. Choice of HPO budget (S) is often informed by the size of the training dataset, the statistical model being fitted, and availability of high-performance computing facilities – as these factors impact model fitting time, and total runtime of the entire HPO experiment.

For each of the HPO methods/experiments we construct a line plot illustrating variation in the metric function over sequential trials, at distinct hyper-parameter configurations. The plot allows for graphical identification of the optimal extreme gradient boosting model identified using each HPO method, comparison of the optimal model against learned alternative models, and comparison against the extreme gradient boosting model fitted using default hyper-parameter values.

We use a random 80:10:10 training/validation/test split of the fiscal year 2017 dataset. Predictions from optimal models identified by each of the hyper-parameter optimization methods are estimated using a randomly selected hold-out test dataset, and generalization performance is evaluated (internal validation). A temporally independent fiscal year 2019 test dataset is also used to evaluate model generalization performance (external validation). The same training, validation, and test datasets are used to evaluate each of the HPO methods being compared, to ensure comparability of discrimination/calibration metrics and inferences regarding feature importance.

The study considers several metrics of discrimination and calibration performance [24–26]. We evaluate model discrimination performance using accuracy, sensitivity (a.k.a. recall), specificity, positive predictive value (PPV; a.k.a. precision), negative predictive value (NPV), and area under the receiver operating characteristic curve (AUC). For each machine learning model and hyper-parameter optimization method, we plot a receiver operating characteristic (ROC) curve and LOESS smoothed calibration curve [2]. We also evaluate model calibration performance using the integrated calibration index (ICI) and the E50, E90 and Emax indices [3]. ICI measures the (integrated) area between the observed and ideal calibration curves; whereas, E50, E90, and Emax measure the median, 90^th^ percentile, and maximum deviation between observed and ideal calibration curves. We use a total gain feature importance metric to compare the stability of important features identified by each of the extreme gradient boosting models, and associated HPO methods. HPO algorithms are also compared in terms of runtime over the 100 budgeted experiments (n.b. runtime is measured in hours).

## Results

### Model training, hyperparameter optimization and model selection

Using the default hyper-parameter values, validation AUC was 0.82 for the extreme gradient boosting model fit. Each of the nine hyper-parameter optimization methods resulted in similar improvements in validation AUC (0.84) (Table 2).

**Table 2:** Validation dataset AUC metrics, hyper-parameter optimization time, and optimally identified hyper-parameter configurations for each hyper-parameter optimization method

Hyper-Parameter Optimization Method	Optimal AUC Estimated on the 2017 Validation Dataset	Hyper-Parameter Optimization Time (Hours)	Optimal Hyper-Parameter Configuration Identified by each HPO Method
Default Hyper-parameters	0.823	NA^a^	trees= 10; lr= 0.3; depth= 6; cw= 1; gamma=None; lambda= 1; alpha= 0; colsample= 1; subsample= 1
Hyperopt - Random Search	0.842	7.6	trees= 887; lr= 0.099; depth= 7; cw= 5; gamma= 4.511; lambda= 0.921; alpha= 0.687; colsample= 0.348; subsample= 0.897
Hyperopt - Simulated Annealing	0.842	5.2	trees= 340; lr= 0.116; depth= 10; cw= 3; gamma= 3.486; lambda= 0.608; alpha= 0.48; colsample= 0.239; subsample= 0.908
Hyperopt - Tree Parzen Estimator	0.842	11.7	trees= 717; lr= 0.11; depth= 15; cw= 6; gamma= 3.537; lambda= 0.062; alpha= 0.884; colsample= 0.204; subsample= 0.925
Hyperopt - Adaptive Tree Parzen Estimator	0.842	10.6	trees= 855; lr= 0.058; depth= 10; cw= 5; gamma= 4.052; lambda= 0.161; alpha= 0.221; colsample= 0.448; subsample= 0.922
Optuna - Quasi Monte Carlo	0.841	9.4	trees= 817; lr= 0.016; depth= 24; cw= 9; gamma= 3.984; lambda= 0.234; alpha= 0.422; colsample= 0.547; subsample= 0.172
Optuna - Gaussian Process	0.841	8.7	trees= 623; lr= 0.279; depth= 8; cw= 9; gamma= 3.684; lambda= 0.82; alpha= 1; colsample= 0.206; subsample= 0.508
Optuna - Covariance Matrix Adaption Evolutionary Strategy	0.842	7.6	trees= 381; lr= 0.076; depth= 17; cw= 6; gamma= 3.736; lambda= 0.059; alpha= 0.614; colsample= 0.219; subsample= 0.75
SkOpt - Gaussian Process	0.842	9.1	trees= 918; lr= 0.051; depth= 11; cw= 10; gamma= 2.176; lambda= 0.138; alpha= 0.234; colsample= 0.298; subsample= 1
SMAC3 - Bayesian Optimization	0.841	6.4	trees= 196; lr= 0.154; depth= 9; cw= 8; gamma= 1.001; lambda= 0.947; alpha= 0.993; colsample= 0.274; subsample= 0.591

^a^NA indicates that HPO time is “not applicable” because the XGBoost model was fit using default hyper-parameter values

Time to run the hyper-parameter optimization experiments is given in Table 2. The hyperopt simulated annealing method resulted in the shortest runtime of all the HPO methods/experiments (5.2 hours).

**Fig. 1:**
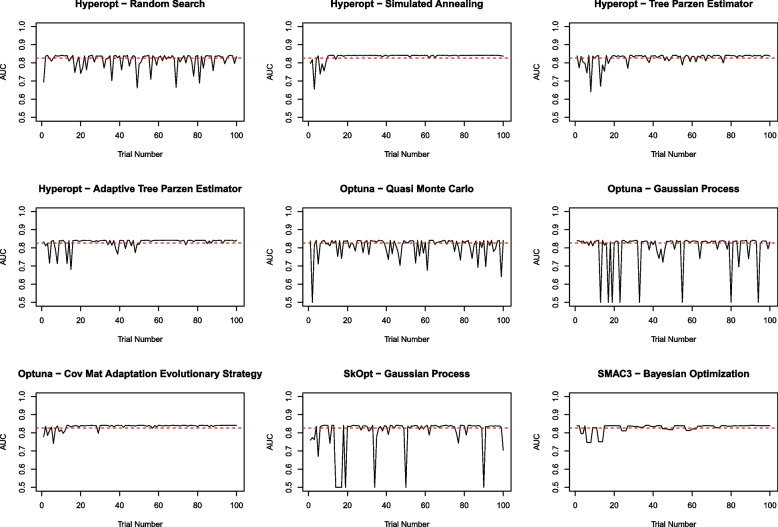
AUC metric estimated on the randomly sampled fiscal year 2017 validation dataset for each hyper-parameter optimization method, over S= 100 budgeted trials from the HPO experiment. The dashed red line represents AUC estimated from fitting the binary extreme gradient boosting model using default hyper-parameter settings

Tuples of optimal hyper-parameter values identified by each HPO method appear qualitatively different (Table 2). This suggests that the metric function may contain multiple local optima, whereby distinct hyper-parameter configurations result in similar AUC values. This finding is corroborated by Fig. 1 which plots AUC statistics over the S= 100 trials in each HPO experiment. Increasing the value of the “trees” hyper-parameter, controlling the number of base learners (i.e. decision trees) in the XGBoost ensemble, allows HPO methods to realize gains in predictive model performance (relative to baseline models fit using the default hyper-parameter values).

### Model discrimination and calibration performance on the 2017 test dataset

Table 3 summarizes model discrimination and calibration performance estimated on the fiscal year 2017 test dataset (internal validation). Using default hyper-parameter values AUC= 0.82. AUC improved when employing all nine hyper-parameter optimization methods (AUC= 0.84). At the default hyper-parameter configuration, specificity was slightly higher and sensitivity was lower than what was observed from all the HPO methods.

**Table 3:** Discrimination and calibration performance estimated on the 2017 test dataset, for each hyper-parameter optimization method

Hyper-Parameter Optimization Method	Acc	Sens	Spec	PPV	NPV	AUC	ICI	E50	E90	Emax
Default Hyper-parameters	0.888	0.354	0.984	0.803	0.894	0.823	0.026	0.028	0.036	0.063
Hyperopt - Random Search	0.891	0.415	0.977	0.762	0.903	0.842	0.002	0.002	0.004	0.008
Hyperopt - Simulated Annealing	0.891	0.414	0.977	0.761	0.903	0.842	0.002	0.002	0.004	0.007
Hyperopt - Tree Parzen Estimator	0.890	0.414	0.976	0.759	0.902	0.842	0.002	0.001	0.003	0.010
Hyperopt - Adaptive Tree Parzen Estimator	0.891	0.414	0.977	0.761	0.902	0.842	0.002	0.002	0.004	0.009
Optuna - Quasi Monte Carlo	0.891	0.414	0.977	0.763	0.903	0.842	0.001	0.000	0.002	0.006
Optuna - Gaussian Process	0.890	0.412	0.976	0.758	0.902	0.841	0.003	0.002	0.005	0.015
Optuna - Covariance Matrix Adaption Evolutionary Strategy	0.891	0.412	0.977	0.763	0.902	0.842	0.001	0.001	0.003	0.005
SkOpt - Gaussian Process	0.891	0.414	0.977	0.761	0.902	0.842	0.002	0.002	0.003	0.008
SMAC3 - Bayesian Optimization	0.891	0.413	0.977	0.760	0.902	0.841	0.002	0.002	0.005	0.007

*Acc*=Accuracy; *Sens*=Sensitivity; *Spec*=Specificity; *PPV*=Positive predictive value; *NPV*=Negative predictive value; *AUC*=Area under the receiver operator characteristic curve; *ICI*=integrated calibration index; *E50/E90/Emax*=50^th^/90^th^/max quantiles of difference between observed and optimal calibration curves

**Fig. 2:**
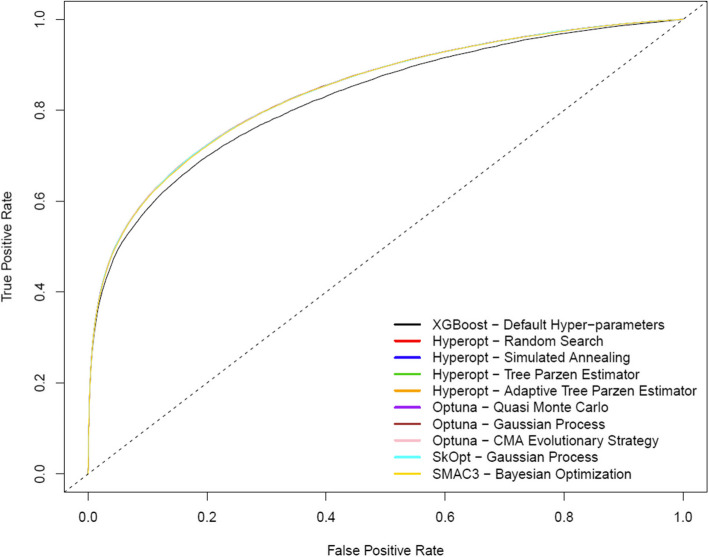
ROC curves for the optimal extreme gradient boosting classifiers for each hyper-parameter optimization method estimated on the 2017 test dataset

**Fig. 3:**
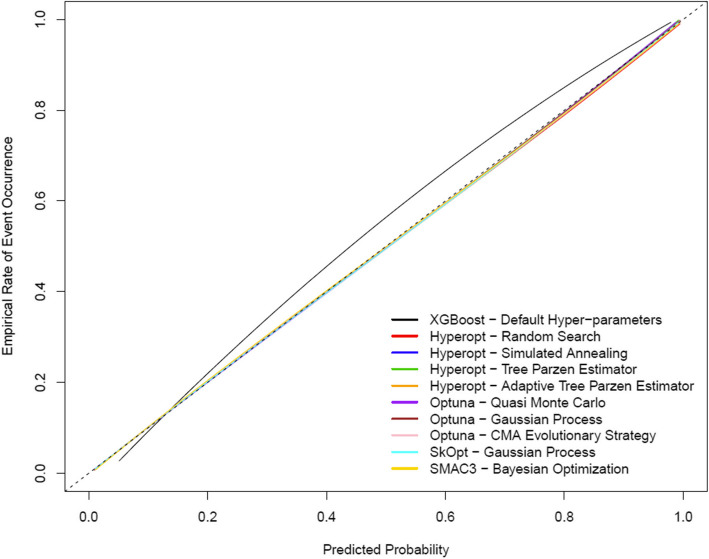
LOESS smoothed calibration curves for the optimal extreme gradient boosting classifiers for each hyper-parameter optimization method estimated on the 2017 test dataset

Figure 2 illustrates ROC curves for each hyper-parameter optimization method (n.b. the HPO methods result in overlapping/identical ROC curves). Figure 3 illustrates LOESS smoothed calibration curves for each hyper-parameter optimization method (n.b. the HPO methods result in overlapping/identical LOESS smoothed calibration curves). Following hyper-parameter optimization, calibration performance was near perfect as indicated by the near-zero values for ICI, E50, E90 and Emax indices (Table 3 and Fig. 3).

### Model discrimination and calibration performance on the 2019 test dataset

**Fig. 4:**
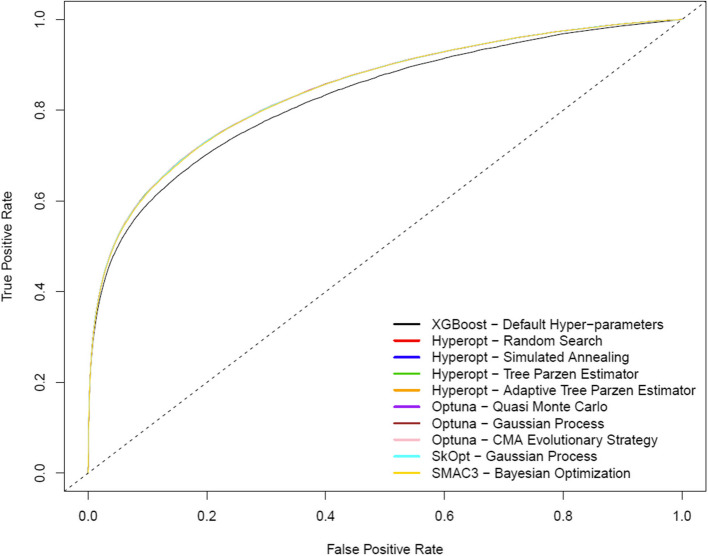
ROC curves for the optimal extreme gradient boosting classifiers for each hyper-parameter optimization method estimated on the 2019 test dataset

**Fig. 5:**
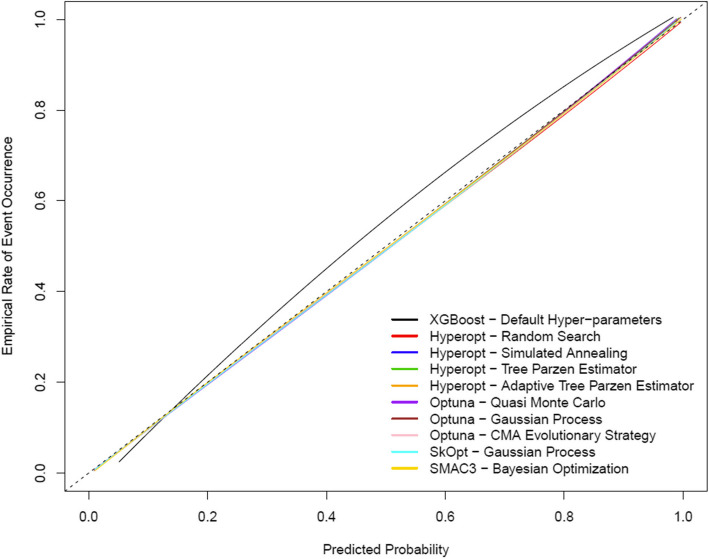
LOESS smoothed calibration curves for the optimal extreme gradient boosting classifiers for each hyper-parameter optimization method estimated on the 2019 test dataset

Table 4 summarizes model discrimination and calibration performance, estimated on the 2019 temporally independent test dataset (external validation). Figure 4 illustrates ROC curves for each hyper-parameter optimization method (again, each of the HPO methods result in identical/overlapping ROC curves). Figure 5 illustrates LOESS smoothed calibration curves for each hyper-parameter optimization method (again, each of the HPO methods result in identical/overlapping LOESS smoothed calibration curves).

**Table 4:** Discrimination and calibration performance estimated on the 2019 test dataset for each hyper-parameter optimization method

Hyper-Parameter Optimization Method	Acc	Sens	Spec	PPV	NPV	AUC	ICI	E50	E90	Emax
Default Hyper-parameters	0.891	0.355	0.984	0.798	0.898	0.824	0.027	0.030	0.038	0.061
Hyperopt - Random Search	0.894	0.42	0.977	0.759	0.906	0.845	0.003	0.003	0.005	0.008
Hyperopt - Simulated Annealing	0.894	0.419	0.977	0.759	0.906	0.845	0.003	0.003	0.004	0.007
Hyperopt - Tree Parzen Estimator	0.894	0.421	0.976	0.756	0.907	0.845	0.003	0.003	0.005	0.011
Hyperopt - Adaptive Tree Parzen Estimator	0.894	0.419	0.977	0.758	0.906	0.845	0.003	0.003	0.004	0.010
Optuna - Quasi Monte Carlo	0.894	0.42	0.977	0.758	0.907	0.845	0.004	0.003	0.007	0.013
Optuna - Gaussian Process	0.894	0.418	0.977	0.756	0.906	0.844	0.003	0.003	0.004	0.014
Optuna - Covariance Matrix Adaption Evolutionary Strategy	0.894	0.419	0.977	0.759	0.906	0.845	0.004	0.003	0.004	0.009
SkOpt - Gaussian Process	0.895	0.420	0.977	0.760	0.907	0.845	0.003	0.003	0.004	0.008
SMAC3 - Bayesian Optimization	0.894	0.419	0.977	0.758	0.906	0.844	0.003	0.003	0.005	0.008

*Acc*=Accuracy; *Sens*=Sensitivity; *Spec*=Specificity; *PPV*=Positive predictive value; *NPV*=Negative predictive value; *AUC*=Area under the receiver operator characteristic curve; *ICI*=integrated calibration index; *E50/E90/Emax*=50^th^/90^th^/max quantiles of difference between observed and optimal calibration curves

Findings with respect to discrimination and calibration performance were similar when estimated on the temporally independent 2019 test dataset (external validation), as compared to the 2017 test dataset (internal validation). Extreme gradient boosting models learned using each hyper-parameter optimization method on the 2019 test dataset had AUC= 0.84, and near-zero values for ICI, E50, E90 and Emax indices.

### XGBoost feature importance rankings from the 2017 train/validation datasets

We use the total gain metric to rank the importance of features to each extreme gradient boosting model (fit using different hyper-parameter optimization methods). We present the most important features learned by fitting an extreme gradient boosting model to the combined 2017 training/validation datasets (Table 5). We present the top- 10 most important features for predicting high-need high-cost health care users (based on average rank across models). A matrix of Kendall’s tau concordance statistics between ranked feature importance lists is presented in Table 6. The correlation statistics suggest stability in inferred feature importance lists across extreme gradient boosting models learning using different HPO methods.

**Table 5:** Feature importance rank statistics, associated with the top-10 most important features (based on average rank) for identifying high-need high-cost health care users, estimated by fitting independent extreme gradient boosting models using different hyper-parameter optimization methods

Feature Variable	Default Rank	RS Rank	SA Rank	TPE Rank	ATPE Rank	QMC Rank	GP Rank	CMAES Rank	SkOpt Rank	SMAC3 Rank
Long term care (in past 2 years)	1	1	1	1	1	1	1	1	1	1
Chronic home care (in past 2 years)	2	2	2	2	2	2	3	3	2	3
Dementia (diagnosed greater than 2 years ago)	5	3	3	3	3	3	2	2	6	2
Chronic home care while awaiting LTC (in past 2 years)	3	4	4	4	4	5	4	4	3	5
Chronic dialysis (in past 90 days)	11	5	5	5	5	4	5	5	4	4
Palliative care (in past 6 months)	13	6	6	6	6	6	6	6	5	6
Dementia (newly diagnosed)	14	8	7	7	9	9	8	8	18	8
Metastatic cancer (diagnosed in past 2 years)	7	9	10	9	8	8	17	10	8	12
HIV (diagnosed greater than 2 years ago)	16	7	8	10	7	7	18	7	7	11
Congestive heart failure (diagnosed greater than 2 years ago)	10	10	11	8	10	13	10	9	13	10

^*^Default: XGBoost model fit using default hyper-parameter values

^*^RS: XGBoost model fit using (hyperopt) random search hyper-parameter optimization method

^*^SA: XGBoost model fit using (hyperopt) simulated annealing hyper-parameter optimization method

^*^TPE: XGBoost model fit using (hyperopt) tree-Parzen estimator hyper-parameter optimization method

^*^ATPE: XGBoost model fit using (hyperopt) adaptive tree-Parzen estimator hyper-parameter optimization method

^*^QMC: XGBoost model fit using (optuna) quasi Monte Carlo hyper-parameter optimization method

^*^GP: XGBoost model fit using (optuna) Gaussian Process hyper-parameter optimization method

^*^CMAES: XGBoost model fit using (optuna) covariance matrix adaptation evolutionary strategy hyper-parameter optimization method

^*^SkOpt: XGBoost model fit using (SkOpt) Gaussian Process hyper-parameter optimization method

^*^SMAC3: XGBoost model fit using (SMAC3) random forest hyper-parameter optimization method

**Table 6:** Matrix of Kendall’s tau correlation statistics between feature importance rankings from extreme gradient boosting models learned using different hyper-parameter optimization methods

	Default	RS	SA	TPE	ATPE	QMC	GP	CMAES	SkOpt	SMAC3
Default	1	0.499	0.495	0.55	0.482	0.466	0.566	0.501	0.455	0.489
RS	0.499	1	0.913	0.864	0.922	0.772	0.717	0.886	0.772	0.802
SA	0.495	0.913	1	0.857	0.912	0.785	0.713	0.904	0.784	0.827
TPE	0.550	0.864	0.857	1	0.828	0.731	0.814	0.867	0.705	0.818
ATPE	0.482	0.922	0.912	0.828	1	0.774	0.677	0.868	0.803	0.787
QMC	0.466	0.772	0.785	0.731	0.774	1	0.671	0.768	0.817	0.770
GP	0.566	0.717	0.713	0.814	0.677	0.671	1	0.725	0.620	0.747
CMAES	0.501	0.886	0.904	0.867	0.868	0.768	0.725	1	0.765	0.858
SkOpt	0.455	0.772	0.784	0.705	0.803	0.817	0.620	0.765	1	0.765
SMAC3	0.489	0.802	0.827	0.818	0.787	0.770	0.747	0.858	0.765	1

^*^Default: XGBoost model fit using default hyper-parameter values

^*^RS: XGBoost model fit using (hyperopt) random search hyper-parameter optimization method

^*^SA: XGBoost model fit using (hyperopt) simulated annealing hyper-parameter optimization method

^*^TPE: XGBoost model fit using (hyperopt) tree-Parzen estimator hyper-parameter optimization method

^*^ATPE: XGBoost model fit using (hyperopt) adaptive tree-Parzen estimator hyper-parameter optimization method

^*^QMC: XGBoost model fit using (optuna) quasi Monte Carlo hyper-parameter optimization method

^*^GP: XGBoost model fit using (optuna) Gaussian Process hyper-parameter optimization method

^*^CMAES: XGBoost model fit using (optuna) covariance matrix adaptation evolutionary strategy hyper-parameter optimization method

^*^SkOpt: XGBoost model fit using (Skopt) Gaussian Process hyper-parameter optimization method

^*^SMAC3: XGBoost model fit using (SMAC3) random forest hyper-parameter optimization method

## Discussion

We compared the performance of several hyper-parameter optimization methods, used for tuning a binary extreme gradient boosting classification model, aimed at predicting high-need high-cost health care users. We observed that the different HPO methods all achieved similar performance in terms of discrimination and calibration metrics and yielded similar inferences in terms of important features identified by the models. This observation likely arises from certain unique characteristics of our study problem: 1) models are estimated and evaluated using large datasets, 2) the dataset consists of relatively few features compared to the number of observations/events, and 3) the dataset has a strong signal to-noise ratio resulting from fact that subject matter experts selected features they hypothesized to impact high-need high-cost status (e.g. socio-demographic characteristics, diagnoses with physical/mental health conditions, and previous hospitalizations and health service utilization). Our study adds to an existing machine learning literature, comparing HPO methods on real world datasets. The study also addresses a knowledge gap resulting from a paucity of HPO methods research, in the context of clinical predictive modelling (Supplementary Material 1).

Overall, the existing literature is mixed with respect to the impacts of HPO methods on the performance of binary prediction models. Bergstra et al. [6, 8] observed similar accuracy across HPO methods used for tuning SVM hyper-parameters on an HPOlib benchmark dataset. Putatunda et al. [21] used several benchmark datasets to demonstrate that hyperopt HPO methods identify more performant XGBoost models, compared to those identified using grid/random search methods. Shekhar et al. [22] compared HPO methods across several benchmark datasets, using different binary classification models, and demonstrated that the relative performance of HPO methods varied across datasets/models. That said, as the dataset size and number of features increased, differences between HPO methods were attenuated. Dunias et al. [13] investigated the relative performance HPO methods across several binary classification models (i.e. penalized logistic regression and random forest models), using a clinical tabular dataset with a small sample size and small number of feature variables. The researchers observed variability in predictive model performance resulting from different HPO methods and is one of the few studies focusing on HPO methods and clinical predictive models.

### Strengths and limitations

A strength of our study relates to the size of the dataset used for model training and evaluation. Inferences from previous comparative HPO methods studies are based on smaller datasets.

Our comparative evaluation study did not include all HPO/HPT frameworks. For example, we did not include ray-tune, Optunity, Nevergrad, and DEAP.

Several HPO methods included in this study themselves have hyper-parameters (sometimes referred to as meta-parameters). Our study has not reviewed the impact of meta-parameter tuning on model performance.

This study considered a single supervised machine learning model for predicting binary high-need high-cost health care user status - the extreme gradient boosting model. The extreme gradient boosting model has been shown to be performant across many prediction/classification problems, involving tabular feature data, and hence was a natural choice to use as a base model for our comparative HPO methods study. Comparison of different binary classification models was not the primary objective of this study. Hence, we do not include cross-model comparisons; rather, our study aimed to compare HPO methods holding model class fixed.

Our study prioritized AUC as a discrimination metric, functions of LOESS smooth calibration curves, and “total gain” based feature importance metrics. A strength of our study, like that of Dunias et al. [13], is that it evaluated the impact of HPO methods with respect to several complementary performance metrics relevant to clinical predictive modelling. Alternative metrics like F1-score, or balanced accuracy might be used for evaluating model discrimination. Similarly, SHAP values or LIME values could be used to assess stability of feature importances. Researchers should select performance metrics which best prioritize research goals (acknowledging differential costs of misclassifications, and miscalibration).

## Conclusions

Hyper-parameter optimization can be a critical component to developing performant clinical prediction models. Our study found that all HPO methods under investigation resulted in similar meaningful improvements in discrimination/calibration metrics relative to baseline models.

We hypothesize that this finding relates to having a large sample size, a small number of features, and a strong signal to noise ratio. Researchers building clinical prediction models using datasets that are smaller and having a larger number of features compared to the total sample size should explore the sensitivity of different hyper-parameter tuning methods on their study inferences.

## Supplementary Information

Supplementary Material 1.

Supplementary Material 2.

## Data Availability

The dataset from this study is held securely in coded form at ICES. While legal data sharing agreements between ICES and data providers (e.g. healthcare organizations and government) prohibit the institute from making the dataset publicly accessible, access may be granted to those who meet pre-specified criteria for confidential access, available at www.ices.on.ca (das@ices.on.ca).
