# Supplementary material for: Comparison of methods for tuning machine learning model hyper-parameters: with application to predicting high-need high-cost health care users
Source: BMC Med Res Methodol. 2025 May 15;25:134. doi: 10.1186/s12874-025-02561-x (PMC12083160; doi:10.1186/s12874-025-02561-x)
Supplement: Supplementary file 1 — Supplementary Material 1. [file 12874_2025_2561_MOESM1_ESM.html]

AppendixA\_QueryPubmed\_ClinPredModels\_HyperParamOptim\_7April2025


In [1]:

```
####################################################
## This script will use BeatifulSoup to query PubMed
## We will focus on two queries: 1) clinical predictive modelling, and 2) hyper-parameter optimization
## We will illustrate the explosion of research in clinical predictive modelling, and paucity of work in hyper-parameter optimization/tuning
##
## Author: Christopher Meaney
## Date: April 2025
####################################################
```

In [2]:

```
####################################################
## Package dependencies
####################################################

## Web scraping
import requests
from bs4 import BeautifulSoup

## Data wrangling
import pandas as pd

## Timing
import time

## Random number generation
import random
```

In [3]:

```
pd.set_option('display.max_rows', 250)
pd.set_option('display.max_columns', 50)
pd.set_option('display.max_colwidth', 250)
```

In [ ]:

```

```

In [ ]:

```

```

In [4]:

```
#####################################################
## Constants for constructing PubMed search string
#####################################################

## URL to PubMed
pubmed_url = "https://pubmed.ncbi.nlm.nih.gov/"

## Articles per page
articles_per_page = 100

## PubMed max page size (which is function articles per page)
max_display_pages = 1000
```

In [ ]:

```

```

In [ ]:

```

```

In [5]:

```
######################################################
## Define search query --- broadly focused on "clinical predictive models" and "supervised ML" in PubMed articles
######################################################
query1 = '''
        ("supervised ml" OR "supervised machine learning" OR "supervised learning" OR "binary classifier" OR "binary classification" OR "predictive model" OR "prediction model")
        AND ("2020/01/01"[PDAT] : "2025/03/31"[PDAT])
        '''
query1
```

Out[5]:

```
'\n        ("supervised ml" OR "supervised machine learning" OR "supervised learning" OR "binary classifier" OR "binary classification" OR "predictive model" OR "prediction model")\n        AND ("2020/01/01"[PDAT] : "2025/03/31"[PDAT])\n        '
```

In [6]:

```
## Construct PubMed search URL
search_url1 = f"{pubmed_url}?term={query1.replace(' ', '+')}&size={articles_per_page}"
search_url1
```

Out[6]:

```
'https://pubmed.ncbi.nlm.nih.gov/?term=\n++++++++("supervised+ml"+OR+"supervised+machine+learning"+OR+"supervised+learning"+OR+"binary+classifier"+OR+"binary+classification"+OR+"predictive+model"+OR+"prediction+model")\n++++++++AND+("2020/01/01"[PDAT]+:+"2025/03/31"[PDAT])\n++++++++&size=100'
```

In [7]:

```
## Post query to PubMed and see if it returns resposne
response1 = requests.get(search_url1, headers={'User-Agent': 'Mozilla/5.0'})
```

In [8]:

```
## Process returned result with Beautiful Soup HTML parser
soup1 = BeautifulSoup(response1.text, "html.parser")
```

In [9]:

```
## Get desired span element from result object
result_count_elem1 = soup1.find("span", class_="value")
result_count_elem1
```

Out[9]:

```
<span class="value">48,065</span>
```

In [10]:

```
## Grab total number of articles
total_articles1 = int(result_count_elem1.get_text(strip=True).replace(",", ""))
total_articles1
```

Out[10]:

```
48065
```

In [ ]:

```

```

In [ ]:

```

```

In [11]:

```
######################################################
## Define search query --- broadly focused on "clinical predictive models" and "supervised ML" in PubMed articles
## --- Narrow focus on logistic regression
######################################################
query1_logistic = '''("supervised ml" OR "supervised machine learning" OR "supervised learning" OR "binary classifier" OR "binary classification" OR "predictive model" OR "prediction model")
AND ("2020/01/01"[PDAT] : "2025/03/31"[PDAT]) 
AND ("logistic regression" OR "logistic glm" OR "logistic generalized linear model")'''
query1_logistic
```

Out[11]:

```
'("supervised ml" OR "supervised machine learning" OR "supervised learning" OR "binary classifier" OR "binary classification" OR "predictive model" OR "prediction model")\nAND ("2020/01/01"[PDAT] : "2025/03/31"[PDAT]) \nAND ("logistic regression" OR "logistic glm" OR "logistic generalized linear model")'
```

In [12]:

```
## Construct PubMed search URL
search_url1_logistic = f"{pubmed_url}?term={query1_logistic.replace(' ', '+')}&size={articles_per_page}"
search_url1_logistic
```

Out[12]:

```
'https://pubmed.ncbi.nlm.nih.gov/?term=("supervised+ml"+OR+"supervised+machine+learning"+OR+"supervised+learning"+OR+"binary+classifier"+OR+"binary+classification"+OR+"predictive+model"+OR+"prediction+model")\nAND+("2020/01/01"[PDAT]+:+"2025/03/31"[PDAT])+\nAND+("logistic+regression"+OR+"logistic+glm"+OR+"logistic+generalized+linear+model")&size=100'
```

In [13]:

```
## Post query to PubMed and see if it returns resposne
response1_logistic = requests.get(search_url1_logistic, headers={'User-Agent': 'Mozilla/5.0'})
```

In [14]:

```
## Process returned result with Beautiful Soup HTML parser
soup1_logistic = BeautifulSoup(response1_logistic.text, "html.parser")
```

In [15]:

```
## Get desired span element from result object
result_count_elem1_logistic = soup1_logistic.find("span", class_="value")
result_count_elem1_logistic
```

Out[15]:

```
<span class="value">10,607</span>
```

In [16]:

```
## Grab total number of articles
total_articles1_logistic = int(result_count_elem1_logistic.get_text(strip=True).replace(",", ""))
total_articles1_logistic
```

Out[16]:

```
10607
```

In [ ]:

```

```

In [ ]:

```

```

In [17]:

```
######################################################
## Define search query --- broadly focused on "clinical predictive models" and "supervised ML" in PubMed articles
## --- Narrow focus on extreme gradient boosting
######################################################
query1_xgboost = '''("supervised ml" OR "supervised machine learning" OR "supervised learning" OR "binary classifier" OR "binary classification" OR "predictive model" OR "prediction model")
AND ("2020/01/01"[PDAT] : "2025/03/31"[PDAT]) 
AND ("xgboost" OR "extreme gradient boosting")'''
query1_xgboost
```

Out[17]:

```
'("supervised ml" OR "supervised machine learning" OR "supervised learning" OR "binary classifier" OR "binary classification" OR "predictive model" OR "prediction model")\nAND ("2020/01/01"[PDAT] : "2025/03/31"[PDAT]) \nAND ("xgboost" OR "extreme gradient boosting")'
```

In [18]:

```
## Construct PubMed search URL
search_url1_xgboost = f"{pubmed_url}?term={query1_xgboost.replace(' ', '+')}&size={articles_per_page}"
search_url1_xgboost
```

Out[18]:

```
'https://pubmed.ncbi.nlm.nih.gov/?term=("supervised+ml"+OR+"supervised+machine+learning"+OR+"supervised+learning"+OR+"binary+classifier"+OR+"binary+classification"+OR+"predictive+model"+OR+"prediction+model")\nAND+("2020/01/01"[PDAT]+:+"2025/03/31"[PDAT])+\nAND+("xgboost"+OR+"extreme+gradient+boosting")&size=100'
```

In [19]:

```
## Post query to PubMed and see if it returns resposne
response1_xgboost = requests.get(search_url1_xgboost, headers={'User-Agent': 'Mozilla/5.0'})
```

In [20]:

```
## Process returned result with Beautiful Soup HTML parser
soup1_xgboost = BeautifulSoup(response1_xgboost.text, "html.parser")
```

In [21]:

```
## Get desired span element from result object
result_count_elem1_xgboost = soup1_xgboost.find("span", class_="value")
result_count_elem1_xgboost
```

Out[21]:

```
<span class="value">2,142</span>
```

In [22]:

```
## Grab total number of articles
total_articles1_xgboost = int(result_count_elem1_xgboost.get_text(strip=True).replace(",", ""))
total_articles1_xgboost
```

Out[22]:

```
2142
```

In [ ]:

```

```

In [ ]:

```

```

In [ ]:

```

```

In [23]:

```
######################################################
## Define search query --- narrowly focused on "clinical predictive models" and "HPO/HPT/AutoML" in PubMed articles
######################################################
query2 = '''("supervised ml" OR "supervised machine learning" OR "supervised learning" OR "binary classifier" OR "binary classification" OR "predictive model" OR "prediction model")
AND ("hyperparameter optimization" OR "hyper-parameter optimization" OR "hyper parameter tuning" OR "hyper-parameter tuning" OR "automl" OR "automated machine learning" OR "hyperopt" OR "optuna" OR "ray-tune" OR "ray tune" OR "skopt" OR "optunity" OR "smac3")
AND ("2020/01/01"[PDAT] : "2025/03/31"[PDAT])'''
query2
```

Out[23]:

```
'("supervised ml" OR "supervised machine learning" OR "supervised learning" OR "binary classifier" OR "binary classification" OR "predictive model" OR "prediction model")\nAND ("hyperparameter optimization" OR "hyper-parameter optimization" OR "hyper parameter tuning" OR "hyper-parameter tuning" OR "automl" OR "automated machine learning" OR "hyperopt" OR "optuna" OR "ray-tune" OR "ray tune" OR "skopt" OR "optunity" OR "smac3")\nAND ("2020/01/01"[PDAT] : "2025/03/31"[PDAT])'
```

In [24]:

```
## Construct PubMed search URL
search_url2 = f"{pubmed_url}?term={query2.replace(' ', '+')}&size={articles_per_page}"
search_url2
```

Out[24]:

```
'https://pubmed.ncbi.nlm.nih.gov/?term=("supervised+ml"+OR+"supervised+machine+learning"+OR+"supervised+learning"+OR+"binary+classifier"+OR+"binary+classification"+OR+"predictive+model"+OR+"prediction+model")\nAND+("hyperparameter+optimization"+OR+"hyper-parameter+optimization"+OR+"hyper+parameter+tuning"+OR+"hyper-parameter+tuning"+OR+"automl"+OR+"automated+machine+learning"+OR+"hyperopt"+OR+"optuna"+OR+"ray-tune"+OR+"ray+tune"+OR+"skopt"+OR+"optunity"+OR+"smac3")\nAND+("2020/01/01"[PDAT]+:+"2025/03/31"[PDAT])&size=100'
```

In [25]:

```
## Post query to PubMed and see if it returns resposne
response2 = requests.get(search_url2, headers={'User-Agent': 'Mozilla/5.0'})
```

In [26]:

```
## Process returned result with Beautiful Soup HTML parser
soup2 = BeautifulSoup(response2.text, "html.parser")
```

In [27]:

```
## Get desired span element from result object
result_count_elem2 = soup2.find("span", class_="value")
result_count_elem2
```

Out[27]:

```
<span class="value">208</span>
```

In [28]:

```
## Grab total number of articles
total_articles2 = int(result_count_elem2.get_text(strip=True).replace(",", ""))
total_articles2
```

Out[28]:

```
208
```

In [ ]:

```

```

In [ ]:

```

```

In [29]:

```
######################################################
## Define search query --- narrowly focused on "clinical predictive models" and "HPO/HPT/AutoML" in PubMed articles
## --- Focus on XGBoost models and HPO
######################################################
query2_xgboost= '''("supervised ml" OR "supervised machine learning" OR "supervised learning" OR "binary classifier" OR "binary classification" OR "predictive model" OR "prediction model")
AND ("hyperparameter optimization" OR "hyper-parameter optimization" OR "hyper parameter tuning" OR "hyper-parameter tuning" OR "automl" OR "automated machine learning" OR "hyperopt" OR "optuna" OR "ray-tune" OR "ray tune" OR "skopt" OR "optunity" OR "smac3")
AND ("2020/01/01"[PDAT] : "2025/03/31"[PDAT])
AND ("xgboost" OR "extreme gradient boosting")'''
query2_xgboost
```

Out[29]:

```
'("supervised ml" OR "supervised machine learning" OR "supervised learning" OR "binary classifier" OR "binary classification" OR "predictive model" OR "prediction model")\nAND ("hyperparameter optimization" OR "hyper-parameter optimization" OR "hyper parameter tuning" OR "hyper-parameter tuning" OR "automl" OR "automated machine learning" OR "hyperopt" OR "optuna" OR "ray-tune" OR "ray tune" OR "skopt" OR "optunity" OR "smac3")\nAND ("2020/01/01"[PDAT] : "2025/03/31"[PDAT])\nAND ("xgboost" OR "extreme gradient boosting")'
```

In [30]:

```
## Construct PubMed search URL
search_url2_xgboost = f"{pubmed_url}?term={query2_xgboost.replace(' ', '+')}&size={articles_per_page}"
search_url2_xgboost
```

Out[30]:

```
'https://pubmed.ncbi.nlm.nih.gov/?term=("supervised+ml"+OR+"supervised+machine+learning"+OR+"supervised+learning"+OR+"binary+classifier"+OR+"binary+classification"+OR+"predictive+model"+OR+"prediction+model")\nAND+("hyperparameter+optimization"+OR+"hyper-parameter+optimization"+OR+"hyper+parameter+tuning"+OR+"hyper-parameter+tuning"+OR+"automl"+OR+"automated+machine+learning"+OR+"hyperopt"+OR+"optuna"+OR+"ray-tune"+OR+"ray+tune"+OR+"skopt"+OR+"optunity"+OR+"smac3")\nAND+("2020/01/01"[PDAT]+:+"2025/03/31"[PDAT])\nAND+("xgboost"+OR+"extreme+gradient+boosting")&size=100'
```

In [31]:

```
## Post query to PubMed and see if it returns resposne
response2_xgboost = requests.get(search_url2_xgboost, headers={'User-Agent': 'Mozilla/5.0'})
```

In [32]:

```
## Process returned result with Beautiful Soup HTML parser
soup2_xgboost = BeautifulSoup(response2_xgboost.text, "html.parser")
```

In [33]:

```
## Get desired span element from result object
result_count_elem2_xgboost = soup2_xgboost.find("span", class_="value")
result_count_elem2_xgboost
```

Out[33]:

```
<span class="value">25</span>
```

In [34]:

```
## Grab total number of articles
total_articles2_xgboost = int(result_count_elem2_xgboost.get_text(strip=True).replace(",", ""))
total_articles2_xgboost
```

Out[34]:

```
25
```

In [ ]:

```

```

In [ ]:

```

```

In [ ]:

```

```

In [ ]:

```

```

In [35]:

```
######################################################
## Only a small proportion of supervised-ML/clinical-predictive-model articles are about HPO/HPT/AutoML
######################################################
```

In [36]:

```
[total_articles1, total_articles2, (total_articles2/total_articles1)*100]
```

Out[36]:

```
[48065, 208, 0.4327473213356912]
```

In [37]:

```
[total_articles1_xgboost, total_articles2_xgboost, (total_articles2_xgboost/total_articles1_xgboost)*100]
```

Out[37]:

```
[2142, 25, 1.1671335200746966]
```

In [ ]:

```

```

In [ ]:

```

```

In [ ]:

```

```

In [38]:

```
#######################################################
## Get corpora of documents corresponding to Pubmed searches --- total_articles2 and total_articles2_xgboost
#######################################################
```

In [39]:

```
## Second/narrower PubMed search query
query2 = '''("supervised ml" OR "supervised machine learning" OR "supervised learning" OR "binary classifier" OR "binary classification" OR "predictive model" OR "prediction model") 
AND ("hyperparameter optimization" OR "hyper-parameter optimization" OR "hyper parameter tuning" OR "hyper-parameter tuning" OR "automl" OR "automated machine learning" OR "hyperopt" OR "optuna" OR "ray-tune" OR "ray tune" OR "skopt" OR "optunity" OR "smac3")
AND ("2020/01/01"[PDAT] : "2025/03/31"[PDAT])
'''
query2
```

Out[39]:

```
'("supervised ml" OR "supervised machine learning" OR "supervised learning" OR "binary classifier" OR "binary classification" OR "predictive model" OR "prediction model") \nAND ("hyperparameter optimization" OR "hyper-parameter optimization" OR "hyper parameter tuning" OR "hyper-parameter tuning" OR "automl" OR "automated machine learning" OR "hyperopt" OR "optuna" OR "ray-tune" OR "ray tune" OR "skopt" OR "optunity" OR "smac3")\nAND ("2020/01/01"[PDAT] : "2025/03/31"[PDAT])\n'
```

In [40]:

```
## Construct base URL to PubMed (based on search query2 defined above)
pubmed_url = "https://pubmed.ncbi.nlm.nih.gov/"
search_url = f"{pubmed_url}?term={query2.replace(' ', '+')}&size=100"
search_url
```

Out[40]:

```
'https://pubmed.ncbi.nlm.nih.gov/?term=("supervised+ml"+OR+"supervised+machine+learning"+OR+"supervised+learning"+OR+"binary+classifier"+OR+"binary+classification"+OR+"predictive+model"+OR+"prediction+model")+\nAND+("hyperparameter+optimization"+OR+"hyper-parameter+optimization"+OR+"hyper+parameter+tuning"+OR+"hyper-parameter+tuning"+OR+"automl"+OR+"automated+machine+learning"+OR+"hyperopt"+OR+"optuna"+OR+"ray-tune"+OR+"ray+tune"+OR+"skopt"+OR+"optunity"+OR+"smac3")\nAND+("2020/01/01"[PDAT]+:+"2025/03/31"[PDAT])\n&size=100'
```

In [41]:

```
##
## User Defined Function to Extract URLs from initial PubMed search
##
def get_article_urls(search_url):
    ## Instantitate list to hold
    article_urls = []
    ## Set page counter to 1 (for first page in search)
    page = 1
    ## Loop over pages/URLs and store in list
    while True:
        ## Search URL based on query2 above
        url = f"{search_url}&page={page}"
        ## Grab response
        response = requests.get(url)
        ## Handle errors
        if response.status_code != 200:
            break
        ## Scrape text from returned page
        soup = BeautifulSoup(response.text, 'html.parser')
        ## Get articles from parsed HTML
        articles = soup.select(".docsum-title")
        ## Stop if no more articles
        if not articles:
            break  
        ## Construct URLs from article (for given page)
        for article in articles:
            article_url = pubmed_url + article['href']
            article_urls.append(article_url)
        ## Increment pages
        page += 1
        ## Sleep to not crash process
        time.sleep(random.uniform(0, 2))
    ## Return list of URLs to user
    return article_urls
```

In [42]:

```
## Gather articles for scraping
article_urls = get_article_urls(search_url=search_url)
len(article_urls)
```

Out[42]:

```
208
```

In [43]:

```
## Print articles URLs to console
article_urls
```

Out[43]:

```
['https://pubmed.ncbi.nlm.nih.gov//37698911/',
 'https://pubmed.ncbi.nlm.nih.gov//36312291/',
 'https://pubmed.ncbi.nlm.nih.gov//39300720/',
 'https://pubmed.ncbi.nlm.nih.gov//37789305/',
 'https://pubmed.ncbi.nlm.nih.gov//36362493/',
 'https://pubmed.ncbi.nlm.nih.gov//36845729/',
 'https://pubmed.ncbi.nlm.nih.gov//37166278/',
 'https://pubmed.ncbi.nlm.nih.gov//35161928/',
 'https://pubmed.ncbi.nlm.nih.gov//37384597/',
 'https://pubmed.ncbi.nlm.nih.gov//37789357/',
 'https://pubmed.ncbi.nlm.nih.gov//36553069/',
 'https://pubmed.ncbi.nlm.nih.gov//36149937/',
 'https://pubmed.ncbi.nlm.nih.gov//37350896/',
 'https://pubmed.ncbi.nlm.nih.gov//34793297/',
 'https://pubmed.ncbi.nlm.nih.gov//38212745/',
 'https://pubmed.ncbi.nlm.nih.gov//34972161/',
 'https://pubmed.ncbi.nlm.nih.gov//35885909/',
 'https://pubmed.ncbi.nlm.nih.gov//34684004/',
 'https://pubmed.ncbi.nlm.nih.gov//33977129/',
 'https://pubmed.ncbi.nlm.nih.gov//38132215/',
 'https://pubmed.ncbi.nlm.nih.gov//39427930/',
 'https://pubmed.ncbi.nlm.nih.gov//34344669/',
 'https://pubmed.ncbi.nlm.nih.gov//35429810/',
 'https://pubmed.ncbi.nlm.nih.gov//36303841/',
 'https://pubmed.ncbi.nlm.nih.gov//36279027/',
 'https://pubmed.ncbi.nlm.nih.gov//37947168/',
 'https://pubmed.ncbi.nlm.nih.gov//34912282/',
 'https://pubmed.ncbi.nlm.nih.gov//36634878/',
 'https://pubmed.ncbi.nlm.nih.gov//34288435/',
 'https://pubmed.ncbi.nlm.nih.gov//33065493/',
 'https://pubmed.ncbi.nlm.nih.gov//38414794/',
 'https://pubmed.ncbi.nlm.nih.gov//38723336/',
 'https://pubmed.ncbi.nlm.nih.gov//34270917/',
 'https://pubmed.ncbi.nlm.nih.gov//35691579/',
 'https://pubmed.ncbi.nlm.nih.gov//37034352/',
 'https://pubmed.ncbi.nlm.nih.gov//38034896/',
 'https://pubmed.ncbi.nlm.nih.gov//33074897/',
 'https://pubmed.ncbi.nlm.nih.gov//36673172/',
 'https://pubmed.ncbi.nlm.nih.gov//35806843/',
 'https://pubmed.ncbi.nlm.nih.gov//31905134/',
 'https://pubmed.ncbi.nlm.nih.gov//35955301/',
 'https://pubmed.ncbi.nlm.nih.gov//34600172/',
 'https://pubmed.ncbi.nlm.nih.gov//35885865/',
 'https://pubmed.ncbi.nlm.nih.gov//39386877/',
 'https://pubmed.ncbi.nlm.nih.gov//38871176/',
 'https://pubmed.ncbi.nlm.nih.gov//34189530/',
 'https://pubmed.ncbi.nlm.nih.gov//35122132/',
 'https://pubmed.ncbi.nlm.nih.gov//39928609/',
 'https://pubmed.ncbi.nlm.nih.gov//33439834/',
 'https://pubmed.ncbi.nlm.nih.gov//37332920/',
 'https://pubmed.ncbi.nlm.nih.gov//37162892/',
 'https://pubmed.ncbi.nlm.nih.gov//36050474/',
 'https://pubmed.ncbi.nlm.nih.gov//40155850/',
 'https://pubmed.ncbi.nlm.nih.gov//39140793/',
 'https://pubmed.ncbi.nlm.nih.gov//39011494/',
 'https://pubmed.ncbi.nlm.nih.gov//36268149/',
 'https://pubmed.ncbi.nlm.nih.gov//33100428/',
 'https://pubmed.ncbi.nlm.nih.gov//39644793/',
 'https://pubmed.ncbi.nlm.nih.gov//39583807/',
 'https://pubmed.ncbi.nlm.nih.gov//35368915/',
 'https://pubmed.ncbi.nlm.nih.gov//37943499/',
 'https://pubmed.ncbi.nlm.nih.gov//35585733/',
 'https://pubmed.ncbi.nlm.nih.gov//35660327/',
 'https://pubmed.ncbi.nlm.nih.gov//33375939/',
 'https://pubmed.ncbi.nlm.nih.gov//31165141/',
 'https://pubmed.ncbi.nlm.nih.gov//36807314/',
 'https://pubmed.ncbi.nlm.nih.gov//33539308/',
 'https://pubmed.ncbi.nlm.nih.gov//38690313/',
 'https://pubmed.ncbi.nlm.nih.gov//37250024/',
 'https://pubmed.ncbi.nlm.nih.gov//38825704/',
 'https://pubmed.ncbi.nlm.nih.gov//38489814/',
 'https://pubmed.ncbi.nlm.nih.gov//37350880/',
 'https://pubmed.ncbi.nlm.nih.gov//39304867/',
 'https://pubmed.ncbi.nlm.nih.gov//39349571/',
 'https://pubmed.ncbi.nlm.nih.gov//39278905/',
 'https://pubmed.ncbi.nlm.nih.gov//32998684/',
 'https://pubmed.ncbi.nlm.nih.gov//38879852/',
 'https://pubmed.ncbi.nlm.nih.gov//34384203/',
 'https://pubmed.ncbi.nlm.nih.gov//35592648/',
 'https://pubmed.ncbi.nlm.nih.gov//39332687/',
 'https://pubmed.ncbi.nlm.nih.gov//38077132/',
 'https://pubmed.ncbi.nlm.nih.gov//38633305/',
 'https://pubmed.ncbi.nlm.nih.gov//39663141/',
 'https://pubmed.ncbi.nlm.nih.gov//36230158/',
 'https://pubmed.ncbi.nlm.nih.gov//35831804/',
 'https://pubmed.ncbi.nlm.nih.gov//36255582/',
 'https://pubmed.ncbi.nlm.nih.gov//38413923/',
 'https://pubmed.ncbi.nlm.nih.gov//37918734/',
 'https://pubmed.ncbi.nlm.nih.gov//39728286/',
 'https://pubmed.ncbi.nlm.nih.gov//37221231/',
 'https://pubmed.ncbi.nlm.nih.gov//32962113/',
 'https://pubmed.ncbi.nlm.nih.gov//37845674/',
 'https://pubmed.ncbi.nlm.nih.gov//38748753/',
 'https://pubmed.ncbi.nlm.nih.gov//38801342/',
 'https://pubmed.ncbi.nlm.nih.gov//37924888/',
 'https://pubmed.ncbi.nlm.nih.gov//39419638/',
 'https://pubmed.ncbi.nlm.nih.gov//32386536/',
 'https://pubmed.ncbi.nlm.nih.gov//37856110/',
 'https://pubmed.ncbi.nlm.nih.gov//36050434/',
 'https://pubmed.ncbi.nlm.nih.gov//38344615/',
 'https://pubmed.ncbi.nlm.nih.gov//35890310/',
 'https://pubmed.ncbi.nlm.nih.gov//33918195/',
 'https://pubmed.ncbi.nlm.nih.gov//36523293/',
 'https://pubmed.ncbi.nlm.nih.gov//34960494/',
 'https://pubmed.ncbi.nlm.nih.gov//36363023/',
 'https://pubmed.ncbi.nlm.nih.gov//36921349/',
 'https://pubmed.ncbi.nlm.nih.gov//32265477/',
 'https://pubmed.ncbi.nlm.nih.gov//33748592/',
 'https://pubmed.ncbi.nlm.nih.gov//36972645/',
 'https://pubmed.ncbi.nlm.nih.gov//38997304/',
 'https://pubmed.ncbi.nlm.nih.gov//39001328/',
 'https://pubmed.ncbi.nlm.nih.gov//34111437/',
 'https://pubmed.ncbi.nlm.nih.gov//39527213/',
 'https://pubmed.ncbi.nlm.nih.gov//34388470/',
 'https://pubmed.ncbi.nlm.nih.gov//39889299/',
 'https://pubmed.ncbi.nlm.nih.gov//38264719/',
 'https://pubmed.ncbi.nlm.nih.gov//34224056/',
 'https://pubmed.ncbi.nlm.nih.gov//37512636/',
 'https://pubmed.ncbi.nlm.nih.gov//37375934/',
 'https://pubmed.ncbi.nlm.nih.gov//37394932/',
 'https://pubmed.ncbi.nlm.nih.gov//35522787/',
 'https://pubmed.ncbi.nlm.nih.gov//34255713/',
 'https://pubmed.ncbi.nlm.nih.gov//39779935/',
 'https://pubmed.ncbi.nlm.nih.gov//36386374/',
 'https://pubmed.ncbi.nlm.nih.gov//36980475/',
 'https://pubmed.ncbi.nlm.nih.gov//39088983/',
 'https://pubmed.ncbi.nlm.nih.gov//38487294/',
 'https://pubmed.ncbi.nlm.nih.gov//36744032/',
 'https://pubmed.ncbi.nlm.nih.gov//39070227/',
 'https://pubmed.ncbi.nlm.nih.gov//37725103/',
 'https://pubmed.ncbi.nlm.nih.gov//38919392/',
 'https://pubmed.ncbi.nlm.nih.gov//36457192/',
 'https://pubmed.ncbi.nlm.nih.gov//37787655/',
 'https://pubmed.ncbi.nlm.nih.gov//39863646/',
 'https://pubmed.ncbi.nlm.nih.gov//36199778/',
 'https://pubmed.ncbi.nlm.nih.gov//35453869/',
 'https://pubmed.ncbi.nlm.nih.gov//38578764/',
 'https://pubmed.ncbi.nlm.nih.gov//35749336/',
 'https://pubmed.ncbi.nlm.nih.gov//37742704/',
 'https://pubmed.ncbi.nlm.nih.gov//38937503/',
 'https://pubmed.ncbi.nlm.nih.gov//32453457/',
 'https://pubmed.ncbi.nlm.nih.gov//33418465/',
 'https://pubmed.ncbi.nlm.nih.gov//38133687/',
 'https://pubmed.ncbi.nlm.nih.gov//40144877/',
 'https://pubmed.ncbi.nlm.nih.gov//34184998/',
 'https://pubmed.ncbi.nlm.nih.gov//39650439/',
 'https://pubmed.ncbi.nlm.nih.gov//39633817/',
 'https://pubmed.ncbi.nlm.nih.gov//34548227/',
 'https://pubmed.ncbi.nlm.nih.gov//38660216/',
 'https://pubmed.ncbi.nlm.nih.gov//33940479/',
 'https://pubmed.ncbi.nlm.nih.gov//32305942/',
 'https://pubmed.ncbi.nlm.nih.gov//34813465/',
 'https://pubmed.ncbi.nlm.nih.gov//37037836/',
 'https://pubmed.ncbi.nlm.nih.gov//38683712/',
 'https://pubmed.ncbi.nlm.nih.gov//35297826/',
 'https://pubmed.ncbi.nlm.nih.gov//39830784/',
 'https://pubmed.ncbi.nlm.nih.gov//38886757/',
 'https://pubmed.ncbi.nlm.nih.gov//34482200/',
 'https://pubmed.ncbi.nlm.nih.gov//36131178/',
 'https://pubmed.ncbi.nlm.nih.gov//39866196/',
 'https://pubmed.ncbi.nlm.nih.gov//34820296/',
 'https://pubmed.ncbi.nlm.nih.gov//39687913/',
 'https://pubmed.ncbi.nlm.nih.gov//37563894/',
 'https://pubmed.ncbi.nlm.nih.gov//38038910/',
 'https://pubmed.ncbi.nlm.nih.gov//33959972/',
 'https://pubmed.ncbi.nlm.nih.gov//33611749/',
 'https://pubmed.ncbi.nlm.nih.gov//35756658/',
 'https://pubmed.ncbi.nlm.nih.gov//36631333/',
 'https://pubmed.ncbi.nlm.nih.gov//33816947/',
 'https://pubmed.ncbi.nlm.nih.gov//37669779/',
 'https://pubmed.ncbi.nlm.nih.gov//33601166/',
 'https://pubmed.ncbi.nlm.nih.gov//34746553/',
 'https://pubmed.ncbi.nlm.nih.gov//39705459/',
 'https://pubmed.ncbi.nlm.nih.gov//38850438/',
 'https://pubmed.ncbi.nlm.nih.gov//33693377/',
 'https://pubmed.ncbi.nlm.nih.gov//39856375/',
 'https://pubmed.ncbi.nlm.nih.gov//38975289/',
 'https://pubmed.ncbi.nlm.nih.gov//38473015/',
 'https://pubmed.ncbi.nlm.nih.gov//33057581/',
 'https://pubmed.ncbi.nlm.nih.gov//34514033/',
 'https://pubmed.ncbi.nlm.nih.gov//33370240/',
 'https://pubmed.ncbi.nlm.nih.gov//38755269/',
 'https://pubmed.ncbi.nlm.nih.gov//38822411/',
 'https://pubmed.ncbi.nlm.nih.gov//37609808/',
 'https://pubmed.ncbi.nlm.nih.gov//40038107/',
 'https://pubmed.ncbi.nlm.nih.gov//37555812/',
 'https://pubmed.ncbi.nlm.nih.gov//38274412/',
 'https://pubmed.ncbi.nlm.nih.gov//37237033/',
 'https://pubmed.ncbi.nlm.nih.gov//38627439/',
 'https://pubmed.ncbi.nlm.nih.gov//34901796/',
 'https://pubmed.ncbi.nlm.nih.gov//32575475/',
 'https://pubmed.ncbi.nlm.nih.gov//39682641/',
 'https://pubmed.ncbi.nlm.nih.gov//39673942/',
 'https://pubmed.ncbi.nlm.nih.gov//39254891/',
 'https://pubmed.ncbi.nlm.nih.gov//39852326/',
 'https://pubmed.ncbi.nlm.nih.gov//40072485/',
 'https://pubmed.ncbi.nlm.nih.gov//39075434/',
 'https://pubmed.ncbi.nlm.nih.gov//39170161/',
 'https://pubmed.ncbi.nlm.nih.gov//38357602/',
 'https://pubmed.ncbi.nlm.nih.gov//39901185/',
 'https://pubmed.ncbi.nlm.nih.gov//39275500/',
 'https://pubmed.ncbi.nlm.nih.gov//35455141/',
 'https://pubmed.ncbi.nlm.nih.gov//38112602/',
 'https://pubmed.ncbi.nlm.nih.gov//38260372/',
 'https://pubmed.ncbi.nlm.nih.gov//39979708/',
 'https://pubmed.ncbi.nlm.nih.gov//34749632/',
 'https://pubmed.ncbi.nlm.nih.gov//33081986/',
 'https://pubmed.ncbi.nlm.nih.gov//39905008/']
```

In [ ]:

```

```

In [ ]:

```

```

In [44]:

```
##
## User defined function to extract bibliometric info from PubMed articles (input is URL, output is dictionary of bibliometric info)
##
def scrape_article(url):
    ## Grab response from PubMed article URL
    response = requests.get(url)
    ## Handle errors
    if response.status_code != 200:
        return None
    ## Parse HTML text
    soup = BeautifulSoup(response.text, 'html.parser')
    ## Grab article meta-data 
    title = soup.select_one("h1.heading-title").text.strip() if soup.select_one("h1.heading-title") else None
    abstract = soup.select_one("div.abstract-content").text.strip() if soup.select_one("div.abstract-content") else None
    pubdate = soup.select_one(".cit").text.strip() if soup.select_one(".cit") else None
    journal = soup.select_one("button.journal-actions-trigger").text.strip() if soup.select_one("button.journal-actions-trigger") else None
    authors = [a.text.strip() for a in soup.select(".authors-list .full-name")]
    doi = soup.select_one(".identifier.doi").text.strip() if soup.select_one(".identifier.doi") else None
    ## Return article meta-data to user
    return {
        "title": title,
        "abstract": abstract,
        "publication_date": pubdate,
        "journal": journal,
        "authors": "; ".join(authors),
        "doi": doi,
        "url": url
    }
```

In [45]:

```
## Instantiate list to hold bibliometric data from each article
articles_data = []
```

In [46]:

```
## Loop over article URL in list and generate bibliometric data
t0 = time.time()

for url in article_urls:
    ## Grab article info
    article_info = scrape_article(url)
    ## Append to list if available
    if article_info:
        articles_data.append(article_info)
    ## Random sleep to respect pubMed scraping rate limits
    time.sleep(random.uniform(0, 1))  

t1 = time.time()
t1-t0
```

Out[46]:

```
318.1103115081787
```

In [47]:

```
## Convert list of Dicts object above into a pandas DataFrame
df = pd.DataFrame(articles_data)
df.columns = ["title","abstract","publication_date","journal","authors","doi","url"]
df
```

Out[47]:

|  | title | abstract | publication\_date | journal | authors | doi | url |
| --- | --- | --- | --- | --- | --- | --- | --- |
| 0 | Predicting the 5-Year Risk of Nonalcoholic Fatty Liver Disease Using Machine Learning Models: Prospective Cohort Study | Background:\n \n \n Nonalcoholic fatty liver disease (NAFLD) has emerged as a worldwide public health issue. Identifying and targeting populations at a heightened risk of developing NAFLD over a 5-year period can help reduce and ... | 2023 Sep 12:25:e46891. | J Med Internet Res | Guoqing Huang; Qiankai Jin; Yushan Mao; Guoqing Huang; Guoqing Huang; Qiankai Jin; Yushan Mao | DOI:\n \n \n\n 10.2196/46891 | https://pubmed.ncbi.nlm.nih.gov//37698911/ |
| 1 | Interpretable machine learning for 28-day all-cause in-hospital mortality prediction in critically ill patients with heart failure combined with hypertension: A retrospective cohort study based on medical information mart for intensive care datab... | Background:\n \n \n Heart failure (HF) combined with hypertension is an extremely important cause of in-hospital mortality, especially for the intensive care unit (ICU) patients. However, under intense working pressure, the medic... | 2022 Oct 12:9:994359. | Front Cardiovasc Med | Shengxian Peng; Jian Huang; Xiaozhu Liu; Jiewen Deng; Chenyu Sun; Juan Tang; Huaqiao Chen; Wenzhai Cao; Wei Wang; Xiangjie Duan; Xianglin Luo; Shuang Peng; Shengxian Peng; Shengxian Peng; Jian Huang; Xiaozhu Liu; Jiewen Deng; Chenyu Sun; Juan Tan... | DOI:\n \n \n\n 10.3389/fcvm.2022.994359 | https://pubmed.ncbi.nlm.nih.gov//36312291/ |
| 2 | Automated machine learning with R: AutoML tools for beginners in clinical research | Recently, interest in machine learning (ML) has increased as the application fields have expanded significantly. Although ML methods excel in many fields, establishing an ML pipeline requires considerable time and human resources. Automated ML (A... | 2024 Sep 15;27(3):129-137. | J Minim Invasive Surg | Youngho Park; Youngho Park; Youngho Park | DOI:\n \n \n\n 10.7602/jmis.2024.27.3.129 | https://pubmed.ncbi.nlm.nih.gov//39300720/ |
| 3 | Machine learning-based prediction model of acute kidney injury in patients with acute respiratory distress syndrome | Background:\n \n \n Acute kidney injury (AKI) can make cases of acute respiratory distress syndrome (ARDS) more complex, and the combination of the two can significantly worsen the prognosis. Our objective is to utilize machine l... | 2023 Oct 3;23(1):370. | BMC Pulm Med | Shuxing Wei; Yongsheng Zhang; Hongmeng Dong; Ying Chen; Xiya Wang; Xiaomei Zhu; Guang Zhang; Shubin Guo; Shuxing Wei; Shuxing Wei; Yongsheng Zhang; Hongmeng Dong; Ying Chen; Xiya Wang; Xiaomei Zhu; Guang Zhang; Shubin Guo | DOI:\n \n \n\n 10.1186/s12890-023-02663-6 | https://pubmed.ncbi.nlm.nih.gov//37789305/ |
| 4 | Explainable Preoperative Automated Machine Learning Prediction Model for Cardiac Surgery-Associated Acute Kidney Injury | Background:\n \n \n We aimed to develop and validate an automated machine learning (autoML) prediction model for cardiac surgery-associated acute kidney injury (CSA-AKI).\n \n\n\n Methods:\n \n \n Usi... | 2022 Oct 24;11(21):6264. | J Clin Med | Charat Thongprayoon; Pattharawin Pattharanitima; Andrea G Kattah; Michael A Mao; Mira T Keddis; John J Dillon; Wisit Kaewput; Supawit Tangpanithandee; Pajaree Krisanapan; Fawad Qureshi; Wisit Cheungpasitporn; Charat Thongprayoon; Charat Thongpray... | DOI:\n \n \n\n 10.3390/jcm11216264 | https://pubmed.ncbi.nlm.nih.gov//36362493/ |
| 5 | Common statistical concepts in the supervised Machine Learning arena | One of the core elements of Machine Learning (ML) is statistics and its embedded foundational rules and without its appropriate integration, ML as we know would not exist. Various aspects of ML platforms are based on statistical rules and most no... | 2023 Feb 14:13:1130229. | Front Oncol | Hooman H Rashidi; Samer Albahra; Scott Robertson; Nam K Tran; Bo Hu; Hooman H Rashidi; Hooman H Rashidi; Samer Albahra; Scott Robertson; Nam K Tran; Bo Hu | DOI:\n \n \n\n 10.3389/fonc.2023.1130229 | https://pubmed.ncbi.nlm.nih.gov//36845729/ |
| 6 | Automated Motor Tic Detection: A Machine Learning Approach | Background:\n \n \n Video-based tic detection and scoring is useful to independently and objectively assess tic frequency and severity in patients with Tourette syndrome. In trained raters, interrater reliability is good. However... | 2023 Jul;38(7):1327-1335. | Mov Disord | Nele Sophie Brügge; Gesine Marie Sallandt; Ronja Schappert; Frédéric Li; Alina Siekmann; Marcin Grzegorzek; Tobias Bäumer; Christian Frings; Christian Beste; Roland Stenger; Veit Roessner; Sebastian Fudickar; Heinz Handels; Alexander Münchau; Nel... | DOI:\n \n \n\n 10.1002/mds.29439 | https://pubmed.ncbi.nlm.nih.gov//37166278/ |
| 7 | Multi-Label Active Learning-Based Machine Learning Model for Heart Disease Prediction | The rapid growth and adaptation of medical information to identify significant health trends and help with timely preventive care have been recent hallmarks of the modern healthcare data system. Heart disease is the deadliest condition in the dev... | 2022 Feb 4;22(3):1184. | Sensors (Basel) | Ibrahim M El-Hasnony; Omar M Elzeki; Ali Alshehri; Hanaa Salem; Ibrahim M El-Hasnony; Ibrahim M El-Hasnony; Omar M Elzeki; Ali Alshehri; Hanaa Salem | DOI:\n \n \n\n 10.3390/s22031184 | https://pubmed.ncbi.nlm.nih.gov//35161928/ |
| 8 | Machine Learning in Environmental Research: Common Pitfalls and Best Practices | Machine learning (ML) is increasingly used in environmental research to process large data sets and decipher complex relationships between system variables. However, due to the lack of familiarity and methodological rigor, inadequate ML studies m... | 2023 Nov 21;57(46):17671-17689. | Environ Sci Technol | Jun-Jie Zhu; Meiqi Yang; Zhiyong Jason Ren; Jun-Jie Zhu; Jun-Jie Zhu; Meiqi Yang; Zhiyong Jason Ren | DOI:\n \n \n\n 10.1021/acs.est.3c00026 | https://pubmed.ncbi.nlm.nih.gov//37384597/ |
| 9 | Development of a machine learning-based acuity score prediction model for virtual care settings | Objective:\n \n \n Healthcare is increasingly digitized, yet remote and automated machine learning (ML) triage prediction systems for virtual urgent care use remain limited. The Canadian Triage and Acuity Scale (CTAS) is the gold... | 2023 Oct 3;23(1):200. | BMC Med Inform Decis Mak | Justin N Hall; Ron Galaev; Marina Gavrilov; Shawn Mondoux; Justin N Hall; Justin N Hall; Ron Galaev; Marina Gavrilov; Shawn Mondoux | DOI:\n \n \n\n 10.1186/s12911-023-02307-z | https://pubmed.ncbi.nlm.nih.gov//37789357/ |
| 10 | Blood Glucose Prediction Method Based on Particle Swarm Optimization and Model Fusion | Blood glucose stability in diabetic patients determines the degree of health, and changes in blood glucose levels are related to the outcome of diabetic patients. Therefore, accurate monitoring of blood glucose has a crucial role in controlling d... | 2022 Dec 6;12(12):3062. | Diagnostics (Basel) | He Xu; Shanjun Bao; Xiaoyu Zhang; Shangdong Liu; Wei Jing; Yimu Ji; He Xu; He Xu; Shanjun Bao; Xiaoyu Zhang; Shangdong Liu; Wei Jing; Yimu Ji | DOI:\n \n \n\n 10.3390/diagnostics12123062 | https://pubmed.ncbi.nlm.nih.gov//36553069/ |
| 11 | Machine learning for cell type classification from single nucleus RNA sequencing data | With the advent of single cell/nucleus RNA sequencing (sc/snRNA-seq), the field of cell phenotyping is now a data-driven exercise providing statistical evidence to support cell type/state categorization. However, the task of classifying cells int... | 2022 Sep 23;17(9):e0275070. | PLoS One | Huy Le; Beverly Peng; Janelle Uy; Daniel Carrillo; Yun Zhang; Brian D Aevermann; Richard H Scheuermann; Huy Le; Huy Le; Beverly Peng; Janelle Uy; Daniel Carrillo; Yun Zhang; Brian D Aevermann; Richard H Scheuermann | DOI:\n \n \n\n 10.1371/journal.pone.0275070 | https://pubmed.ncbi.nlm.nih.gov//36149937/ |
| 12 | Exploring Automated Machine Learning for Cognitive Outcome Prediction from Multimodal Brain Imaging using STREAMLINE | STREAMLINE is a simple, transparent, end-to-end automated machine learning (AutoML) pipeline for easily conducting rigorous machine learning (ML) modeling and analysis. The initial version is limited to binary classification. In this work, we ext... | 2023 Jun 16:2023:544-553. | AMIA Jt Summits Transl Sci Proc | Xinkai Wang; Yanbo Feng; Boning Tong; Jingxuan Bao; Marylyn D Ritchie; Andrew J Saykin; Jason H Moore; Ryan Urbanowicz; Li Shen; Xinkai Wang; Xinkai Wang; Yanbo Feng; Boning Tong; Jingxuan Bao; Marylyn D Ritchie; Andrew J Saykin; Jason H Moore; R... | None | https://pubmed.ncbi.nlm.nih.gov//37350896/ |
| 13 | Automated Machine Learning Pipeline Framework for Classification of Pediatric Functional Nausea Using High-Resolution Electrogastrogram | Objective:\n \n \n Pediatric functional nausea is challenging for patients to manage and for clinicians to treat since it lacks objective diagnosis and assessment. A data-driven non-invasive diagnostic screening tool that disting... | 2022 May;69(5):1717-1725. | IEEE Trans Biomed Eng | Joseph D Olson; Suseela Somarajan; Nicole D Muszynski; Andrew H Comstock; Kyra E Hendrickson; Lauren Scott; Alexandra Russell; Sari A Acra; Lynn Walker; Leonard A Bradshaw; Joseph D Olson; Joseph D Olson; Suseela Somarajan; Nicole D Muszynski; An... | DOI:\n \n \n\n 10.1109/TBME.2021.3129175 | https://pubmed.ncbi.nlm.nih.gov//34793297/ |
| 14 | Automated machine learning for early prediction of acute kidney injury in acute pancreatitis | Background:\n \n \n Acute kidney injury (AKI) represents a frequent and grave complication associated with acute pancreatitis (AP), substantially elevating both mortality rates and the financial burden of hospitalization. The aim... | 2024 Jan 11;24(1):16. | BMC Med Inform Decis Mak | Rufa Zhang; Minyue Yin; Anqi Jiang; Shihou Zhang; Xiaodan Xu; Luojie Liu; Rufa Zhang; Rufa Zhang; Minyue Yin; Anqi Jiang; Shihou Zhang; Xiaodan Xu; Luojie Liu | DOI:\n \n \n\n 10.1186/s12911-024-02414-5 | https://pubmed.ncbi.nlm.nih.gov//38212745/ |
| 15 | Super.Complex: A supervised machine learning pipeline for molecular complex detection in protein-interaction networks | Characterization of protein complexes, i.e. sets of proteins assembling into a single larger physical entity, is important, as such assemblies play many essential roles in cells such as gene regulation. From networks of protein-protein interactio... | 2021 Dec 31;16(12):e0262056. | PLoS One | Meghana Venkata Palukuri; Edward M Marcotte; Meghana Venkata Palukuri; Meghana Venkata Palukuri; Edward M Marcotte | DOI:\n \n \n\n 10.1371/journal.pone.0262056 | https://pubmed.ncbi.nlm.nih.gov//34972161/ |
| 16 | PromoterLCNN: A Light CNN-Based Promoter Prediction and Classification Model | Promoter identification is a fundamental step in understanding bacterial gene regulation mechanisms. However, accurate and fast classification of bacterial promoters continues to be challenging. New methods based on deep convolutional networks ha... | 2022 Jun 23;13(7):1126. | Genes (Basel) | Daryl Hernández; Nicolás Jara; Mauricio Araya; Roberto E Durán; Carlos Buil-Aranda; Daryl Hernández; Daryl Hernández; Nicolás Jara; Mauricio Araya; Roberto E Durán; Carlos Buil-Aranda | DOI:\n \n \n\n 10.3390/genes13071126 | https://pubmed.ncbi.nlm.nih.gov//35885909/ |
| 17 | Curated Database and Preliminary AutoML QSAR Model for 5-HT1A Receptor | Introduction of a new drug to the market is a challenging and resource-consuming process. Predictive models developed with the use of artificial intelligence could be the solution to the growing need for an efficient tool which brings practical a... | 2021 Oct 16;13(10):1711. | Pharmaceutics | Natalia Czub; Adam Pacławski; Jakub Szlęk; Aleksander Mendyk; Natalia Czub; Natalia Czub; Adam Pacławski; Jakub Szlęk; Aleksander Mendyk | DOI:\n \n \n\n 10.3390/pharmaceutics13101711 | https://pubmed.ncbi.nlm.nih.gov//34684004/ |
| 18 | Neural network hyperparameter optimization for prediction of real estate prices in Helsinki | Accurate price evaluation of real estate is beneficial for many parties involved in real estate business such as real estate companies, property owners, investors, banks, and financial institutes. Artificial Neural Networks (ANNs) have shown prom... | 2021 Apr 19:7:e444. | PeerJ Comput Sci | Jussi Kalliola; Jurgita Kapočiūtė-Dzikienė; Robertas Damaševičius; Jussi Kalliola; Jussi Kalliola; Jurgita Kapočiūtė-Dzikienė; Robertas Damaševičius | DOI:\n \n \n\n 10.7717/peerj-cs.444 | https://pubmed.ncbi.nlm.nih.gov//33977129/ |
| 19 | Automated Machine Learning to Develop Predictive Models of Metabolic Syndrome in Patients with Periodontal Disease | Metabolic syndrome is experiencing a concerning and escalating rise in prevalence today. The link between metabolic syndrome and periodontal disease is a highly relevant area of research. Some studies have suggested a bidirectional relationship b... | 2023 Dec 8;13(24):3631. | Diagnostics (Basel) | Ovidiu Boitor; Florin Stoica; Romeo Mihăilă; Laura Florentina Stoica; Laura Stef; Ovidiu Boitor; Ovidiu Boitor; Florin Stoica; Romeo Mihăilă; Laura Florentina Stoica; Laura Stef | DOI:\n \n \n\n 10.3390/diagnostics13243631 | https://pubmed.ncbi.nlm.nih.gov//38132215/ |
| 20 | Development of machine learning model for predicting prolonged operation time in lumbar stenosis undergoing posterior lumbar interbody fusion: a multicenter study | Background context:\n \n \n Longer posterior lumbar interbody fusion (PLIF) surgeries for individuals with lumbar spinal stenosis are linked to more complications and negatively affect recovery after the operation. Therefore, the... | 2025 Mar;25(3):460-473. | Spine J | Runmin Li; Lin Wang; Xinghao Wang; Marcin Grzegorzek; An-Tian Chen; Xubin Quan; Zhaohui Hu; Xiaozhu Liu; Yang Zhang; Tianyu Xiang; Yingang Zhang; Anfa Chen; Hao Jiang; Xuewen Hou; Qizhong Xu; Weiheng He; Liang Chen; Xin Zhou; Qiang Zhang; Wei Hua... | DOI:\n \n \n\n 10.1016/j.spinee.2024.10.001 | https://pubmed.ncbi.nlm.nih.gov//39427930/ |
| 21 | Accuracy of automated machine learning in classifying retinal pathologies from ultra-widefield pseudocolour fundus images | Aims:\n \n \n Automated machine learning (AutoML) is a novel tool in artificial intelligence (AI). This study assessed the discriminative performance of AutoML in differentiating retinal vein occlusion (RVO), retinitis pigmentosa... | 2023 Jan;107(1):90-95. | Br J Ophthalmol | Fares Antaki; Razek Georges Coussa; Ghofril Kahwati; Karim Hammamji; Mikael Sebag; Renaud Duval; Fares Antaki; Fares Antaki; Razek Georges Coussa; Ghofril Kahwati; Karim Hammamji; Mikael Sebag; Renaud Duval | DOI:\n \n \n\n 10.1136/bjophthalmol-2021-319030 | https://pubmed.ncbi.nlm.nih.gov//34344669/ |
| 22 | Diabetes mellitus prediction and diagnosis from a data preprocessing and machine learning perspective | Background and objective:\n \n \n Diabetes mellitus is a metabolic disorder characterized by hyperglycemia, which results from the inadequacy of the body to secrete and respond to insulin. If not properly managed or diagnosed on ... | 2022 Jun:220:106773. | Comput Methods Programs Biomed | Chollette C Olisah; Lyndon Smith; Melvyn Smith; Chollette C Olisah; Chollette C Olisah; Lyndon Smith; Melvyn Smith | DOI:\n \n \n\n 10.1016/j.cmpb.2022.106773 | https://pubmed.ncbi.nlm.nih.gov//35429810/ |
| 23 | Automated data preparation for in vivo tumor characterization with machine learning | Background:\n \n \n This study proposes machine learning-driven data preparation (MLDP) for optimal data preparation (DP) prior to building prediction models for cancer cohorts.\n \n\n\n Methods:\n \n \n ... | 2022 Oct 11:12:1017911. | Front Oncol | Denis Krajnc; Clemens P Spielvogel; Marko Grahovac; Boglarka Ecsedi; Sazan Rasul; Nina Poetsch; Tatjana Traub-Weidinger; Alexander R Haug; Zsombor Ritter; Hussain Alizadeh; Marcus Hacker; Thomas Beyer; Laszlo Papp; Denis Krajnc; Denis Krajnc; Cle... | DOI:\n \n \n\n 10.3389/fonc.2022.1017911 | https://pubmed.ncbi.nlm.nih.gov//36303841/ |
| 24 | Automated Multimodal Machine Learning for Esophageal Variceal Bleeding Prediction Based on Endoscopy and Structured Data | Esophageal variceal (EV) bleeding is a severe medical emergency related to cirrhosis. Early identification of cirrhotic patients with at a high risk of EV bleeding is key to improving outcomes and optimizing medical resources. This study aimed to... | 2023 Feb;36(1):326-338. | J Digit Imaging | Yu Wang; Yu Hong; Yue Wang; Xin Zhou; Xin Gao; Chenyan Yu; Jiaxi Lin; Lu Liu; Jingwen Gao; Minyue Yin; Guoting Xu; Xiaolin Liu; Jinzhou Zhu; Yu Wang; Yu Wang; Yu Hong; Yue Wang; Xin Zhou; Xin Gao; Chenyan Yu; Jiaxi Lin; Lu Liu; Jingwen Gao; Minyu... | DOI:\n \n \n\n 10.1007/s10278-022-00724-6 | https://pubmed.ncbi.nlm.nih.gov//36279027/ |
| 25 | Sample size and predictive performance of machine learning methods with survival data: A simulation study | Prediction models are increasingly developed and used in diagnostic and prognostic studies, where the use of machine learning (ML) methods is becoming more and more popular over traditional regression techniques. For survival outcomes the Cox pro... | 2023 Dec 30;42(30):5657-5675. | Stat Med | Gabriele Infante; Rosalba Miceli; Federico Ambrogi; Gabriele Infante; Gabriele Infante; Rosalba Miceli; Federico Ambrogi | DOI:\n \n \n\n 10.1002/sim.9931 | https://pubmed.ncbi.nlm.nih.gov//37947168/ |
| 26 | Automated Machine Learning Model Development for Intracranial Aneurysm Treatment Outcome Prediction: A Feasibility Study | Background: The prediction of aneurysm treatment outcomes can help to optimize the treatment strategies. Machine learning (ML) has shown positive results in many clinical areas. However, the development of such models requires expertise in ML, wh... | 2021 Nov 29:12:735142. | Front Neurol | Chubin Ou; Jiahui Liu; Yi Qian; Winston Chong; Dangqi Liu; Xuying He; Xin Zhang; Chuan-Zhi Duan; Chubin Ou; Chubin Ou; Jiahui Liu; Yi Qian; Winston Chong; Dangqi Liu; Xuying He; Xin Zhang; Chuan-Zhi Duan | DOI:\n \n \n\n 10.3389/fneur.2021.735142 | https://pubmed.ncbi.nlm.nih.gov//34912282/ |
| 27 | Metaheuristic optimization of data preparation and machine learning hyperparameters for prediction of dynamic methane production | Machine learning algorithms provide detailed description of the anaerobic digestion process, but the impact of data preparation procedures and hyperparameter optimization has rarely been investigated. A genetic algorithm was developed for optimiz... | 2023 Mar:372:128604. | Bioresour Technol | Alberto Meola; Manuel Winkler; Sören Weinrich; Alberto Meola; Alberto Meola; Manuel Winkler; Sören Weinrich | DOI:\n \n \n\n 10.1016/j.biortech.2023.128604 | https://pubmed.ncbi.nlm.nih.gov//36634878/ |
| 28 | Machine learning in health care and laboratory medicine: General overview of supervised learning and Auto-ML | Artificial Intelligence (AI) and machine learning (ML) have now spawned a new field within health care and health science research. These new predictive analytics tools are starting to change various facets of our clinical care domains including ... | 2021 Jul:43 Suppl 1:15-22. | Int J Lab Hematol | Hooman H Rashidi; Nam Tran; Samer Albahra; Luke T Dang; Hooman H Rashidi; Hooman H Rashidi; Nam Tran; Samer Albahra; Luke T Dang | DOI:\n \n \n\n 10.1111/ijlh.13537 | https://pubmed.ncbi.nlm.nih.gov//34288435/ |
| 29 | Biomedical image classification made easier thanks to transfer and semi-supervised learning | Background and objectives:\n \n \n Deep learning techniques are the state-of-the-art approach to solve image classification problems in biomedicine; however, they require the acquisition and annotation of a considerable volume of... | 2021 Jan:198:105782. | Comput Methods Programs Biomed | A Inés; C Domínguez; J Heras; E Mata; V Pascual; A Inés; A Inés; C Domínguez; J Heras; E Mata; V Pascual | DOI:\n \n \n\n 10.1016/j.cmpb.2020.105782 | https://pubmed.ncbi.nlm.nih.gov//33065493/ |
| 30 | Automated machine learning in nanotoxicity assessment: A comparative study of predictive model performance | Computational modeling has earned significant interest as an alternative to animal testing of toxicity assessment. However, the process of selecting an appropriate algorithm and fine-tuning hyperparameters for the developing of optimized models t... | 2024 Feb 9:25:9-19. | Comput Struct Biotechnol J | Xiao Xiao; Tung X Trinh; Zayakhuu Gerelkhuu; Eunyong Ha; Tae Hyun Yoon; Xiao Xiao; Xiao Xiao; Tung X Trinh; Zayakhuu Gerelkhuu; Eunyong Ha; Tae Hyun Yoon | DOI:\n \n \n\n 10.1016/j.csbj.2024.02.003 | https://pubmed.ncbi.nlm.nih.gov//38414794/ |
| 31 | Improving sepsis classification performance with artificial intelligence algorithms: A comprehensive overview of healthcare applications | Purpose:\n \n \n This study investigates the potential of machine learning (ML) algorithms in improving sepsis diagnosis and prediction, focusing on their relevance in healthcare decision-making. The primary objective is to contr... | 2024 Oct:83:154815. | J Crit Care | Anjana G; Nisha K L; Arun Sankar M S; Anjana G; Anjana G; Nisha K L; Arun Sankar M S | DOI:\n \n \n\n 10.1016/j.jcrc.2024.154815 | https://pubmed.ncbi.nlm.nih.gov//38723336/ |
| 32 | Systematic mapping of global research on climate and health: a machine learning review | Background:\n \n \n The global literature on the links between climate change and human health is large, increasing exponentially, and it is no longer feasible to collate and synthesise using traditional systematic evidence mappi... | 2021 Aug;5(8):e514-e525. | Lancet Planet Health | Lea Berrang-Ford; Anne J Sietsma; Max Callaghan; Jan C Minx; Pauline F D Scheelbeek; Neal R Haddaway; Andy Haines; Alan D Dangour; Lea Berrang-Ford; Lea Berrang-Ford; Anne J Sietsma; Max Callaghan; Jan C Minx; Pauline F D Scheelbeek; Neal R Hadda... | DOI:\n \n \n\n 10.1016/S2542-5196(21)00179-0 | https://pubmed.ncbi.nlm.nih.gov//34270917/ |
| 33 | Machine Learning-Based Automated Detection of Hydroxychloroquine Toxicity and Prediction of Future Toxicity Using Higher-Order OCT Biomarkers | Objective:\n \n \n Despite guidelines for hydroxychloroquine (HCQ) toxicity screening, there are clear challenges to accurate detection and interpretation. In the current report, the feasibility of automated machine learning (ML)... | 2022 Dec;6(12):1241-1252. | Ophthalmol Retina | Gagan Kalra; Katherine E Talcott; Stephanie Kaiser; Obinna Ugwuegbu; Ming Hu; Sunil K Srivastava; Justis P Ehlers; Gagan Kalra; Gagan Kalra; Katherine E Talcott; Stephanie Kaiser; Obinna Ugwuegbu; Ming Hu; Sunil K Srivastava; Justis P Ehlers | DOI:\n \n \n\n 10.1016/j.oret.2022.05.031 | https://pubmed.ncbi.nlm.nih.gov//35691579/ |
| 34 | Data processing pipeline for cardiogenic shock prediction using machine learning | Introduction:\n \n \n Recent advances in machine learning provide new possibilities to process and analyse observational patient data to predict patient outcomes. In this paper, we introduce a data processing pipeline for cardiog... | 2023 Mar 23:10:1132680. | Front Cardiovasc Med | Nikola Jajcay; Branislav Bezak; Amitai Segev; Shlomi Matetzky; Jana Jankova; Michael Spartalis; Mohammad El Tahlawi; Federico Guerra; Julian Friebel; Tharusan Thevathasan; Imrich Berta; Leo Pölzl; Felix Nägele; Edita Pogran; F Aaysha Cader; Milan... | DOI:\n \n \n\n 10.3389/fcvm.2023.1132680 | https://pubmed.ncbi.nlm.nih.gov//37034352/ |
| 35 | Establishment and Validation of a Machine Learning-Based Prediction Model for Termination of Pregnancy via Cesarean Section | Objective:\n \n \n This study aimed to investigate the risk factors of cesarean section and establish a prediction model for cesarean section based on the characteristics of pregnant women.\n \n\n\n Methods:\n ... | 2023 Nov 24:16:5567-5578. | Int J Gen Med | Rui Zhang; Weixuan Sheng; Feiran Liu; Jin Zhang; Wenpei Bai; Rui Zhang; Rui Zhang; Weixuan Sheng; Feiran Liu; Jin Zhang; Wenpei Bai | DOI:\n \n \n\n 10.2147/IJGM.S413736 | https://pubmed.ncbi.nlm.nih.gov//38034896/ |
| 36 | Accelerating Surgical Site Infection Abstraction With a Semi-automated Machine-learning Approach | Objective:\n \n \n To demonstrate that a semi-automated approach to health data abstraction provides significant efficiencies and high accuracy.\n \n\n\n Background:\n \n \n Surgical outcome abstracti... | 2022 Jul 1;276(1):180-185. | Ann Surg | Steven J Skube; Zhen Hu; Gyorgy J Simon; Elizabeth C Wick; Elliot G Arsoniadis; Clifford Y Ko; Genevieve B Melton; Steven J Skube; Steven J Skube; Zhen Hu; Gyorgy J Simon; Elizabeth C Wick; Elliot G Arsoniadis; Clifford Y Ko; Genevieve B Melton | DOI:\n \n \n\n 10.1097/SLA.0000000000004354 | https://pubmed.ncbi.nlm.nih.gov//33074897/ |
| 37 | Self-Supervised Node Classification with Strategy and Actively Selected Labeled Set | To alleviate the impact of insufficient labels in less-labeled classification problems, self-supervised learning improves the performance of graph neural networks (GNNs) by focusing on the information of unlabeled nodes. However, none of the exis... | 2022 Dec 23;25(1):30. | Entropy (Basel) | Yi Kang; Ke Liu; Zhiyuan Cao; Jiacai Zhang; Yi Kang; Yi Kang; Ke Liu; Zhiyuan Cao; Jiacai Zhang | DOI:\n \n \n\n 10.3390/e25010030 | https://pubmed.ncbi.nlm.nih.gov//36673172/ |
| 38 | Research on Hyperparameter Optimization of Concrete Slump Prediction Model Based on Response Surface Method | In this paper, eight variables of cement, blast furnace slag, fly ash, water, superplasticizer, coarse aggregate, fine aggregate and flow are used as network input and slump is used as network output to construct a back-propagation (BP) neural ne... | 2022 Jul 5;15(13):4721. | Materials (Basel) | Yuan Chen; Jiaye Wu; Yingqian Zhang; Lei Fu; Yunrong Luo; Yong Liu; Lindan Li; Yuan Chen; Yuan Chen; Jiaye Wu; Yingqian Zhang; Lei Fu; Yunrong Luo; Yong Liu; Lindan Li | DOI:\n \n \n\n 10.3390/ma15134721 | https://pubmed.ncbi.nlm.nih.gov//35806843/ |
| 39 | Instance-Level Microtubule Tracking | We propose a new method of instance-level microtubule (MT) tracking in time-lapse image series using recurrent attention. Our novel deep learning algorithm segments individual MTs at each frame. Segmentation results from successive frames are use... | 2020 Jun;39(6):2061-2075. | IEEE Trans Med Imaging | Samira Masoudi; Afsaneh Razi; Cameron H G Wright; Jesse C Gatlin; Ulas Bagci; Samira Masoudi; Samira Masoudi; Afsaneh Razi; Cameron H G Wright; Jesse C Gatlin; Ulas Bagci | DOI:\n \n \n\n 10.1109/TMI.2019.2963865 | https://pubmed.ncbi.nlm.nih.gov//31905134/ |
| 40 | Concrete Strength Prediction Using Different Machine Learning Processes: Effect of Slag, Fly Ash and Superplasticizer | Blast furnace slag (BFS) and fly ash (FA), as mining-associated solid wastes with good pozzolanic effects, can be combined with superplasticizer to prepare concrete with less cement utilization. Considering the important influence of strength on ... | 2022 Aug 4;15(15):5369. | Materials (Basel) | Chongchong Qi; Binhan Huang; Mengting Wu; Kun Wang; Shan Yang; Guichen Li; Chongchong Qi; Chongchong Qi; Binhan Huang; Mengting Wu; Kun Wang; Shan Yang; Guichen Li | DOI:\n \n \n\n 10.3390/ma15155369 | https://pubmed.ncbi.nlm.nih.gov//35955301/ |
| 41 | Prediction of illness remission in patients with Obsessive-Compulsive Disorder with supervised machine learning | Introduction:\n \n \n The course of OCD differs widely among OCD patients, varying from chronic symptoms to full remission. No tools for individual prediction of OCD remission are currently available. This study aimed to develop ... | 2022 Jan 1:296:117-125. | J Affect Disord | Massimiliano Grassi; Judith Rickelt; Daniela Caldirola; Merijn Eikelenboom; Patricia van Oppen; Michel Dumontier; Giampaolo Perna; Koen Schruers; Massimiliano Grassi; Massimiliano Grassi; Judith Rickelt; Daniela Caldirola; Merijn Eikelenboom; Pat... | DOI:\n \n \n\n 10.1016/j.jad.2021.09.042 | https://pubmed.ncbi.nlm.nih.gov//34600172/ |
| 42 | Enhanced Gravitational Search Optimization with Hybrid Deep Learning Model for COVID-19 Diagnosis on Epidemiology Data | Effective screening provides efficient and quick diagnoses of COVID-19 and could alleviate related problems in the health care system. A prediction model that combines multiple features to assess contamination risks was established in the hope of... | 2022 Jul 19;10(7):1339. | Healthcare (Basel) | Mahmoud Ragab; Hani Choudhry; Amer H Asseri; Sami Saeed Binyamin; Mohammed W Al-Rabia; Mahmoud Ragab; Mahmoud Ragab; Hani Choudhry; Amer H Asseri; Sami Saeed Binyamin; Mohammed W Al-Rabia | DOI:\n \n \n\n 10.3390/healthcare10071339 | https://pubmed.ncbi.nlm.nih.gov//35885865/ |
| 43 | Enhancing diabetic foot ulcer prediction with machine learning: A focus on Localized examinations | Background:\n \n \n diabetices foot ulcer (DFU) are serious complications. It is crucial to detect and diagnose DFU early in order to provide timely treatment, improve patient quality of life, and avoid the social and economic co... | 2024 Sep 19;10(19):e37635. | Heliyon | Wang Xiaoling; Zhu Shengmei; Wang BingQian; Li Wen; Gu Shuyan; Chen Hanbei; Qin Chenjie; Dai Yao; Li Jutang; Wang Xiaoling; Wang Xiaoling; Zhu Shengmei; Wang BingQian; Li Wen; Gu Shuyan; Chen Hanbei; Qin Chenjie; Dai Yao; Li Jutang | DOI:\n \n \n\n 10.1016/j.heliyon.2024.e37635 | https://pubmed.ncbi.nlm.nih.gov//39386877/ |
| 44 | The Prediction of Recombination Hotspot Based on Automated Machine Learning | Meiotic recombination plays a pivotal role in genetic evolution. Genetic variation induced by recombination is a crucial factor in generating biodiversity and a driving force for evolution. At present, the development of recombination hotspot pre... | 2025 Mar 15;437(6):168653. | J Mol Biol | Dong-Xin Ye; Jun-Wen Yu; Rui Li; Yu-Duo Hao; Tian-Yu Wang; Hui Yang; Hui Ding; Dong-Xin Ye; Dong-Xin Ye; Jun-Wen Yu; Rui Li; Yu-Duo Hao; Tian-Yu Wang; Hui Yang; Hui Ding | DOI:\n \n \n\n 10.1016/j.jmb.2024.168653 | https://pubmed.ncbi.nlm.nih.gov//38871176/ |
| 45 | Super.Complex: A supervised machine learning pipeline for molecular complex detection in protein-interaction networks | Characterization of protein complexes, i.e. sets of proteins assembling into a single larger physical entity, is important, as such assemblies play many essential roles in cells such as gene regulation. From networks of protein-protein interactio... | 2021 Oct 11:2021.06.22.449395. | bioRxiv | Meghana V Palukuri; Edward M Marcotte; Meghana V Palukuri; Meghana V Palukuri; Edward M Marcotte | DOI:\n \n \n\n 10.1101/2021.06.22.449395 | https://pubmed.ncbi.nlm.nih.gov//34189530/ |
| 46 | Evaluating an automated machine learning model that predicts visual acuity outcomes in patients with neovascular age-related macular degeneration | Purpose:\n \n \n Neovascular age-related macular degeneration (nAMD) is a major global cause of blindness. Whilst anti-vascular endothelial growth factor (anti-VEGF) treatment is effective, response varies considerably between in... | 2022 Aug;260(8):2461-2473. | Graefes Arch Clin Exp Ophthalmol | Abdallah Abbas; Ciara O'Byrne; Dun Jack Fu; Gabriella Moraes; Konstantinos Balaskas; Robbert Struyven; Sara Beqiri; Siegfried K Wagner; Edward Korot; Pearse A Keane; Abdallah Abbas; Abdallah Abbas; Ciara O'Byrne; Dun Jack Fu; Gabriella Moraes; Ko... | DOI:\n \n \n\n 10.1007/s00417-021-05544-y | https://pubmed.ncbi.nlm.nih.gov//35122132/ |
| 47 | Enhancing machine learning performance in cardiac surgery ICU: Hyperparameter optimization with metaheuristic algorithm | The healthcare industry is generating a massive volume of data, promising a potential goldmine of information that can be extracted through machine learning (ML) techniques. The Intensive Care Unit (ICU) stands out as a focal point within hospita... | 2025 Feb 10;20(2):e0311250. | PLoS One | Ali Bahrami; Morteza Rakhshaninejad; Rouzbeh Ghousi; Alireza Atashi; Ali Bahrami; Ali Bahrami; Morteza Rakhshaninejad; Rouzbeh Ghousi; Alireza Atashi | DOI:\n \n \n\n 10.1371/journal.pone.0311250 | https://pubmed.ncbi.nlm.nih.gov//39928609/ |
| 48 | AutoML for Multi-Label Classification: Overview and Empirical Evaluation | Automated machine learning (AutoML) supports the algorithmic construction and data-specific customization of machine learning pipelines, including the selection, combination, and parametrization of machine learning algorithms as main constituents... | 2021 Sep;43(9):3037-3054. | IEEE Trans Pattern Anal Mach Intell | Marcel Wever; Alexander Tornede; Felix Mohr; Eyke Hullermeier; Marcel Wever; Marcel Wever; Alexander Tornede; Felix Mohr; Eyke Hullermeier | DOI:\n \n \n\n 10.1109/TPAMI.2021.3051276 | https://pubmed.ncbi.nlm.nih.gov//33439834/ |
| 49 | Analyzing Perceived Psychological and Social Stress of University Students: A Machine Learning Approach | The COVID-19 pandemic has worsened the psychological and social stress levels of university students due to physical illness, enhanced dependence on mobile devices and internet, a lack of social activities, and home confinement. Therefore, early ... | 2023 Jun;9(6):e17307. | Heliyon | Ishrak Jahan Ratul; Mirza Muntasir Nishat; Fahim Faisal; Sadia Sultana; Ashik Ahmed; Md Abdullah Al Mamun; Ishrak Jahan Ratul; Ishrak Jahan Ratul; Mirza Muntasir Nishat; Fahim Faisal; Sadia Sultana; Ashik Ahmed; Md Abdullah Al Mamun | DOI:\n \n \n\n 10.1016/j.heliyon.2023.e17307 | https://pubmed.ncbi.nlm.nih.gov//37332920/ |
| 50 | Differentiation between Descending Thoracic Aortic Diseases using Machine Learning and Plasma Proteomic Signatures | Background:\n \n \n Descending thoracic aortic aneurysms and dissections can go undetected until severe and catastrophic, and few clinical indices exist to screen for aneurysms or predict risk of dissection.\n \n\n\n ... | 2023 Oct 23:2023.04.26.538468. | bioRxiv | Amanda Momenzadeh; Simion Kreimer; Dongchuan Guo; Matthew Ayres; Daniel Berman; Kuang-Yuh Chyu; Prediman K Shah; Dianna Milewicz; Ali Azizzadeh; Jesse G Meyer; Sarah Parker; Amanda Momenzadeh; Amanda Momenzadeh; Simion Kreimer; Dongchuan Guo; Mat... | DOI:\n \n \n\n 10.1101/2023.04.26.538468 | https://pubmed.ncbi.nlm.nih.gov//37162892/ |
| 51 | Automated machine learning-based classification of proliferative and non-proliferative diabetic retinopathy using optical coherence tomography angiography vascular density maps | Purpose:\n \n \n The study aims to classify the eyes with proliferative diabetic retinopathy (PDR) and non-proliferative diabetic retinopathy (NPDR) based on the optical coherence tomography angiography (OCTA) vascular density ma... | 2023 Feb;261(2):391-399. | Graefes Arch Clin Exp Ophthalmol | Elias Khalili Pour; Khosro Rezaee; Hossein Azimi; Seyed Mohammad Mirshahvalad; Behzad Jafari; Kaveh Fadakar; Hooshang Faghihi; Ahmad Mirshahi; Fariba Ghassemi; Nazanin Ebrahimiadib; Masoud Mirghorbani; Fatemeh Bazvand; Hamid Riazi-Esfahani; Moham... | DOI:\n \n \n\n 10.1007/s00417-022-05818-z | https://pubmed.ncbi.nlm.nih.gov//36050474/ |
| 52 | Identification of testicular cancer with T2-weighted MRI-based radiomics and automatic machine learning | Background:\n \n \n Distinguishing between benign and malignant testicular lesions on clinical magnetic resonance imaging (MRI) is crucial for guiding treatment planning. However, conventional MRI-based radiomics to identify test... | 2025 Mar 28;25(1):563. | BMC Cancer | Liang Wang; PeiPei Zhang; Yanhui Feng; Wenzhi Lv; Xiangde Min; Zhiyong Liu; Jin Li; Zhaoyan Feng; Liang Wang; Liang Wang; PeiPei Zhang; Yanhui Feng; Wenzhi Lv; Xiangde Min; Zhiyong Liu; Jin Li; Zhaoyan Feng | DOI:\n \n \n\n 10.1186/s12885-025-13844-3 | https://pubmed.ncbi.nlm.nih.gov//40155850/ |
| 53 | Weakly-supervised learning-based pathology detection and localization in 3D chest CT scans | Background:\n \n \n Recent advancements in anomaly detection have paved the way for novel radiological reading assistance tools that support the identification of findings, aimed at saving time. The clinical adoption of such appl... | 2024 Nov;51(11):8272-8282. | Med Phys | Aissam Djahnine; Emilien Jupin-Delevaux; Olivier Nempont; Salim Aymeric Si-Mohamed; Fabien Craighero; Vincent Cottin; Philippe Douek; Alexandre Popoff; Loic Boussel; Aissam Djahnine; Aissam Djahnine; Emilien Jupin-Delevaux; Olivier Nempont; Salim... | DOI:\n \n \n\n 10.1002/mp.17302 | https://pubmed.ncbi.nlm.nih.gov//39140793/ |
| 54 | Explainable coronary artery disease prediction model based on AutoGluon from AutoML framework | Objective:\n \n \n This study focuses on the innovative application of Automated Machine Learning (AutoML) technology in cardiovascular medicine to construct an explainable Coronary Artery Disease (CAD) prediction model to suppor... | 2024 Jul 1:11:1360548. | Front Cardiovasc Med | Jianghong Wang; Qiang Xue; Chris W J Zhang; Kelvin Kian Loong Wong; Zhihua Liu; Jianghong Wang; Jianghong Wang; Qiang Xue; Chris W J Zhang; Kelvin Kian Loong Wong; Zhihua Liu | DOI:\n \n \n\n 10.3389/fcvm.2024.1360548 | https://pubmed.ncbi.nlm.nih.gov//39011494/ |
| 55 | Predictive Analysis of Diabetes-Risk with Class Imbalance | Diabetes type 2 (T2DM) is a common chronic disease, increasingly leading to many complications and affecting vital organs. Hyperglycemia is the main characteristic caused by insufficient insulin secretion and poses a serious risk to human health.... | 2022 Oct 11:2022:3078025. | Comput Intell Neurosci | Ahmed I ElSeddawy; Faten Khalid Karim; Aisha Mohamed Hussein; Doaa Sami Khafaga; Ahmed I ElSeddawy; Ahmed I ElSeddawy; Faten Khalid Karim; Aisha Mohamed Hussein; Doaa Sami Khafaga | DOI:\n \n \n\n 10.1155/2022/3078025 | https://pubmed.ncbi.nlm.nih.gov//36268149/ |
| 56 | Using artificial intelligence to overcome over-indebtedness and fight poverty | This research examines how artificial intelligence may contribute to better understanding and to overcome over-indebtedness in contexts of high poverty risk. This research uses Automated Machine Learning (AutoML) in a field database of 1654 over-... | 2021 Jul:131:411-425. | J Bus Res | Mário Boto Ferreira; Diego Costa Pinto; Márcia Maurer Herter; Jerônimo Soro; Leonardo Vanneschi; Mauro Castelli; Fernando Peres; Mário Boto Ferreira; Mário Boto Ferreira; Diego Costa Pinto; Márcia Maurer Herter; Jerônimo Soro; Leonardo Vanneschi;... | DOI:\n \n \n\n 10.1016/j.jbusres.2020.10.035 | https://pubmed.ncbi.nlm.nih.gov//33100428/ |
| 57 | Construction and evaluation of prediction model for postoperative re-fractures in elderly patients with hip fractures | Objective:\n \n \n The aim of study was to construct a postoperative re-fracture prediction model for elderly hip fracture patients using an automated machine learning algorithm to provide a basis for early identification of pati... | 2025 Mar:195:105738. | Int J Med Inform | Jingjing Wu; Qingqing Zeng; Sijie Gui; Zhuolan Li; Wanyu Miao; Mi Zeng; Manyi Wang; Li Hu; Guqing Zeng; Jingjing Wu; Jingjing Wu; Qingqing Zeng; Sijie Gui; Zhuolan Li; Wanyu Miao; Mi Zeng; Manyi Wang; Li Hu; Guqing Zeng | DOI:\n \n \n\n 10.1016/j.ijmedinf.2024.105738 | https://pubmed.ncbi.nlm.nih.gov//39644793/ |
| 58 | Machine learning in obsessive-compulsive disorder medications | Obsessive-compulsive disorder (OCD) is the fourth most common psychiatric disorder with a significant morbidity rate. Despite various treatment modalities and medications, some patients show no definitive response. The aim of this study is to cla... | 2024 Nov 5;10(21):e40136. | Heliyon | Mahdiyeh Khazaneha; Behnaz Bakhshinejad; Mitra Mehrabani; Abdolreza Sabahi; Mohammad Khaksari; Mehdi Shafiee; Mohsen Nakhaie; Mohammad Rezaei Zadeh Rukerd; Abdollah Jafarzadeh; Mehrzad Mehrbani; Mahdiyeh Khazaneha; Mahdiyeh Khazaneha; Behnaz Bakh... | DOI:\n \n \n\n 10.1016/j.heliyon.2024.e40136 | https://pubmed.ncbi.nlm.nih.gov//39583807/ |
| 59 | COVID-19 Risk Prediction for Diabetic Patients Using Fuzzy Inference System and Machine Learning Approaches | Individuals with pre-existing diabetes seem to be vulnerable to the COVID-19 due to changes in blood sugar levels and diabetes complications. As observed globally, around 20-50% of individuals affected by coronavirus had diabetes. However, there ... | 2022 Apr 1:2022:4096950. | J Healthc Eng | Alok Aggarwal; Madam Chakradar; Manpreet Singh Bhatia; Manoj Kumar; Thompson Stephan; Sachin Kumar Gupta; S H Alsamhi; Hatem Al-Dois; Alok Aggarwal; Alok Aggarwal; Madam Chakradar; Manpreet Singh Bhatia; Manoj Kumar; Thompson Stephan; Sachin Kuma... | DOI:\n \n \n\n 10.1155/2022/4096950 | https://pubmed.ncbi.nlm.nih.gov//35368915/ |
| 60 | Prediction of body condition in Jersey dairy cattle from 3D-images using machine learning techniques | The body condition of dairy cows is a crucial health and welfare indicator that is widely acknowledged. Dairy herds with a well-management body condition tend to have more fertile and functional cows. Therefore, routine recording of high-quality ... | 2023 Jan 3:101:skad376. | J Anim Sci | Rasmus B Stephansen; Coralia I V Manzanilla-Pech; Grum Gebreyesus; Goutam Sahana; Jan Lassen; Rasmus B Stephansen; Rasmus B Stephansen; Coralia I V Manzanilla-Pech; Grum Gebreyesus; Goutam Sahana; Jan Lassen | DOI:\n \n \n\n 10.1093/jas/skad376 | https://pubmed.ncbi.nlm.nih.gov//37943499/ |
| 61 | The variant artificial intelligence easy scoring (VARIES) system | Purpose:\n \n \n Medical artificial intelligence (MAI) is artificial intelligence (AI) applied to the healthcare field. AI can be applied to many different aspects of genetics, such as variant classification. With little or no pr... | 2022 Jun:145:105492. | Comput Biol Med | Taghrid Aloraini; Abdulrhman Aljouie; Rashed Alniwaider; Wardah Alharbi; Lamia Alsubaie; Wafaa AlTuraif; Waseem Qureshi; Abdulrahman Alswaid; Wafaa Eyiad; Fuad Al Mutairi; Faroug Ababneh; Majid Alfadhel; Ahmed Alfares; Taghrid Aloraini; Taghrid A... | DOI:\n \n \n\n 10.1016/j.compbiomed.2022.105492 | https://pubmed.ncbi.nlm.nih.gov//35585733/ |
| 62 | HGSORF: Henry Gas Solubility Optimization-based Random Forest for C-Section prediction and XAI-based cause analysis | A stable predictive model is essential for forecasting the chances of cesarean or C-section (CS) delivery, as unnecessary CS delivery can adversely affect neonatal, maternal, and pediatric morbidity and mortality, and can incur significant financ... | 2022 Aug:147:105671. | Comput Biol Med | Md Saiful Islam; Md Abdul Awal; Jinnaton Nessa Laboni; Farhana Tazmim Pinki; Shatu Karmokar; Khondoker Mirazul Mumenin; Saad Al-Ahmadi; Md Ashfikur Rahman; Md Shahadat Hossain; Seyedali Mirjalili; Md Saiful Islam; Md Saiful Islam; Md Abdul Awal; ... | DOI:\n \n \n\n 10.1016/j.compbiomed.2022.105671 | https://pubmed.ncbi.nlm.nih.gov//35660327/ |
| 63 | Semi-supervised learning for somatic variant calling and peptide identification in personalized cancer immunotherapy | Background:\n \n \n Personalized cancer vaccines are emerging as one of the most promising approaches to immunotherapy of advanced cancers. However, only a small proportion of the neoepitopes generated by somatic DNA mutations in... | 2020 Dec 30;21(Suppl 18):498. | BMC Bioinformatics | Elham Sherafat; Jordan Force; Ion I Măndoiu; Elham Sherafat; Elham Sherafat; Jordan Force; Ion I Măndoiu | DOI:\n \n \n\n 10.1186/s12859-020-03813-x | https://pubmed.ncbi.nlm.nih.gov//33375939/ |
| 64 | Scaling tree-based automated machine learning to biomedical big data with a feature set selector | Motivation:\n \n \n Automated machine learning (AutoML) systems are helpful data science assistants designed to scan data for novel features, select appropriate supervised learning models and optimize their parameters. For this p... | 2020 Jan 1;36(1):250-256. | Bioinformatics | Trang T Le; Weixuan Fu; Jason H Moore; Trang T Le; Trang T Le; Weixuan Fu; Jason H Moore | DOI:\n \n \n\n 10.1093/bioinformatics/btz470 | https://pubmed.ncbi.nlm.nih.gov//31165141/ |
| 65 | Ultrasound-Based Machine Learning Approach for Detection of Nonalcoholic Fatty Liver Disease | Objectives:\n \n \n Current diagnosis of nonalcoholic fatty liver disease (NAFLD) relies on biopsy or MR-based fat quantification. This prospective study explored the use of ultrasound with artificial intelligence for the detecti... | 2023 Aug;42(8):1747-1756. | J Ultrasound Med | Aylin Tahmasebi; Shuo Wang; Corinne E Wessner; Trang Vu; Ji-Bin Liu; Flemming Forsberg; Jesse Civan; Flavius F Guglielmo; John R Eisenbrey; Aylin Tahmasebi; Aylin Tahmasebi; Shuo Wang; Corinne E Wessner; Trang Vu; Ji-Bin Liu; Flemming Forsberg; J... | DOI:\n \n \n\n 10.1002/jum.16194 | https://pubmed.ncbi.nlm.nih.gov//36807314/ |
| 66 | Using Automated Machine Learning to Predict the Mortality of Patients With COVID-19: Prediction Model Development Study | Background:\n \n \n During a pandemic, it is important for clinicians to stratify patients and decide who receives limited medical resources. Machine learning models have been proposed to accurately predict COVID-19 disease sever... | 2021 Feb 26;23(2):e23458. | J Med Internet Res | Kenji Ikemura; Eran Bellin; Yukako Yagi; Henny Billett; Mahmoud Saada; Katelyn Simone; Lindsay Stahl; James Szymanski; D Y Goldstein; Morayma Reyes Gil; Kenji Ikemura; Kenji Ikemura; Eran Bellin; Yukako Yagi; Henny Billett; Mahmoud Saada; Katelyn... | DOI:\n \n \n\n 10.2196/23458 | https://pubmed.ncbi.nlm.nih.gov//33539308/ |
| 67 | A User-friendly Approach for the Diagnosis of Diabetic Retinopathy Using ChatGPT and Automated Machine Learning | Purpose:\n \n \n To assess the capabilities of Chat Generative Pre-trained Transformer (ChatGPT) and Vertex AI in executing code-free preprocessing, training machine learning (ML) models, and analyzing the data.\n \n\n\n ... | 2024 Feb 21;4(4):100495. | Ophthalmol Sci | S Saeed Mohammadi; Quan Dong Nguyen; S Saeed Mohammadi; S Saeed Mohammadi; Quan Dong Nguyen | DOI:\n \n \n\n 10.1016/j.xops.2024.100495 | https://pubmed.ncbi.nlm.nih.gov//38690313/ |
| 68 | Combination of whole genome sequencing and supervised machine learning provides unambiguous identification of eae-positive Shiga toxin-producing Escherichia coli | Introduction:\n \n \n The objective of this study was to develop, using a genome wide machine learning approach, an unambiguous model to predict the presence of highly pathogenic STEC in E. coli reads assemblies derived from comp... | 2023 May 12:14:1118158. | Front Microbiol | Fabien Vorimore; Sandra Jaudou; Mai-Lan Tran; Hugues Richard; Patrick Fach; Sabine Delannoy; Fabien Vorimore; Fabien Vorimore; Sandra Jaudou; Mai-Lan Tran; Hugues Richard; Patrick Fach; Sabine Delannoy | DOI:\n \n \n\n 10.3389/fmicb.2023.1118158 | https://pubmed.ncbi.nlm.nih.gov//37250024/ |
| 69 | Differentiation between descending thoracic aortic diseases using machine learning and plasma proteomic signatures | Background:\n \n \n Descending thoracic aortic aneurysms and dissections can go undetected until severe and catastrophic, and few clinical indices exist to screen for aneurysms or predict risk of dissection.\n \n\n\n ... | 2024 Jun 2;21(1):38. | Clin Proteomics | Amanda Momenzadeh; Simion Kreimer; Dongchuan Guo; Matthew Ayres; Daniel Berman; Kuang-Yuh Chyu; Prediman K Shah; Dianna Milewicz; Ali Azizzadeh; Jesse G Meyer; Sarah Parker; Amanda Momenzadeh; Amanda Momenzadeh; Simion Kreimer; Dongchuan Guo; Mat... | DOI:\n \n \n\n 10.1186/s12014-024-09487-4 | https://pubmed.ncbi.nlm.nih.gov//38825704/ |
| 70 | A supervised, externally validated machine learning model for artifact and drainage detection in high-resolution intracranial pressure monitoring data | Objective:\n \n \n In neurocritical care, data from multiple biosensors are continuously measured, but only sporadically acknowledged by the attending physicians. In contrast, machine learning (ML) tools can analyze large amounts... | 2024 Mar 15;141(2):509-517. | J Neurosurg | Shufan Huo; Alexander Nelde; Christian Meisel; Franziska Scheibe; Andreas Meisel; Matthias Endres; Peter Vajkoczy; Stefan Wolf; Jan F Willms; Jens M Boss; Emanuela Keller; Shufan Huo; Shufan Huo; Alexander Nelde; Christian Meisel; Franziska Schei... | DOI:\n \n \n\n 10.3171/2023.12.JNS231670 | https://pubmed.ncbi.nlm.nih.gov//38489814/ |
| 71 | Comparing Amyloid Imaging Normalization Strategies for Alzheimer's Disease Classification using an Automated Machine Learning Pipeline | Amyloid imaging has been widely used in Alzheimer's disease (AD) diagnosis and biomarker discovery through detecting the regional amyloid plaque density. It is essential to be normalized by a reference region to reduce noise and artifacts. To exp... | 2023 Jun 16:2023:525-533. | AMIA Jt Summits Transl Sci Proc | Boning Tong; Shannon L Risacher; Jingxuan Bao; Yanbo Feng; Xinkai Wang; Marylyn D Ritchie; Jason H Moore; Ryan Urbanowicz; Andrew J Saykin; Li Shen; Boning Tong; Boning Tong; Shannon L Risacher; Jingxuan Bao; Yanbo Feng; Xinkai Wang; Marylyn D Ri... | None | https://pubmed.ncbi.nlm.nih.gov//37350880/ |
| 72 | Establishment and external validation of an early warning model of diabetic peripheral neuropathy based on random forest and logistic regression | Objective:\n \n \n The primary objective of this study was to investigate the risk factors for diabetic peripheral neuropathy (DPN) and to establish an early diagnostic prediction model for its onset, based on clinical data and b... | 2024 Sep 20;24(1):196. | BMC Endocr Disord | Lujie Wang; Jiajie Li; Yixuan Lin; Huilun Yuan; Zhaohui Fang; Aihua Fei; Guoming Shen; Aijuan Jiang; Lujie Wang; Lujie Wang; Jiajie Li; Yixuan Lin; Huilun Yuan; Zhaohui Fang; Aihua Fei; Guoming Shen; Aijuan Jiang | DOI:\n \n \n\n 10.1186/s12902-024-01728-9 | https://pubmed.ncbi.nlm.nih.gov//39304867/ |
| 73 | Experimental Investigation and Machine Learning Prediction of Mechanical Properties of Rubberized Concrete for Sustainable Construction | Concrete is widely used in civil engineering applications and the natural aggregates which used in concrete are scarce, but its demand is increasing. The disposal of rubber tyres poses a significant environmental challenge, as their decomposition... | 2024 Sep 30;14(1):22725. | Sci Rep | T Senthil Vadivel; Ardra Suseelan; K Karthick; Mejdl Safran; Sultan Alfarhood; T Senthil Vadivel; T Senthil Vadivel; Ardra Suseelan; K Karthick; Mejdl Safran; Sultan Alfarhood | DOI:\n \n \n\n 10.1038/s41598-024-73504-7 | https://pubmed.ncbi.nlm.nih.gov//39349571/ |
| 74 | Machine learning-assisted rapid determination for traditional Chinese Medicine Constitution | The aim of this study was to develop a machine learning-assisted rapid determination methodology for traditional Chinese Medicine Constitution. Based on the Constitution in Chinese Medicine Questionnaire (CCMQ), the most applied diagnostic instru... | 2024 Sep 15;19(1):127. | Chin Med | Wen Sun; Minghua Bai; Ji Wang; Bei Wang; Yixing Liu; Qi Wang; Dongran Han; Wen Sun; Wen Sun; Minghua Bai; Ji Wang; Bei Wang; Yixing Liu; Qi Wang; Dongran Han | DOI:\n \n \n\n 10.1186/s13020-024-00992-0 | https://pubmed.ncbi.nlm.nih.gov//39278905/ |
| 75 | Embedding covariate adjustments in tree-based automated machine learning for biomedical big data analyses | Background:\n \n \n A typical task in bioinformatics consists of identifying which features are associated with a target outcome of interest and building a predictive model. Automated machine learning (AutoML) systems such as the... | 2020 Oct 1;21(1):430. | BMC Bioinformatics | Elisabetta Manduchi; Weixuan Fu; Joseph D Romano; Stefano Ruberto; Jason H Moore; Elisabetta Manduchi; Elisabetta Manduchi; Weixuan Fu; Joseph D Romano; Stefano Ruberto; Jason H Moore | DOI:\n \n \n\n 10.1186/s12859-020-03755-4 | https://pubmed.ncbi.nlm.nih.gov//32998684/ |
| 76 | Development and validation of an automatic machine learning model to predict abnormal increase of transaminase in valproic acid-treated epilepsy | Valproic acid (VPA) is a primary medication for epilepsy, yet its hepatotoxicity consistently raises concerns among individuals. This study aims to establish an automated machine learning (autoML) model for forecasting the risk of abnormal increa... | 2024 Sep;98(9):3049-3061. | Arch Toxicol | Hongying Ma; Sihui Huang; Fengxin Li; Zicheng Pang; Jian Luo; Danfeng Sun; Junsong Liu; Zhuoming Chen; Jian Qu; Qiang Qu; Hongying Ma; Hongying Ma; Sihui Huang; Fengxin Li; Zicheng Pang; Jian Luo; Danfeng Sun; Junsong Liu; Zhuoming Chen; Jian Qu;... | DOI:\n \n \n\n 10.1007/s00204-024-03803-5 | https://pubmed.ncbi.nlm.nih.gov//38879852/ |
| 77 | Machine Learning for Antibiotic Resistance Prediction: A Prototype Using Off-the-Shelf Techniques and Entry-Level Data to Guide Empiric Antimicrobial Therapy | Objective:\n \n \n In the era of increasing antimicrobial resistance, the need for early identification and prompt treatment of multi-drug-resistant infections is crucial for achieving favorable outcomes in critically ill patient... | 2021 Jul;27(3):214-221. | Healthc Inform Res | Georgios Feretzakis; Aikaterini Sakagianni; Evangelos Loupelis; Dimitris Kalles; Nikoletta Skarmoutsou; Maria Martsoukou; Constantinos Christopoulos; Malvina Lada; Stavroula Petropoulou; Aikaterini Velentza; Sophia Michelidou; Rea Chatzikyriakou;... | DOI:\n \n \n\n 10.4258/hir.2021.27.3.214 | https://pubmed.ncbi.nlm.nih.gov//34384203/ |
| 78 | Modeling Users' Cognitive Performance Using Digital Pen Features | Digital pen features model characteristics of sketches and user behavior, and can be used for various supervised machine learning (ML) applications, such as multi-stroke sketch recognition and user modeling. In this work, we use a state-of-the-ar... | 2022 May 3:5:787179. | Front Artif Intell | Alexander Prange; Daniel Sonntag; Alexander Prange; Alexander Prange; Daniel Sonntag | DOI:\n \n \n\n 10.3389/frai.2022.787179 | https://pubmed.ncbi.nlm.nih.gov//35592648/ |
| 79 | Machine learning in the diagnosis, management, and care of patients with low back pain: a scoping review of the literature and future directions | Background context:\n \n \n Low back pain (LBP) remains the leading cause of disability globally. In recent years, machine learning (ML) has emerged as a potentially useful tool to aid the diagnosis, management, and prognosticati... | 2025 Jan;25(1):18-31. | Spine J | Andreas Seas; Tanner J Zachem; Bruno Valan; Christine Goertz; Shiva Nischal; Sully F Chen; David Sykes; Troy Q Tabarestani; Benjamin D Wissel; Elizabeth R Blackwood; Christopher Holland; Oren Gottfried; Christopher I Shaffrey; Muhammad M Abd-El-B... | DOI:\n \n \n\n 10.1016/j.spinee.2024.09.010 | https://pubmed.ncbi.nlm.nih.gov//39332687/ |
| 80 | Predicting stroke and myocardial infarction risk in Takayasu arteritis with automated machine learning models | Few models exist for predicting severe ischemic complications (SIC) in patients with Takayasu arteritis (TA). We conducted a retrospective analysis of 703 patients with TA from January 2010 to December 2019 to establish an SIC prediction model fo... | 2023 Nov 9;26(12):108421. | iScience | Yi-Ting Lu; Zeng-Lei Zhang; Xing-Yu Zhou; Di Zhang; Tao Tian; Peng Fan; Ying Zhang; Xian-Liang Zhou; Yi-Ting Lu; Yi-Ting Lu; Zeng-Lei Zhang; Xing-Yu Zhou; Di Zhang; Tao Tian; Peng Fan; Ying Zhang; Xian-Liang Zhou | DOI:\n \n \n\n 10.1016/j.isci.2023.108421 | https://pubmed.ncbi.nlm.nih.gov//38077132/ |
| 81 | The development and validation of automated machine learning models for predicting lymph node metastasis in Siewert type II T1 adenocarcinoma of the esophagogastric junction | Background:\n \n \n Lymph node metastasis (LNM) is considered an essential prognosis factor for adenocarcinoma of the esophagogastric junction (AEG), which also affects the treatment strategies of AEG. We aimed to evaluate automa... | 2024 Apr 3:11:1266278. | Front Med (Lausanne) | Chenghao Lu; Lu Liu; Minyue Yin; Jiaxi Lin; Shiqi Zhu; Jingwen Gao; Shuting Qu; Guoting Xu; Lihe Liu; Jinzhou Zhu; Chunfang Xu; Chenghao Lu; Chenghao Lu; Lu Liu; Minyue Yin; Jiaxi Lin; Shiqi Zhu; Jingwen Gao; Shuting Qu; Guoting Xu; Lihe Liu; Jin... | DOI:\n \n \n\n 10.3389/fmed.2024.1266278 | https://pubmed.ncbi.nlm.nih.gov//38633305/ |
| 82 | Performance of automated machine learning in detecting fundus diseases based on ophthalmologic B-scan ultrasound images | Aim:\n \n \n To evaluate the efficacy of automated machine learning (AutoML) models in detecting fundus diseases using ocular B-scan ultrasound images.\n \n\n\n Methods:\n \n \n Ophthalmologists annot... | 2024 Dec 11;9(1):e001873. | BMJ Open Ophthalmol | Qiaoling Wei; Qian Chen; Chen Zhao; Rui Jiang; Qiaoling Wei; Qiaoling Wei; Qian Chen; Chen Zhao; Rui Jiang | DOI:\n \n \n\n 10.1136/bmjophth-2024-001873 | https://pubmed.ncbi.nlm.nih.gov//39663141/ |
| 83 | Egg Freshness Prediction Model Using Real-Time Cold Chain Storage Condition Based on Transfer Learning | Maintaining and monitoring the quality of eggs is a major concern during cold chain storage and transportation due to the variation of external environments, such as temperature or humidity. In this study, we proposed a deep learning-based Haugh ... | 2022 Oct 5;11(19):3082. | Foods | Tae Hyong Kim; Jong Hoon Kim; Ji Young Kim; Seung Eel Oh; Tae Hyong Kim; Tae Hyong Kim; Jong Hoon Kim; Ji Young Kim; Seung Eel Oh | DOI:\n \n \n\n 10.3390/foods11193082 | https://pubmed.ncbi.nlm.nih.gov//36230158/ |
| 84 | A novel oppositional binary crow search algorithm with optimal machine learning based postpartum hemorrhage prediction model | Postpartum hemorrhage (PPH) is an obstetric emergency instigated by excessive blood loss which occurs frequently after the delivery. The PPH can result in volume depletion, hypovolemic shock, and anemia. This is particular condition is considered... | 2022 Jul 13;22(1):560. | BMC Pregnancy Childbirth | Sujatha Krishnamoorthy; Yihang Liu; Kun Liu; Sujatha Krishnamoorthy; Sujatha Krishnamoorthy; Yihang Liu; Kun Liu | DOI:\n \n \n\n 10.1186/s12884-022-04775-z | https://pubmed.ncbi.nlm.nih.gov//35831804/ |
| 85 | Exploring the influence of COVID-19 on the spread of hand, foot, and mouth disease with an automatic machine learning prediction model | Hand, foot, and mouth disease (HFMD) is an important public health problem and has received concern worldwide. Moreover, the coronavirus disease 2019 (COVID-19) epidemic also increases the difficulty of understanding and predicting the prevalence... | 2023 Feb;30(8):20369-20385. | Environ Sci Pollut Res Int | Chuan Yang; Shuyi An; Baojun Qiao; Peng Guan; Desheng Huang; Wei Wu; Chuan Yang; Chuan Yang; Shuyi An; Baojun Qiao; Peng Guan; Desheng Huang; Wei Wu | DOI:\n \n \n\n 10.1007/s11356-022-23643-z | https://pubmed.ncbi.nlm.nih.gov//36255582/ |
| 86 | Automated machine learning for the identification of asymptomatic COVID-19 carriers based on chest CT images | Background:\n \n \n Asymptomatic COVID-19 carriers with normal chest computed tomography (CT) scans have perpetuated the ongoing pandemic of this disease. This retrospective study aimed to use automated machine learning (AutoML) ... | 2024 Feb 27;24(1):50. | BMC Med Imaging | Minyue Yin; Chao Xu; Jinzhou Zhu; Yuhan Xue; Yijia Zhou; Yu He; Jiaxi Lin; Lu Liu; Jingwen Gao; Xiaolin Liu; Dan Shen; Cuiping Fu; Minyue Yin; Minyue Yin; Chao Xu; Jinzhou Zhu; Yuhan Xue; Yijia Zhou; Yu He; Jiaxi Lin; Lu Liu; Jingwen Gao; Xiaolin... | DOI:\n \n \n\n 10.1186/s12880-024-01211-w | https://pubmed.ncbi.nlm.nih.gov//38413923/ |
| 87 | Application of interpretable machine learning models to improve the prediction performance of ionic liquids toxicity | With the wide application prospect of ionic liquids (ILs) as solvent in the future industry, in order to promote green and sustainable chemical engineering, the toxicity problem of common concern has been systematically modeled. Machine learning ... | 2024 Jan 15:908:168168. | Sci Total Environ | Dingchao Fan; Ke Xue; Runqi Zhang; Wenguang Zhu; Hongru Zhang; Jianguang Qi; Zhaoyou Zhu; Yinglong Wang; Peizhe Cui; Dingchao Fan; Dingchao Fan; Ke Xue; Runqi Zhang; Wenguang Zhu; Hongru Zhang; Jianguang Qi; Zhaoyou Zhu; Yinglong Wang; Peizhe Cui | DOI:\n \n \n\n 10.1016/j.scitotenv.2023.168168 | https://pubmed.ncbi.nlm.nih.gov//37918734/ |
| 88 | Evaluating Binary Classifiers for Cardiovascular Disease Prediction: Enhancing Early Diagnostic Capabilities | Cardiovascular disease (CVD) is a significant global health concern and the leading cause of death in many countries. Early detection and diagnosis of CVD can significantly reduce the risk of complications and mortality. Machine learning methods,... | 2024 Dec 9;11(12):396. | J Cardiovasc Dev Dis | Paul Iacobescu; Virginia Marina; Catalin Anghel; Aurelian-Dumitrache Anghele; Paul Iacobescu; Paul Iacobescu; Virginia Marina; Catalin Anghel; Aurelian-Dumitrache Anghele | DOI:\n \n \n\n 10.3390/jcdd11120396 | https://pubmed.ncbi.nlm.nih.gov//39728286/ |
| 89 | Using machine learning methods to predict electric vehicles penetration in the automotive market | Electric vehicles (EVs) have been introduced as an alternative to gasoline and diesel cars to reduce greenhouse gas emissions, optimize fossil fuel use, and protect the environment. Predicting EV sales is momentous for stakeholders, including car... | 2023 May 23;13(1):8345. | Sci Rep | Shahriar Afandizadeh; Diyako Sharifi; Navid Kalantari; Hamid Mirzahossein; Shahriar Afandizadeh; Shahriar Afandizadeh; Diyako Sharifi; Navid Kalantari; Hamid Mirzahossein | DOI:\n \n \n\n 10.1038/s41598-023-35366-3 | https://pubmed.ncbi.nlm.nih.gov//37221231/ |
| 90 | Accurate Blood-Based Diagnostic Biosignatures for Alzheimer's Disease via Automated Machine Learning | Alzheimer's disease (AD) is the most common form of neurodegenerative dementia and its timely diagnosis remains a major challenge in biomarker discovery. In the present study, we analyzed publicly available high-throughput low-sample -omics datas... | 2020 Sep 18;9(9):3016. | J Clin Med | Makrina Karaglani; Krystallia Gourlia; Ioannis Tsamardinos; Ekaterini Chatzaki; Makrina Karaglani; Makrina Karaglani; Krystallia Gourlia; Ioannis Tsamardinos; Ekaterini Chatzaki | DOI:\n \n \n\n 10.3390/jcm9093016 | https://pubmed.ncbi.nlm.nih.gov//32962113/ |
| 91 | Machine learning approach for the detection of vitamin D level: a comparative study | Background:\n \n \n After the World Health Organization declared the COVID-19 pandemic, the role of Vitamin D has become even more critical for people worldwide. The most accurate way to define vitamin D level is 25-hydroxy vitam... | 2023 Oct 16;23(1):219. | BMC Med Inform Decis Mak | Nuriye Sancar; Sahar S Tabrizi; Nuriye Sancar; Nuriye Sancar; Sahar S Tabrizi | DOI:\n \n \n\n 10.1186/s12911-023-02323-z | https://pubmed.ncbi.nlm.nih.gov//37845674/ |
| 92 | Multi-strategy modified sparrow search algorithm for hyperparameter optimization in arbitrage prediction models | Deep learning models struggle to effectively capture data features and make accurate predictions because of the strong non-linear characteristics of arbitrage data. Therefore, to fully exploit the model performance, researchers have focused on ne... | 2024 May 15;19(5):e0303688. | PLoS One | Shenjie Cheng; Panke Qin; Baoyun Lu; Jinxia Yu; Yongli Tang; Zeliang Zeng; Sensen Tu; Haoran Qi; Bo Ye; Zhongqi Cai; Shenjie Cheng; Shenjie Cheng; Panke Qin; Baoyun Lu; Jinxia Yu; Yongli Tang; Zeliang Zeng; Sensen Tu; Haoran Qi; Bo Ye; Zhongqi Cai | DOI:\n \n \n\n 10.1371/journal.pone.0303688 | https://pubmed.ncbi.nlm.nih.gov//38748753/ |
| 93 | R-MFE-TCN: A correlation prediction model between body surface and tumor during respiratory movement | Background:\n \n \n 2D CT image-guided radiofrequency ablation (RFA) is an exciting minimally invasive treatment that can destroy liver tumors without removing them. However, CT images can only provide limited static information,... | 2024 Sep;51(9):6075-6089. | Med Phys | Xuehu Wang; Chang Yang; Ziqi Liu; Jushuo Zhang; Chao Xue; Lihong Xing; Yongchang Zheng; Chen Geng; Xiaoping Yin; Xuehu Wang; Xuehu Wang; Chang Yang; Ziqi Liu; Jushuo Zhang; Chao Xue; Lihong Xing; Yongchang Zheng; Chen Geng; Xiaoping Yin | DOI:\n \n \n\n 10.1002/mp.17183 | https://pubmed.ncbi.nlm.nih.gov//38801342/ |
| 94 | The predictive machine learning model of a hydrated inverse vulcanized copolymer for effective mercury sequestration from wastewater | Inverse vulcanized polysulfides (IVP) are promising sulfur-enriched copolymers with unconventional properties irresistible for diverse applications like Hg2+ remediation. Nevertheless, due to their inherent hydrophobic nature, these copolymers st... | 2024 Jan 15:908:168034. | Sci Total Environ | Ali Shaan Manzoor Ghumman; Rashid Shamsuddin; Amin Abbasi; Mohaira Ahmad; Yoshiaki Yoshida; Abdul Sami; Hamad Almohamadi; Ali Shaan Manzoor Ghumman; Ali Shaan Manzoor Ghumman; Rashid Shamsuddin; Amin Abbasi; Mohaira Ahmad; Yoshiaki Yoshida; Abdul... | DOI:\n \n \n\n 10.1016/j.scitotenv.2023.168034 | https://pubmed.ncbi.nlm.nih.gov//37924888/ |
| 95 | Application of multimodal deep learning and multi-instance learning fusion techniques in predicting STN-DBS outcomes for Parkinson's disease patients | Parkinson's Disease (PD) is a progressive neurodegenerative disorder with substantial impact on patients' quality of life. Subthalamic nucleus deep brain stimulation (STN-DBS) is an effective treatment for advanced PD, but patient responses vary,... | 2024 Oct;21(6):e00471. | Neurotherapeutics | Bowen Chang; Zhi Geng; Jiaming Mei; Zhengyu Wang; Peng Chen; Yuge Jiang; Chaoshi Niu; Bowen Chang; Bowen Chang; Zhi Geng; Jiaming Mei; Zhengyu Wang; Peng Chen; Yuge Jiang; Chaoshi Niu | DOI:\n \n \n\n 10.1016/j.neurot.2024.e00471 | https://pubmed.ncbi.nlm.nih.gov//39419638/ |
| 96 | Mantis-ml: Disease-Agnostic Gene Prioritization from High-Throughput Genomic Screens by Stochastic Semi-supervised Learning | Access to large-scale genomics datasets has increased the utility of hypothesis-free genome-wide analyses. However, gene signals are often insufficiently powered to reach experiment-wide significance, triggering a process of laborious triaging of... | 2020 May 7;106(5):659-678. | Am J Hum Genet | Dimitrios Vitsios; Slavé Petrovski; Dimitrios Vitsios; Dimitrios Vitsios; Slavé Petrovski | DOI:\n \n \n\n 10.1016/j.ajhg.2020.03.012 | https://pubmed.ncbi.nlm.nih.gov//32386536/ |
| 97 | Clinician-Driven AI: Code-Free Self-Training on Public Data for Diabetic Retinopathy Referral | Importance:\n \n \n Democratizing artificial intelligence (AI) enables model development by clinicians with a lack of coding expertise, powerful computing resources, and large, well-labeled data sets.\n \n\n\n Objecti... | 2023 Nov 1;141(11):1029-1036. | JAMA Ophthalmol | Edward Korot; Mariana Batista Gonçalves; Josef Huemer; Sara Beqiri; Hagar Khalid; Madeline Kelly; Mark Chia; Emily Mathijs; Robbert Struyven; Magdy Moussa; Pearse A Keane; Edward Korot; Edward Korot; Mariana Batista Gonçalves; Josef Huemer; Sara ... | DOI:\n \n \n\n 10.1001/jamaophthalmol.2023.4508 | https://pubmed.ncbi.nlm.nih.gov//37856110/ |
| 98 | High-dimensional multinomial multiclass severity scoring of COVID-19 pneumonia using CT radiomics features and machine learning algorithms | We aimed to construct a prediction model based on computed tomography (CT) radiomics features to classify COVID-19 patients into severe-, moderate-, mild-, and non-pneumonic. A total of 1110 patients were studied from a publicly available dataset... | 2022 Sep 1;12(1):14817. | Sci Rep | Isaac Shiri; Shayan Mostafaei; Atlas Haddadi Avval; Yazdan Salimi; Amirhossein Sanaat; Azadeh Akhavanallaf; Hossein Arabi; Arman Rahmim; Habib Zaidi; Isaac Shiri; Isaac Shiri; Shayan Mostafaei; Atlas Haddadi Avval; Yazdan Salimi; Amirhossein Sana... | DOI:\n \n \n\n 10.1038/s41598-022-18994-z | https://pubmed.ncbi.nlm.nih.gov//36050434/ |
| 99 | Prediction of Liver Enzyme Elevation Using Supervised Machine Learning in Patients With Rheumatoid Arthritis on Treatment with Methotrexate | Objective The aim of this study is to develop a machine learning (ML) model to accurately predict liver enzyme elevation in rheumatoid arthritis (RA) patients on treatment with methotrexate (MTX) using electronic health record (EHR) data from a r... | 2024 Jan 11;16(1):e52110. | Cureus | Sandeep Surendran; Mithun C B; Vinit Gilvaz; Prudhvi K Manyam; Kavya Panicker; Manu Pradeep; Sandeep Surendran; Sandeep Surendran; Mithun C B; Vinit Gilvaz; Prudhvi K Manyam; Kavya Panicker; Manu Pradeep | DOI:\n \n \n\n 10.7759/cureus.52110 | https://pubmed.ncbi.nlm.nih.gov//38344615/ |
| 100 | Do AutoML-Based QSAR Models Fulfill OECD Principles for Regulatory Assessment? A 5-HT1A Receptor Case | The drug discovery and development process requires a lot of time, financial, and workforce resources. Any reduction in these burdens might benefit all stakeholders in the healthcare domain, including patients, government, and companies. One of t... | 2022 Jul 6;14(7):1415. | Pharmaceutics | Natalia Czub; Adam Pacławski; Jakub Szlęk; Aleksander Mendyk; Natalia Czub; Natalia Czub; Adam Pacławski; Jakub Szlęk; Aleksander Mendyk | DOI:\n \n \n\n 10.3390/pharmaceutics14071415 | https://pubmed.ncbi.nlm.nih.gov//35890310/ |
| 101 | Deciphering the Methylation Landscape in Breast Cancer: Diagnostic and Prognostic Biosignatures through Automated Machine Learning | DNA methylation plays an important role in breast cancer (BrCa) pathogenesis and could contribute to driving its personalized management. We performed a complete bioinformatic analysis in BrCa whole methylome datasets, analyzed using the Illumina... | 2021 Apr 2;13(7):1677. | Cancers (Basel) | Maria Panagopoulou; Makrina Karaglani; Vangelis G Manolopoulos; Ioannis Iliopoulos; Ioannis Tsamardinos; Ekaterini Chatzaki; Maria Panagopoulou; Maria Panagopoulou; Makrina Karaglani; Vangelis G Manolopoulos; Ioannis Iliopoulos; Ioannis Tsamardin... | DOI:\n \n \n\n 10.3390/cancers13071677 | https://pubmed.ncbi.nlm.nih.gov//33918195/ |
| 102 | Prediction of BRAF V600E variant from cancer gene expression data | Background:\n \n \n BRAF inhibitors have been approved for the treatment of melanoma, non-small cell lung cancer, and colon cancer. Real-time polymerase chain reaction or next-generation sequencing were clinically used for BRAF v... | 2022 Nov;11(11):4051-4056. | Transl Cancer Res | Jun Kang; Jieun Lee; Ahwon Lee; Youn Soo Lee; Jun Kang; Jun Kang; Jieun Lee; Ahwon Lee; Youn Soo Lee | DOI:\n \n \n\n 10.21037/tcr-22-883 | https://pubmed.ncbi.nlm.nih.gov//36523293/ |
| 103 | A Bibliometric Analysis and Benchmark of Machine Learning and AutoML in Crash Severity Prediction: The Case Study of Three Colombian Cities | Traffic accidents are of worldwide concern, as they are one of the leading causes of death globally. One policy designed to cope with them is the design and deployment of road safety systems. These aim to predict crashes based on historical recor... | 2021 Dec 16;21(24):8401. | Sensors (Basel) | Juan S Angarita-Zapata; Gina Maestre-Gongora; Jenny Fajardo Calderín; Juan S Angarita-Zapata; Juan S Angarita-Zapata; Gina Maestre-Gongora; Jenny Fajardo Calderín | DOI:\n \n \n\n 10.3390/s21248401 | https://pubmed.ncbi.nlm.nih.gov//34960494/ |
| 104 | Prediction of Ecofriendly Concrete Compressive Strength Using Gradient Boosting Regression Tree Combined with GridSearchCV Hyperparameter-Optimization Techniques | A crucial factor in the efficient design of concrete sustainable buildings is the compressive strength (Cs) of eco-friendly concrete. In this work, a hybrid model of Gradient Boosting Regression Tree (GBRT) with grid search cross-validation (Grid... | 2022 Oct 23;15(21):7432. | Materials (Basel) | Zaineb M Alhakeem; Yasir Mohammed Jebur; Sadiq N Henedy; Hamza Imran; Luís F A Bernardo; Hussein M Hussein; Zaineb M Alhakeem; Zaineb M Alhakeem; Yasir Mohammed Jebur; Sadiq N Henedy; Hamza Imran; Luís F A Bernardo; Hussein M Hussein | DOI:\n \n \n\n 10.3390/ma15217432 | https://pubmed.ncbi.nlm.nih.gov//36363023/ |
| 105 | A framework for prediction of personalized pediatric nuclear medical dosimetry based on machine learning and Monte Carlo techniques | Objective:A methodology is introduced for the development of an internal dosimetry prediction toolkit for nuclear medical pediatric applications. The proposed study exploits Artificial Intelligence techniques using Monte Carlo simulations as grou... | 2023 Apr 7;68(8). | Phys Med Biol | Vasileios Eleftheriadis; Georgios Savvidis; Valentina Paneta; Konstantinos Chatzipapas; George C Kagadis; Panagiotis Papadimitroulas; Vasileios Eleftheriadis; Vasileios Eleftheriadis; Georgios Savvidis; Valentina Paneta; Konstantinos Chatzipapas;... | DOI:\n \n \n\n 10.1088/1361-6560/acc4a5 | https://pubmed.ncbi.nlm.nih.gov//36921349/ |
| 106 | DeepMicro: deep representation learning for disease prediction based on microbiome data | Human microbiota plays a key role in human health and growing evidence supports the potential use of microbiome as a predictor of various diseases. However, the high-dimensionality of microbiome data, often in the order of hundreds of thousands, ... | 2020 Apr 7;10(1):6026. | Sci Rep | Min Oh; Liqing Zhang; Min Oh; Min Oh; Liqing Zhang | DOI:\n \n \n\n 10.1038/s41598-020-63159-5 | https://pubmed.ncbi.nlm.nih.gov//32265477/ |
| 107 | In Silico Prediction of Fraction Unbound in Human Plasma from Chemical Fingerprint Using Automated Machine Learning | Predicting the fraction unbound of a drug in plasma plays a significant role in understanding its pharmacokinetic properties during in vitro studies of drug design and discovery. Owing to the gaining reliability of machine learning in biological ... | 2021 Mar 5;6(10):6791-6797. | ACS Omega | Viswajit Mulpuru; Nidhi Mishra; Viswajit Mulpuru; Viswajit Mulpuru; Nidhi Mishra | DOI:\n \n \n\n 10.1021/acsomega.0c05846 | https://pubmed.ncbi.nlm.nih.gov//33748592/ |
| 108 | Test-time bi-directional adaptation between image and model for robust segmentation | Background and objective:\n \n \n Deep learning models often suffer from performance degradations when deployed in real clinical environments due to appearance shifts between training and testing images. Most extant methods use t... | 2023 May:233:107477. | Comput Methods Programs Biomed | Xiaoqiong Huang; Xin Yang; Haoran Dou; Yuhao Huang; Li Zhang; Zhendong Liu; Zhongnuo Yan; Lian Liu; Yuxin Zou; Xindi Hu; Rui Gao; Yuanji Zhang; Yi Xiong; Wufeng Xue; Dong Ni; Xiaoqiong Huang; Xiaoqiong Huang; Xin Yang; Haoran Dou; Yuhao Huang; Li... | DOI:\n \n \n\n 10.1016/j.cmpb.2023.107477 | https://pubmed.ncbi.nlm.nih.gov//36972645/ |
| 109 | Long-term prediction modeling of shallow rockburst with small dataset based on machine learning | Rockburst present substantial hazards in both deep underground construction and shallow depths, underscoring the critical need for accurate prediction methods. This study addressed this need by collecting and analyzing 69 real datasets of rockbur... | 2024 Jul 12;14(1):16131. | Sci Rep | Guozhu Rao; Yunzhang Rao; Jiazheng Wan; Qiang Huang; Yangjun Xie; Qiande Lai; Zhihua Yang; Run Xiang; Laiye Zhang; Guozhu Rao; Guozhu Rao; Yunzhang Rao; Jiazheng Wan; Qiang Huang; Yangjun Xie; Qiande Lai; Zhihua Yang; Run Xiang; Laiye Zhang | DOI:\n \n \n\n 10.1038/s41598-024-64107-3 | https://pubmed.ncbi.nlm.nih.gov//38997304/ |
| 110 | Transfer Learning Video Classification of Preserved, Mid-Range, and Reduced Left Ventricular Ejection Fraction in Echocardiography | Identifying patients with left ventricular ejection fraction (EF), either reduced [EF < 40% (rEF)], mid-range [EF 40-50% (mEF)], or preserved [EF > 50% (pEF)], is considered of primary clinical importance. An end-to-end video classification using... | 2024 Jul 5;14(13):1439. | Diagnostics (Basel) | Pierre Decoodt; Daniel Sierra-Sosa; Laura Anghel; Giovanni Cuminetti; Eva De Keyzer; Marielle Morissens; Pierre Decoodt; Pierre Decoodt; Daniel Sierra-Sosa; Laura Anghel; Giovanni Cuminetti; Eva De Keyzer; Marielle Morissens | DOI:\n \n \n\n 10.3390/diagnostics14131439 | https://pubmed.ncbi.nlm.nih.gov//39001328/ |
| 111 | A data-driven binary-classification framework for oil fingerprinting analysis | A marine oil spill is one of the most challenging environmental issues, resulting in severe long-term impacts on ecosystems and human society. Oil dispersants are widely applied as a treating agent in oil spill response operations. The usage of d... | 2021 Oct:201:111454. | Environ Res | Yifu Chen; Bing Chen; Xing Song; Qiao Kang; Xudong Ye; Baiyu Zhang; Yifu Chen; Yifu Chen; Bing Chen; Xing Song; Qiao Kang; Xudong Ye; Baiyu Zhang | DOI:\n \n \n\n 10.1016/j.envres.2021.111454 | https://pubmed.ncbi.nlm.nih.gov//34111437/ |
| 112 | Automated Machine Learning Tools to Build Regression Models for Schizosaccharomyces pombe Omics Data | Machine learning is a powerful tool for analyzing biological data and making useful predictions. The surge of biological data from high-throughput omics technologies has raised the need for modeling approaches capable of tackling such amounts of ... | 2025:2862:353-361. | Methods Mol Biol | Mauricio Alexander de Moura Ferreira; Wendel Batista da Silveira; Mauricio Alexander de Moura Ferreira; Mauricio Alexander de Moura Ferreira; Wendel Batista da Silveira | DOI:\n \n \n\n 10.1007/978-1-0716-4168-2\_25 | https://pubmed.ncbi.nlm.nih.gov//39527213/ |
| 113 | A novel melanoma prediction model for imbalanced data using optimized SqueezeNet by bald eagle search optimization | Skin lesion classification plays a crucial role in diagnosing various gene and related local medical cases in the field of dermoscopy. In this paper, a new model for the classification of skin lesions as either normal or melanoma is presented. Th... | 2021 Sep:136:104712. | Comput Biol Med | Gehad Ismail Sayed; Mona M Soliman; Aboul Ella Hassanien; Gehad Ismail Sayed; Gehad Ismail Sayed; Mona M Soliman; Aboul Ella Hassanien | DOI:\n \n \n\n 10.1016/j.compbiomed.2021.104712 | https://pubmed.ncbi.nlm.nih.gov//34388470/ |
| 114 | Machine Learning-Based Risk Factor Analysis and Prediction Model Construction for the Occurrence of Chronic Heart Failure: Health Ecologic Study | Background:\n \n \n Chronic heart failure (CHF) is a serious threat to human health, with high morbidity and mortality rates, imposing a heavy burden on the health care system and society. With the abundance of medical data and t... | 2025 Jan 31:13:e64972. | JMIR Med Inform | Qian Xu; Xue Cai; Ruicong Yu; Yueyue Zheng; Guanjie Chen; Hui Sun; Tianyun Gao; Cuirong Xu; Jing Sun; Qian Xu; Qian Xu; Xue Cai; Ruicong Yu; Yueyue Zheng; Guanjie Chen; Hui Sun; Tianyun Gao; Cuirong Xu; Jing Sun | DOI:\n \n \n\n 10.2196/64972 | https://pubmed.ncbi.nlm.nih.gov//39889299/ |
| 115 | AlphaML: A clear, legible, explainable, transparent, and elucidative binary classification platform for tabular data | Leveraging the potential of machine learning and recognizing the broad applications of binary classification, it becomes essential to develop platforms that are not only powerful but also transparent, interpretable, and user friendly. We introduc... | 2023 Dec 13;5(1):100897. | Patterns (N Y) | Ahmad Nasimian; Saleena Younus; Özge Tatli; Emma U Hammarlund; Kenneth J Pienta; Lars Rönnstrand; Julhash U Kazi; Ahmad Nasimian; Ahmad Nasimian; Saleena Younus; Özge Tatli; Emma U Hammarlund; Kenneth J Pienta; Lars Rönnstrand; Julhash U Kazi | DOI:\n \n \n\n 10.1016/j.patter.2023.100897 | https://pubmed.ncbi.nlm.nih.gov//38264719/ |
| 116 | Prediction of pathologic complete response to neoadjuvant chemotherapy using machine learning models in patients with breast cancer | Background:\n \n \n The aim of this study was to develop a machine learning (ML) based model to accurately predict pathologic complete response (pCR) to neoadjuvant chemotherapy (NAC) using pretreatment clinical and pathological ... | 2021 Oct;189(3):747-757. | Breast Cancer Res Treat | Ji-Yeon Kim; Eunjoo Jeon; Soonhwan Kwon; Hyungsik Jung; Sunghoon Joo; Youngmin Park; Se Kyung Lee; Jeong Eon Lee; Seok Jin Nam; Eun Yoon Cho; Yeon Hee Park; Jin Seok Ahn; Young-Hyuck Im; Ji-Yeon Kim; Ji-Yeon Kim; Eunjoo Jeon; Soonhwan Kwon; Hyung... | DOI:\n \n \n\n 10.1007/s10549-021-06310-8 | https://pubmed.ncbi.nlm.nih.gov//34224056/ |
| 117 | Development of ANN-Based Warpage Prediction Model for FCCSP via Subdomain Sampling and Taguchi Hyperparameter Optimization | This study aims to establish an accurate prediction model using artificial neural networks (ANNs) to effectively and efficiently predict the process-induced warpage of a flip-chip chip-scale package (FCCSP). To enhance model performance, a novel ... | 2023 Jun 28;14(7):1325. | Micromachines (Basel) | Hsien-Chie Cheng; Chia-Lin Ma; Yang-Lun Liu; Hsien-Chie Cheng; Hsien-Chie Cheng; Chia-Lin Ma; Yang-Lun Liu | DOI:\n \n \n\n 10.3390/mi14071325 | https://pubmed.ncbi.nlm.nih.gov//37512636/ |
| 118 | Improved SVM-Based Soil-Moisture-Content Prediction Model for Tea Plantation | Accurate prediction of soil moisture content in tea plantations plays a crucial role in optimizing irrigation practices and improving crop productivity. Traditional methods for SMC prediction are difficult to implement due to high costs and labor... | 2023 Jun 14;12(12):2309. | Plants (Basel) | Ying Huang; Ying Huang; Ying Huang | DOI:\n \n \n\n 10.3390/plants12122309 | https://pubmed.ncbi.nlm.nih.gov//37375934/ |
| 119 | Prediction of medication-related osteonecrosis of the jaw (MRONJ) using automated machine learning in patients with osteoporosis associated with dental extraction and implantation: a retrospective study | Objectives:\n \n \n This study aimed to develop and validate machine learning (ML) models using H2O-AutoML, an automated ML program, for predicting medication-related osteonecrosis of the jaw (MRONJ) in patients with osteoporosis... | 2023 Jun 30;49(3):135-141. | J Korean Assoc Oral Maxillofac Surg | Da Woon Kwack; Sung Min Park; Da Woon Kwack; Da Woon Kwack; Sung Min Park | DOI:\n \n \n\n 10.5125/jkaoms.2023.49.3.135 | https://pubmed.ncbi.nlm.nih.gov//37394932/ |
| 120 | A deep learning-based approach for the diagnosis of adrenal adenoma: a new trial using CT | Objective:\n \n \n To develop and validate deep convolutional neural network (DCNN) models for the diagnosis of adrenal adenoma (AA) using CT.\n \n\n\n Methods:\n \n \n This retrospective study enroll... | 2022 Jul 1;95(1135):20211066. | Br J Radiol | Masaoki Kusunoki; Tomohiro Nakayama; Akihiro Nishie; Yasuo Yamashita; Kazufumi Kikuchi; Masatoshi Eto; Yoshinao Oda; Kousei Ishigami; Masaoki Kusunoki; Masaoki Kusunoki; Tomohiro Nakayama; Akihiro Nishie; Yasuo Yamashita; Kazufumi Kikuchi; Masato... | DOI:\n \n \n\n 10.1259/bjr.20211066 | https://pubmed.ncbi.nlm.nih.gov//35522787/ |
| 121 | Predicting Depression From Smartphone Behavioral Markers Using Machine Learning Methods, Hyperparameter Optimization, and Feature Importance Analysis: Exploratory Study | Background:\n \n \n Depression is a prevalent mental health challenge. Current depression assessment methods using self-reported and clinician-administered questionnaires have limitations. Instrumenting smartphones to passively a... | 2021 Jul 12;9(7):e26540. | JMIR Mhealth Uhealth | Kennedy Opoku Asare; Yannik Terhorst; Julio Vega; Ella Peltonen; Eemil Lagerspetz; Denzil Ferreira; Kennedy Opoku Asare; Kennedy Opoku Asare; Yannik Terhorst; Julio Vega; Ella Peltonen; Eemil Lagerspetz; Denzil Ferreira | DOI:\n \n \n\n 10.2196/26540 | https://pubmed.ncbi.nlm.nih.gov//34255713/ |
| 122 | Clustering-based binary Grey Wolf Optimisation model with 6LDCNNet for prediction of heart disease using patient data | In recent years, the healthcare data system has expanded rapidly, allowing for the identification of important health trends and facilitating targeted preventative care. Heart disease remains a leading cause of death in developed countries, often... | 2025 Jan 8;15(1):1270. | Sci Rep | Lella Kranthi Kumar; K G Suma; Pamula Udayaraju; Venkateswarlu Gundu; Srihari Varma Mantena; B N Jagadesh; Lella Kranthi Kumar; Lella Kranthi Kumar; K G Suma; Pamula Udayaraju; Venkateswarlu Gundu; Srihari Varma Mantena; B N Jagadesh | DOI:\n \n \n\n 10.1038/s41598-025-85561-7 | https://pubmed.ncbi.nlm.nih.gov//39779935/ |
| 123 | Prediction of 30-day mortality in heart failure patients with hypoxic hepatitis: Development and external validation of an interpretable machine learning model | Background:\n \n \n This study aimed to explore the impact of hypoxic hepatitis (HH) on survival in heart failure (HF) patients and to develop an effective machine learning model to predict 30-day mortality risk in HF patients wi... | 2022 Oct 28:9:1035675. | Front Cardiovasc Med | Run Sun; Xue Wang; Haiyan Jiang; Yan Yan; Yansong Dong; Wenxiao Yan; Xinye Luo; Hua Miu; Lei Qi; Zhongwei Huang; Run Sun; Run Sun; Xue Wang; Haiyan Jiang; Yan Yan; Yansong Dong; Wenxiao Yan; Xinye Luo; Hua Miu; Lei Qi; Zhongwei Huang | DOI:\n \n \n\n 10.3389/fcvm.2022.1035675 | https://pubmed.ncbi.nlm.nih.gov//36386374/ |
| 124 | Phenotyping the Histopathological Subtypes of Non-Small-Cell Lung Carcinoma: How Beneficial Is Radiomics? | The aim of this study was to investigate the usefulness of radiomics in the absence of well-defined standard guidelines. Specifically, we extracted radiomics features from multicenter computed tomography (CT) images to differentiate between the f... | 2023 Mar 18;13(6):1167. | Diagnostics (Basel) | Giovanni Pasini; Alessandro Stefano; Giorgio Russo; Albert Comelli; Franco Marinozzi; Fabiano Bini; Giovanni Pasini; Giovanni Pasini; Alessandro Stefano; Giorgio Russo; Albert Comelli; Franco Marinozzi; Fabiano Bini | DOI:\n \n \n\n 10.3390/diagnostics13061167 | https://pubmed.ncbi.nlm.nih.gov//36980475/ |
| 125 | Detecting pulmonary malignancy against benign nodules using noninvasive cell-free DNA fragmentomics assay | Background:\n \n \n Early screening using low-dose computed tomography (LDCT) can reduce mortality caused by non-small-cell lung cancer. However, ∼25% of the 'suspicious' pulmonary nodules identified by LDCT are later confirmed b... | 2024 Aug;9(8):103595. | ESMO Open | S Xu; J Luo; W Tang; H Bao; J Wang; S Chang; Z Zou; X Fan; Y Liu; C Jiang; X Wu; S Xu; S Xu; J Luo; W Tang; H Bao; J Wang; S Chang; Z Zou; X Fan; Y Liu; C Jiang; X Wu | DOI:\n \n \n\n 10.1016/j.esmoop.2024.103595 | https://pubmed.ncbi.nlm.nih.gov//39088983/ |
| 126 | NYUS.2: an automated machine learning prediction model for the large-scale real-time simulation of grapevine freezing tolerance in North America | Accurate and real-time monitoring of grapevine freezing tolerance is crucial for the sustainability of the grape industry in cool climate viticultural regions. However, on-site data are limited due to the complexity of measurement. Current predic... | 2023 Dec 29;11(2):uhad286. | Hortic Res | Hongrui Wang; Gaurav D Moghe; Al P Kovaleski; Markus Keller; Timothy E Martinson; A Harrison Wright; Jeffrey L Franklin; Andréanne Hébert-Haché; Caroline Provost; Michael Reinke; Amaya Atucha; Michael G North; Jennifer P Russo; Pierre Helwi; Mich... | DOI:\n \n \n\n 10.1093/hr/uhad286 | https://pubmed.ncbi.nlm.nih.gov//38487294/ |
| 127 | A training pipeline of an arrhythmia classifier for atrial fibrillation detection using Photoplethysmography signal | Photoplethysmography (PPG) signal is potentially suitable in atrial fibrillation (AF) detection for its convenience in use and similarity in physiological origin to electrocardiogram (ECG). There are a few preceding studies that have shown the po... | 2023 Jan 19:14:1084837. | Front Physiol | Sota Kudo; Zheng Chen; Xue Zhou; Leighton T Izu; Ye Chen-Izu; Xin Zhu; Toshiyo Tamura; Shigehiko Kanaya; Ming Huang; Sota Kudo; Sota Kudo; Zheng Chen; Xue Zhou; Leighton T Izu; Ye Chen-Izu; Xin Zhu; Toshiyo Tamura; Shigehiko Kanaya; Ming Huang | DOI:\n \n \n\n 10.3389/fphys.2023.1084837 | https://pubmed.ncbi.nlm.nih.gov//36744032/ |
| 128 | Development and Validation of an ICU-Venous Thromboembolism Prediction Model Using Machine Learning Approaches: A Multicenter Study | Purpose:\n \n \n The purpose of this study was to establish and validate machine learning-based models for predicting the risk of venous thromboembolism (VTE) in intensive care unit (ICU) patients.\n \n\n\n Patients a... | 2024 Jul 24:17:3279-3292. | Int J Gen Med | Jie Jin; Jie Lu; Xinyang Su; Yinhuan Xiong; Shasha Ma; Yang Kong; Hongmei Xu; Jie Jin; Jie Jin; Jie Lu; Xinyang Su; Yinhuan Xiong; Shasha Ma; Yang Kong; Hongmei Xu | DOI:\n \n \n\n 10.2147/IJGM.S467374 | https://pubmed.ncbi.nlm.nih.gov//39070227/ |
| 129 | Systemic lupus erythematosus with high disease activity identification based on machine learning | Objective:\n \n \n Clinical evaluation of systemic lupus erythematosus (SLE) disease activity is limited and inconsistent, and high disease activity significantly, seriously impacts on SLE patients. This study aims to generate a ... | 2023 Sep;72(9):1909-1918. | Inflamm Res | Da-Cheng Wang; Wang-Dong Xu; Zhen Qin; Lu Fu; You-Yu Lan; Xiao-Yan Liu; An-Fang Huang; Da-Cheng Wang; Da-Cheng Wang; Wang-Dong Xu; Zhen Qin; Lu Fu; You-Yu Lan; Xiao-Yan Liu; An-Fang Huang | DOI:\n \n \n\n 10.1007/s00011-023-01793-1 | https://pubmed.ncbi.nlm.nih.gov//37725103/ |
| 130 | Employing advanced supervised machine learning approaches for predicting micronutrient intake status among children aged 6-23 months in Ethiopia | Background:\n \n \n Although micronutrients (MNs) are important for children's growth and development, their intake has not received enough attention. MN deficiency is a significant public health problem, especially in developing... | 2024 Jun 11:11:1397399. | Front Nutr | Alemu Birara Zemariam; Molalign Aligaz Adisu; Aklilu Abera Habesse; Biruk Beletew Abate; Molla Azmeraw Bizuayehu; Wubet Tazeb Wondie; Addis Wondmagegn Alamaw; Habtamu Setegn Ngusie; Alemu Birara Zemariam; Alemu Birara Zemariam; Molalign Aligaz Ad... | DOI:\n \n \n\n 10.3389/fnut.2024.1397399 | https://pubmed.ncbi.nlm.nih.gov//38919392/ |
| 131 | Respiratory motion prediction based on deep artificial neural networks in CyberKnife system: A comparative study | Background:\n \n \n In external beam radiotherapy, a prediction model is required to compensate for the temporal system latency that affects the accuracy of radiation dose delivery. This study focused on a thorough comparison of ... | 2023 Mar;24(3):e13854. | J Appl Clin Med Phys | Payam Samadi Miandoab; Shahyar Saramad; Saeed Setayeshi; Payam Samadi Miandoab; Payam Samadi Miandoab; Shahyar Saramad; Saeed Setayeshi | DOI:\n \n \n\n 10.1002/acm2.13854 | https://pubmed.ncbi.nlm.nih.gov//36457192/ |
| 132 | Large Language Models for Epidemiological Research via Automated Machine Learning: Case Study Using Data From the British National Child Development Study | Background:\n \n \n Large language models have had a huge impact on natural language processing (NLP) in recent years. However, their application in epidemiological research is still limited to the analysis of electronic health r... | 2023 Sep 19:11:e43638. | JMIR Med Inform | Rasmus Wibaek; Gregers Stig Andersen; Christina C Dahm; Daniel R Witte; Adam Hulman; Rasmus Wibaek; Rasmus Wibaek; Gregers Stig Andersen; Christina C Dahm; Daniel R Witte; Adam Hulman | DOI:\n \n \n\n 10.2196/43638 | https://pubmed.ncbi.nlm.nih.gov//37787655/ |
| 133 | Colorectal cancer detection with enhanced precision using a hybrid supervised and unsupervised learning approach | The current work introduces the hybrid ensemble framework for the detection and segmentation of colorectal cancer. This framework will incorporate both supervised classification and unsupervised clustering methods to present more understandable a... | 2025 Jan 25;15(1):3180. | Sci Rep | Akella S Narasimha Raju; K Venkatesh; Ranjith Kumar Gatla; Eswara Prasad Konakalla; Marwa M Eid; Nataliia Titova; Sherif S M Ghoneim; Ramy N R Ghaly; Akella S Narasimha Raju; Akella S Narasimha Raju; K Venkatesh; Ranjith Kumar Gatla; Eswara Prasa... | DOI:\n \n \n\n 10.1038/s41598-025-86590-y | https://pubmed.ncbi.nlm.nih.gov//39863646/ |
| 134 | Survival Prediction of Children Undergoing Hematopoietic Stem Cell Transplantation Using Different Machine Learning Classifiers by Performing Chi-Square Test and Hyperparameter Optimization: A Retrospective Analysis | Bone marrow transplant (BMT) is an effective surgical treatment for bone marrow-related disorders. However, several associated risk factors can impair long-term survival after BMT. Machine learning (ML) technologies have been proven useful in sur... | 2022 Sep 25:2022:9391136. | Comput Math Methods Med | Ishrak Jahan Ratul; Ummay Habiba Wani; Mirza Muntasir Nishat; Abdullah Al-Monsur; Abrar Mohammad Ar-Rafi; Fahim Faisal; Mohammad Ridwan Kabir; Ishrak Jahan Ratul; Ishrak Jahan Ratul; Ummay Habiba Wani; Mirza Muntasir Nishat; Abdullah Al-Monsur; A... | DOI:\n \n \n\n 10.1155/2022/9391136 | https://pubmed.ncbi.nlm.nih.gov//36199778/ |
| 135 | Development of a Machine Learning Based Web Application for Early Diagnosis of COVID-19 Based on Symptoms | Detecting the presence of a disease requires laboratory tests, testing kits, and devices; however, these were not always available on hand. This study proposes a new approach in disease detection using machine learning algorithms by analyzing sym... | 2022 Mar 27;12(4):821. | Diagnostics (Basel) | Charlyn Nayve Villavicencio; Julio Jerison Macrohon; Xavier Alphonse Inbaraj; Jyh-Horng Jeng; Jer-Guang Hsieh; Charlyn Nayve Villavicencio; Charlyn Nayve Villavicencio; Julio Jerison Macrohon; Xavier Alphonse Inbaraj; Jyh-Horng Jeng; Jer-Guang Hsieh | DOI:\n \n \n\n 10.3390/diagnostics12040821 | https://pubmed.ncbi.nlm.nih.gov//35453869/ |
| 136 | Development and web deployment of prediction model for pulmonary arterial pressure in chronic thromboembolic pulmonary hypertension using machine learning | Background and purpose:\n \n \n Mean pulmonary artery pressure (mPAP) is a key index for chronic thromboembolic pulmonary hypertension (CTEPH). Using machine learning, we attempted to construct an accurate prediction model for mP... | 2024 Apr 5;19(4):e0300716. | PLoS One | Takaaki Matsunaga; Atsushi Kono; Mizuho Nishio; Takahiro Yoshii; Hidetoshi Matsuo; Mai Takahashi; Takuya Takahashi; Yu Taniguchi; Hidekazu Tanaka; Kenichi Hirata; Takamichi Murakami; Takaaki Matsunaga; Takaaki Matsunaga; Atsushi Kono; Mizuho Nish... | DOI:\n \n \n\n 10.1371/journal.pone.0300716 | https://pubmed.ncbi.nlm.nih.gov//38578764/ |
| 137 | SplitAVG: A Heterogeneity-Aware Federated Deep Learning Method for Medical Imaging | Federated learning is an emerging research paradigm for enabling collaboratively training deep learning models without sharing patient data. However, the data from different institutions are usually heterogeneous across institutions, which may re... | 2022 Sep;26(9):4635-4644. | IEEE J Biomed Health Inform | Miao Zhang; Liangqiong Qu; Praveer Singh; Jayashree Kalpathy-Cramer; Daniel L Rubin; Miao Zhang; Miao Zhang; Liangqiong Qu; Praveer Singh; Jayashree Kalpathy-Cramer; Daniel L Rubin | DOI:\n \n \n\n 10.1109/JBHI.2022.3185956 | https://pubmed.ncbi.nlm.nih.gov//35749336/ |
| 138 | A machine learning study on the fatigue crack path of short crack on an α titanium alloy | In the present study, a physics-informed neural network model based on Bayesian hyperparameter optimization is proposed for the prediction of short crack growth paths. A large number of cyclic loadings at a lower amplitude were applied to an α ti... | 2023 Nov 13;381(2260):20220391. | Philos Trans A Math Phys Eng Sci | Zhengyu Shen; Guanlin Lv; Daixin Fu; Yihao Long; Zhouyu Zhang; Kai Tan; Lang Li; Qingyuan Wang; Chong Wang; Zhengyu Shen; Zhengyu Shen; Guanlin Lv; Daixin Fu; Yihao Long; Zhouyu Zhang; Kai Tan; Lang Li; Qingyuan Wang; Chong Wang | DOI:\n \n \n\n 10.1098/rsta.2022.0391 | https://pubmed.ncbi.nlm.nih.gov//37742704/ |
| 139 | Comparing machine learning screening approaches using clinical data and cytokine profiles for COVID-19 in resource-limited and resource-abundant settings | Accurate screening of COVID-19 infection status for symptomatic patients is a critical public health task. Although molecular and antigen tests now exist for COVID-19, in resource-limited settings, screening tests are often not available. Further... | 2024 Jun 28;14(1):14892. | Sci Rep | Hooman H Rashidi; Aamer Ikram; Luke T Dang; Adnan Bashir; Tanzeel Zohra; Amna Ali; Hamza Tanvir; Mohammad Mudassar; Resmi Ravindran; Nasim Akhtar; Rana I Sikandar; Mohammed Umer; Naeem Akhter; Rafi Butt; Brandon D Fennell; Imran H Khan; Hooman H ... | DOI:\n \n \n\n 10.1038/s41598-024-63707-3 | https://pubmed.ncbi.nlm.nih.gov//38937503/ |
| 140 | A New Robust Epigenetic Model for Forensic Age Prediction | Forensic DNA phenotyping refers to an emerging field of forensic sciences aimed at the prediction of externally visible characteristics of unknown sample donors directly from biological materials. The aging process significantly affects most of t... | 2020 Sep;65(5):1424-1431. | J Forensic Sci | Alberto Montesanto; Patrizia D'Aquila; Vincenzo Lagani; Ersilia Paparazzo; Silvana Geracitano; Laura Formentini; Robertina Giacconi; Maurizio Cardelli; Mauro Provinciali; Dina Bellizzi; Giuseppe Passarino; Alberto Montesanto; Alberto Montesanto; ... | DOI:\n \n \n\n 10.1111/1556-4029.14460 | https://pubmed.ncbi.nlm.nih.gov//32453457/ |
| 141 | Automated interpretation of congenital heart disease from multi-view echocardiograms | Congenital heart disease (CHD) is the most common birth defect and the leading cause of neonate death in China. Clinical diagnosis can be based on the selected 2D key-frames from five views. Limited by the availability of multi-view data, most me... | 2021 Apr:69:101942. | Med Image Anal | Jing Wang; Xiaofeng Liu; Fangyun Wang; Lin Zheng; Fengqiao Gao; Hanwen Zhang; Xin Zhang; Wanqing Xie; Binbin Wang; Jing Wang; Jing Wang; Xiaofeng Liu; Fangyun Wang; Lin Zheng; Fengqiao Gao; Hanwen Zhang; Xin Zhang; Wanqing Xie; Binbin Wang | DOI:\n \n \n\n 10.1016/j.media.2020.101942 | https://pubmed.ncbi.nlm.nih.gov//33418465/ |
| 142 | Prediction of therapy response of breast cancer patients with machine learning based on clinical data and imaging data derived from breast [18F]FDG-PET/MRI | Purpose:\n \n \n To evaluate if a machine learning prediction model based on clinical and easily assessable imaging features derived from baseline breast [18F]FDG-PET/MRI staging can predict pathologic complete response (pCR) in ... | 2024 Apr;51(5):1451-1461. | Eur J Nucl Med Mol Imaging | Kai Jannusch; Frederic Dietzel; Nils Martin Bruckmann; Janna Morawitz; Matthias Boschheidgen; Peter Minko; Ann-Kathrin Bittner; Svjetlana Mohrmann; Harald H Quick; Ken Herrmann; Lale Umutlu; Gerald Antoch; Christian Rubbert; Julian Kirchner; Juli... | DOI:\n \n \n\n 10.1007/s00259-023-06513-9 | https://pubmed.ncbi.nlm.nih.gov//38133687/ |
| 143 | Predictive model for sarcopenia in chronic kidney disease: a nomogram and machine learning approach using CHARLS data | Background:\n \n \n Sarcopenia frequently occurs as a complication among individuals with chronic kidney disease (CKD), contributing to poorer clinical outcomes. This research aimed to create and assess a predictive model for the... | 2025 Mar 12:12:1546988. | Front Med (Lausanne) | Renjie Lu; Shiyun Wang; Pinghua Chen; Fangfang Li; Pan Li; Qian Chen; Xuefei Li; Fangyu Li; Suxia Guo; Jinlin Zhang; Dan Liu; Zhijun Hu; Renjie Lu; Renjie Lu; Shiyun Wang; Pinghua Chen; Fangfang Li; Pan Li; Qian Chen; Xuefei Li; Fangyu Li; Suxia ... | DOI:\n \n \n\n 10.3389/fmed.2025.1546988 | https://pubmed.ncbi.nlm.nih.gov//40144877/ |
| 144 | Discovery of Depression-Associated Factors From a Nationwide Population-Based Survey: Epidemiological Study Using Machine Learning and Network Analysis | Background:\n \n \n In epidemiological studies, finding the best subset of factors is challenging when the number of explanatory variables is large.\n \n\n\n Objective:\n \n \n Our study had two aims.... | 2021 Jun 24;23(6):e27344. | J Med Internet Res | Sang Min Nam; Thomas A Peterson; Kyoung Yul Seo; Hyun Wook Han; Jee In Kang; Sang Min Nam; Sang Min Nam; Thomas A Peterson; Kyoung Yul Seo; Hyun Wook Han; Jee In Kang | DOI:\n \n \n\n 10.2196/27344 | https://pubmed.ncbi.nlm.nih.gov//34184998/ |
| 145 | Enhancing machine learning-based forecasting of chronic renal disease with explainable AI | Chronic renal disease (CRD) is a significant concern in the field of healthcare, highlighting the crucial need of early and accurate prediction in order to provide prompt treatments and enhance patient outcomes. This article presents an end-to-en... | 2024 Sep 26:10:e2291. | PeerJ Comput Sci | Sanjana Singamsetty; Swetha Ghanta; Sujit Biswas; Ashok Pradhan; Sanjana Singamsetty; Sanjana Singamsetty; Swetha Ghanta; Sujit Biswas; Ashok Pradhan | DOI:\n \n \n\n 10.7717/peerj-cs.2291 | https://pubmed.ncbi.nlm.nih.gov//39650439/ |
| 146 | Exploring machine learning algorithms to predict acute respiratory tract infection and identify its determinants among children under five in Sub-Saharan Africa | Background:\n \n \n The primary cause of death for children under the age of five is acute respiratory infections (ARI). Early predicting acute respiratory tract infections (ARI) and identifying their predictors using supervised ... | 2024 Nov 20:12:1388820. | Front Pediatr | Tirualem Zeleke Yehuala; Bezawit Melak Fente; Sisay Maru Wubante; Nebiyu Mekonnen Derseh; Tirualem Zeleke Yehuala; Tirualem Zeleke Yehuala; Bezawit Melak Fente; Sisay Maru Wubante; Nebiyu Mekonnen Derseh | DOI:\n \n \n\n 10.3389/fped.2024.1388820 | https://pubmed.ncbi.nlm.nih.gov//39633817/ |
| 147 | Predicting Survived Events in Nontraumatic Out-of-Hospital Cardiac Arrest: A Comparison Study on Machine Learning and Regression Models | Background:\n \n \n Prediction of early outcomes of nontraumatic out-of-hospital cardiac arrest (OHCA) by emergency physicians is inaccurate.\n \n\n\n Objective:\n \n \n Our aim was to develop and val... | 2021 Dec;61(6):683-694. | J Emerg Med | Yat Hei Lo; Yuet Chung Axel Siu; Yat Hei Lo; Yat Hei Lo; Yuet Chung Axel Siu | DOI:\n \n \n\n 10.1016/j.jemermed.2021.07.058 | https://pubmed.ncbi.nlm.nih.gov//34548227/ |
| 148 | Heart failure survival prediction using novel transfer learning based probabilistic features | Heart failure is a complex cardiovascular condition characterized by the heart's inability to pump blood effectively, leading to a cascade of physiological changes. Predicting survival in heart failure patients is crucial for optimizing patient c... | 2024 Mar 12:10:e1894. | PeerJ Comput Sci | Azam Mehmood Qadri; Muhammad Shadab Alam Hashmi; Ali Raza; Syed Ali Jafar Zaidi; Atiq Ur Rehman; Azam Mehmood Qadri; Azam Mehmood Qadri; Muhammad Shadab Alam Hashmi; Ali Raza; Syed Ali Jafar Zaidi; Atiq Ur Rehman | DOI:\n \n \n\n 10.7717/peerj-cs.1894 | https://pubmed.ncbi.nlm.nih.gov//38660216/ |
| 149 | Predicting hospitalization of pediatric asthma patients in emergency departments using machine learning | Motivation:\n \n \n The timely identification of patients for hospitalization in emergency departments (EDs) can facilitate efficient use of hospital resources. Machine learning can help the early prediction of ED disposition; ho... | 2021 Jul:151:104468. | Int J Med Inform | Marion R Sills; Mustafa Ozkaynak; Hoon Jang; Marion R Sills; Marion R Sills; Mustafa Ozkaynak; Hoon Jang | DOI:\n \n \n\n 10.1016/j.ijmedinf.2021.104468 | https://pubmed.ncbi.nlm.nih.gov//33940479/ |
| 150 | Coverage-Based Designs Improve Sample Mining and Hyperparameter Optimization | Sampling one or more effective solutions from large search spaces is a recurring idea in machine learning (ML), and sequential optimization has become a popular solution. Typical examples include data summarization, sample mining for predictive m... | 2021 Mar;32(3):1241-1253. | IEEE Trans Neural Netw Learn Syst | Gowtham Muniraju; Bhavya Kailkhura; Jayaraman J Thiagarajan; Peer-Timo Bremer; Cihan Tepedelenlioglu; Andreas Spanias; Gowtham Muniraju; Gowtham Muniraju; Bhavya Kailkhura; Jayaraman J Thiagarajan; Peer-Timo Bremer; Cihan Tepedelenlioglu; Andreas... | DOI:\n \n \n\n 10.1109/TNNLS.2020.2982936 | https://pubmed.ncbi.nlm.nih.gov//32305942/ |
| 151 | A Unified Framework for Automatic Distributed Active Learning | We propose a novel unified frameork for automated distributed active learning (AutoDAL) to address multiple challenging problems in active learning such as limited labeled data, imbalanced datasets, automatic hyperparameter selection as well as s... | 2022 Dec;44(12):9774-9786. | IEEE Trans Pattern Anal Mach Intell | Xu Chen; Brett Wujek; Xu Chen; Xu Chen; Brett Wujek | DOI:\n \n \n\n 10.1109/TPAMI.2021.3129793 | https://pubmed.ncbi.nlm.nih.gov//34813465/ |
| 152 | Modelling 30-day hospital readmission after discharge for COPD patients based on electronic health records | Chronic Obstructive Pulmonary Disease (COPD) is the third most common chronic disease in China with frequent exacerbations, resulting in increased hospitalization and readmission rate. COPD readmission within 30 days after discharge is an importa... | 2023 Apr 10;33(1):16. | NPJ Prim Care Respir Med | Meng Li; Kun Cheng; Keisun Ku; Junlei Li; Hao Hu; Carolina Oi Lam Ung; Meng Li; Meng Li; Kun Cheng; Keisun Ku; Junlei Li; Hao Hu; Carolina Oi Lam Ung | DOI:\n \n \n\n 10.1038/s41533-023-00339-6 | https://pubmed.ncbi.nlm.nih.gov//37037836/ |
| 153 | Searching to Exploit Memorization Effect in Deep Learning With Noisy Labels | Sample selection approaches are popular in robust learning from noisy labels. However, how to control the selection process properly so that deep networks can benefit from the memorization effect is a hard problem. In this paper, motivated by the... | 2024 Dec;46(12):7833-7849. | IEEE Trans Pattern Anal Mach Intell | Hansi Yang; Quanming Yao; Bo Han; James T Kwok; Hansi Yang; Hansi Yang; Quanming Yao; Bo Han; James T Kwok | DOI:\n \n \n\n 10.1109/TPAMI.2024.3394552 | https://pubmed.ncbi.nlm.nih.gov//38683712/ |
| 154 | Development of advanced machine learning models for analysis of plutonium surrogate optical emission spectra | This work investigates and applies machine learning paradigms seldom seen in analytical spectroscopy for quantification of gallium in cerium matrices via processing of laser-plasma spectra. Ensemble regressions, support vector machine regressions... | 2022 Mar 1;61(7):D30-D38. | Appl Opt | Ashwin P Rao; Phillip R Jenkins; John D Auxier; Michael B Shattan; Anil K Patnaik; Ashwin P Rao; Ashwin P Rao; Phillip R Jenkins; John D Auxier; Michael B Shattan; Anil K Patnaik | DOI:\n \n \n\n 10.1364/AO.444093 | https://pubmed.ncbi.nlm.nih.gov//35297826/ |
| 155 | Risk Prediction of Liver Injury in Pediatric Tuberculosis Treatment: Development of an Automated Machine Learning Model | Purpose:\n \n \n Drug-induced liver injury (DILI) is one of the most common and serious adverse drug reactions related to first-line anti-tuberculosis drugs in pediatric tuberculosis patients. This study aims to develop an automa... | 2025 Jan 13:19:239-250. | Drug Des Devel Ther | Ying Zeng; Hong Lu; Sen Li; Qun-Zhi Shi; Lin Liu; Yong-Qing Gong; Pan Yan; Ying Zeng; Ying Zeng; Hong Lu; Sen Li; Qun-Zhi Shi; Lin Liu; Yong-Qing Gong; Pan Yan | DOI:\n \n \n\n 10.2147/DDDT.S495555 | https://pubmed.ncbi.nlm.nih.gov//39830784/ |
| 156 | Beyond benchmarking and towards predictive models of dataset-specific single-cell RNA-seq pipeline performance | Background:\n \n \n The advent of single-cell RNA-sequencing (scRNA-seq) has driven significant computational methods development for all steps in the scRNA-seq data analysis pipeline, including filtering, normalization, and clus... | 2024 Jun 17;25(1):159. | Genome Biol | Cindy Fang; Alina Selega; Kieran R Campbell; Cindy Fang; Cindy Fang; Alina Selega; Kieran R Campbell | DOI:\n \n \n\n 10.1186/s13059-024-03304-9 | https://pubmed.ncbi.nlm.nih.gov//38886757/ |
| 157 | Correction of the travel time estimation for ambulances of the red cross Tijuana using machine learning | This paper addresses the problem of estimating the response time to a medical emergency, specifically from the Red Cross of Tijuana (RCT), which provides most of the emergency medical services (EMS) in the city of Tijuana, Mexico. For institution... | 2021 Oct:137:104798. | Comput Biol Med | Noelia Torres; Leonardo Trujillo; Yazmin Maldonado; Carlos Vera; Noelia Torres; Noelia Torres; Leonardo Trujillo; Yazmin Maldonado; Carlos Vera | DOI:\n \n \n\n 10.1016/j.compbiomed.2021.104798 | https://pubmed.ncbi.nlm.nih.gov//34482200/ |
| 158 | A data-driven interpretable ensemble framework based on tree models for forecasting the occurrence of COVID-19 in the USA | This prevalence of coronavirus disease 2019 (COVID-19) has become one of the most serious public health crises. Tree-based machine learning methods, with the advantages of high efficiency, and strong interpretability, have been widely used in pre... | 2023 Jan;30(5):13648-13659. | Environ Sci Pollut Res Int | Hu-Li Zheng; Shu-Yi An; Bao-Jun Qiao; Peng Guan; De-Sheng Huang; Wei Wu; Hu-Li Zheng; Hu-Li Zheng; Shu-Yi An; Bao-Jun Qiao; Peng Guan; De-Sheng Huang; Wei Wu | DOI:\n \n \n\n 10.1007/s11356-022-23132-3 | https://pubmed.ncbi.nlm.nih.gov//36131178/ |
| 159 | A multi-dimensional student performance prediction model (MSPP): An advanced framework for accurate academic classification and analysis | Forecasting student performance with precision in the educational space is paramount for creating tailor-made interventions capable to boost learning effectiveness. It means most of the traditional student performance prediction models have diffi... | 2024 Dec 30:14:103148. | MethodsX | V Balachandar; K Venkatesh; V Balachandar; V Balachandar; K Venkatesh | DOI:\n \n \n\n 10.1016/j.mex.2024.103148 | https://pubmed.ncbi.nlm.nih.gov//39866196/ |
| 160 | Generative Adversarial Network Image Synthesis Method for Skin Lesion Generation and Classification | Background:\n \n \n One of the common limitations in the treatment of cancer is in the early detection of this disease. The customary medical practice of cancer examination is a visual examination by the dermatologist followed by... | 2021 Oct 20;11(4):237-252. | J Med Signals Sens | Freedom Mutepfe; Behnam Kiani Kalejahi; Saeed Meshgini; Sebelan Danishvar; Freedom Mutepfe; Freedom Mutepfe; Behnam Kiani Kalejahi; Saeed Meshgini; Sebelan Danishvar | DOI:\n \n \n\n 10.4103/jmss.JMSS\_53\_20 | https://pubmed.ncbi.nlm.nih.gov//34820296/ |
| 161 | Enhancing mortality prediction in patients with spontaneous intracerebral hemorrhage: Radiomics and supervised machine learning on non-contrast computed tomography | Purpose:\n \n \n This study aims to develop a Radiomics-based Supervised Machine-Learning model to predict mortality in patients with spontaneous intracerebral hemorrhage (sICH).\n \n\n\n Methods:\n \n \n ... | 2024 Dec 1:13:100618. | Eur J Radiol Open | Antonio López-Rueda; María-Ángeles Rodríguez-Sánchez; Elena Serrano; Javier Moreno; Alejandro Rodríguez; Laura Llull; Sergi Amaro; Laura Oleaga; Antonio López-Rueda; Antonio López-Rueda; María-Ángeles Rodríguez-Sánchez; Elena Serrano; Javier More... | DOI:\n \n \n\n 10.1016/j.ejro.2024.100618 | https://pubmed.ncbi.nlm.nih.gov//39687913/ |
| 162 | A revolutionary acute subdural hematoma detection based on two-tiered artificial intelligence model | Background:\n \n \n The article was planned to make the first evaluation in terms of acute subdural hemorrhages, thinking that it can help in appropriate pathologies by tomography interpretation with the artificial intelligence (... | 2023 Aug;29(8):858-871. | Ulus Travma Acil Cerrahi Derg | İsmail Kaya; Tuğrul Hakan Gençtürk; Fidan Kaya Gülağız; İsmail Kaya; İsmail Kaya; Tuğrul Hakan Gençtürk; Fidan Kaya Gülağız | DOI:\n \n \n\n 10.14744/tjtes.2023.76756 | https://pubmed.ncbi.nlm.nih.gov//37563894/ |
| 163 | Spatial predictions of groundwater potential using automated machine learning (AutoML): a comparative study of feature selection and training sample size in Qinghai Province, China | Predicting groundwater potential is crucial for identifying the spatial distribution of groundwater in a region. It serves as an essential guide for the development, utilization, and protection of groundwater resources. Previous studies have prim... | 2024 Jan;31(1):1127-1145. | Environ Sci Pollut Res Int | Zitao Wang; Jianping Wang; Mengling Li; Zitao Wang; Zitao Wang; Jianping Wang; Mengling Li | DOI:\n \n \n\n 10.1007/s11356-023-31262-5 | https://pubmed.ncbi.nlm.nih.gov//38038910/ |
| 164 | Intrinsic radiomic expression patterns after 20 Gy demonstrate early metabolic response of oropharyngeal cancers | Purpose:\n \n \n This study investigated the prognostic potential of intra-treatment PET radiomics data in patients undergoing definitive (chemo) radiation therapy for oropharyngeal cancer (OPC) on a prospective clinical trial. W... | 2021 Jul;48(7):3767-3777. | Med Phys | Kyle J Lafata; Yushi Chang; Chunhao Wang; Yvonne M Mowery; Irina Vergalasova; Donna Niedzwiecki; David S Yoo; Jian-Guo Liu; David M Brizel; Fang-Fang Yin; Kyle J Lafata; Kyle J Lafata; Yushi Chang; Chunhao Wang; Yvonne M Mowery; Irina Vergalasova... | DOI:\n \n \n\n 10.1002/mp.14926 | https://pubmed.ncbi.nlm.nih.gov//33959972/ |
| 165 | Prediction of lead (Pb) adsorption on attapulgite clay using the feasibility of data intelligence models | This study investigates the performance of support vector machine (SVM), multivariate adaptive regression spline (MARS), and random forest (RF) models for predicting the lead (Pb) adsorption by attapulgite clay. Models are constructed using batch... | 2021 Jun;28(24):31670-31688. | Environ Sci Pollut Res Int | Suraj Kumar Bhagat; Mariapparaj Paramasivan; Mustafa Al-Mukhtar; Tiyasha Tiyasha; Konstantina Pyrgaki; Tran Minh Tung; Zaher Mundher Yaseen; Suraj Kumar Bhagat; Suraj Kumar Bhagat; Mariapparaj Paramasivan; Mustafa Al-Mukhtar; Tiyasha Tiyasha; Kon... | DOI:\n \n \n\n 10.1007/s11356-021-12836-7 | https://pubmed.ncbi.nlm.nih.gov//33611749/ |
| 166 | Two-Year Event-Free Survival Prediction in DLBCL Patients Based on In Vivo Radiomics and Clinical Parameters | Purpose:\n \n \n For the identification of high-risk patients in diffuse large B-cell lymphoma (DLBCL), we investigated the prognostic significance of in vivo radiomics derived from baseline [18F]FDG PET/CT and clinical parameter... | 2022 Jun 8:12:820136. | Front Oncol | Zsombor Ritter; László Papp; Katalin Zámbó; Zoltán Tóth; Dániel Dezső; Dániel Sándor Veres; Domokos Máthé; Ferenc Budán; Éva Karádi; Anett Balikó; László Pajor; Árpád Szomor; Erzsébet Schmidt; Hussain Alizadeh; Zsombor Ritter; Zsombor Ritter; Lás... | DOI:\n \n \n\n 10.3389/fonc.2022.820136 | https://pubmed.ncbi.nlm.nih.gov//35756658/ |
| 167 | Machine learning methods applied to audit of surgical margins after curative surgery for facial (non-melanoma) skin cancer | We aimed to build a model to predict positive margin status after curative excision of facial non-melanoma skin cancer based on known risk factors that contribute to the complexity of the case mix. A pathology output of consecutive histology repo... | 2023 Jan;61(1):94-100. | Br J Oral Maxillofac Surg | David Tighe; Kemal Tekeli; Tara Gouk; Jennifer Smith; Michael Ho; Andrew Moody; Stephen Walsh; Simon Provost; Alex Freitas; David Tighe; David Tighe; Kemal Tekeli; Tara Gouk; Jennifer Smith; Michael Ho; Andrew Moody; Stephen Walsh; Simon Provost;... | DOI:\n \n \n\n 10.1016/j.bjoms.2022.11.280 | https://pubmed.ncbi.nlm.nih.gov//36631333/ |
| 168 | Democratizing AI: non-expert design of prediction tasks | Non-experts have long made important contributions to machine learning (ML) by contributing training data, and recent work has shown that non-experts can also help with feature engineering by suggesting novel predictive features. However, non-exp... | 2020 Sep 7:6:e296. | PeerJ Comput Sci | James P Bagrow; James P Bagrow; James P Bagrow | DOI:\n \n \n\n 10.7717/peerj-cs.296 | https://pubmed.ncbi.nlm.nih.gov//33816947/ |
| 169 | Fast deep learning reconstruction techniques for preclinical magnetic resonance fingerprinting | We propose a deep learning (DL) model and a hyperparameter optimization strategy to reconstruct T1 and T2 maps acquired with the magnetic resonance fingerprinting (MRF) methodology. We applied two different MRF sequence routines to acquire images... | 2024 Jan;37(1):e5028. | NMR Biomed | Raffaella Fiamma Cabini; Leonardo Barzaghi; Davide Cicolari; Paolo Arosio; Stefano Carrazza; Silvia Figini; Marta Filibian; Andrea Gazzano; Rolf Krause; Manuel Mariani; Marco Peviani; Anna Pichiecchio; Diego Ulisse Pizzagalli; Alessandro Lascialf... | DOI:\n \n \n\n 10.1002/nbm.5028 | https://pubmed.ncbi.nlm.nih.gov//37669779/ |
| 170 | Federated semi-supervised learning for COVID region segmentation in chest CT using multi-national data from China, Italy, Japan | The recent outbreak of Coronavirus Disease 2019 (COVID-19) has led to urgent needs for reliable diagnosis and management of SARS-CoV-2 infection. The current guideline is using RT-PCR for testing. As a complimentary tool with diagnostic imaging, ... | 2021 May:70:101992. | Med Image Anal | Dong Yang; Ziyue Xu; Wenqi Li; Andriy Myronenko; Holger R Roth; Stephanie Harmon; Sheng Xu; Baris Turkbey; Evrim Turkbey; Xiaosong Wang; Wentao Zhu; Gianpaolo Carrafiello; Francesca Patella; Maurizio Cariati; Hirofumi Obinata; Hitoshi Mori; Kaku ... | DOI:\n \n \n\n 10.1016/j.media.2021.101992 | https://pubmed.ncbi.nlm.nih.gov//33601166/ |
| 171 | Aviation Turbine Fuel Thermal Conductivity: A Predictive Approach Using Entropy Scaling-Guided Machine Learning with Experimental Validation | Although typical aircraft fuel thermal management analysis relies upon temperature-dependent thermodynamic and transport properties of aviation turbine fuel, the variation in properties associated with compositional variation in fuels and the sub... | 2021 Oct 21;6(43):28579-28586. | ACS Omega | William Anthony Malatesta; Bao Yang; William Anthony Malatesta; William Anthony Malatesta; Bao Yang | DOI:\n \n \n\n 10.1021/acsomega.1c02934 | https://pubmed.ncbi.nlm.nih.gov//34746553/ |
| 172 | Risk prediction of integrated traditional Chinese and western medicine for diabetes retinopathy based on optimized gradient boosting classifier model | In order to take full advantage of traditional Chinese medicine (TCM) and western medicine, combined with machine learning technology, to study the risk factors and better risk prediction model of diabetic retinopathy (DR), and provide basis for ... | 2024 Dec 20;103(51):e40896. | Medicine (Baltimore) | Li Xiao; Lixuan Tang; Wenxuan Kuang; Yijing Yang; Ying Deng; Jing Lu; Qinghua Peng; Junfeng Yan; Li Xiao; Li Xiao; Lixuan Tang; Wenxuan Kuang; Yijing Yang; Ying Deng; Jing Lu; Qinghua Peng; Junfeng Yan | DOI:\n \n \n\n 10.1097/MD.0000000000040896 | https://pubmed.ncbi.nlm.nih.gov//39705459/ |
| 173 | Self-supervised learning for classifying paranasal anomalies in the maxillary sinus | Purpose:\n \n \n Paranasal anomalies, frequently identified in routine radiological screenings, exhibit diverse morphological characteristics. Due to the diversity of anomalies, supervised learning methods require large labelled ... | 2024 Sep;19(9):1713-1721. | Int J Comput Assist Radiol Surg | Debayan Bhattacharya; Finn Behrendt; Benjamin Tobias Becker; Lennart Maack; Dirk Beyersdorff; Elina Petersen; Marvin Petersen; Bastian Cheng; Dennis Eggert; Christian Betz; Anna Sophie Hoffmann; Alexander Schlaefer; Debayan Bhattacharya; Debayan ... | DOI:\n \n \n\n 10.1007/s11548-024-03172-5 | https://pubmed.ncbi.nlm.nih.gov//38850438/ |
| 174 | On Robustness of Neural Architecture Search Under Label Noise | Neural architecture search (NAS), which aims at automatically seeking proper neural architectures given a specific task, has attracted extensive attention recently in supervised learning applications. In most real-world situations, the class labe... | 2020 Feb 11:3:2. | Front Big Data | Yi-Wei Chen; Qingquan Song; Xi Liu; P S Sastry; Xia Hu; Yi-Wei Chen; Yi-Wei Chen; Qingquan Song; Xi Liu; P S Sastry; Xia Hu | DOI:\n \n \n\n 10.3389/fdata.2020.00002 | https://pubmed.ncbi.nlm.nih.gov//33693377/ |
| 175 | Prediction of microvascular obstruction from angio-based microvascular resistance and available clinical data in percutaneous coronary intervention: an explainable machine learning model | Angio-based microvascular resistance (AMR) as a potential alternative to the index of microcirculatory resistance (IMR) and its relationship with microvascular obstruction (MVO) and other cardiac magnetic resonance (CMR) parameters still lacks co... | 2025 Jan 24;15(1):3045. | Sci Rep | Zhe Zhang; Yang Dai; Peng Xue; Xue Bao; Xinbo Bai; Shiyang Qiao; Yuan Gao; Xuemei Guo; Yanan Xue; Qing Dai; Biao Xu; Lina Kang; Zhe Zhang; Zhe Zhang; Yang Dai; Peng Xue; Xue Bao; Xinbo Bai; Shiyang Qiao; Yuan Gao; Xuemei Guo; Yanan Xue; Qing Dai;... | DOI:\n \n \n\n 10.1038/s41598-025-87828-5 | https://pubmed.ncbi.nlm.nih.gov//39856375/ |
| 176 | Deep neural network-based prediction of tsunami wave attenuation by mangrove forests | The goal of this research is to develop a model employing deep neural networks (DNNs) to predict the effectiveness of mangrove forests in attenuating the impact of tsunami waves. The dataset for the DNN model is obtained by simulating tsunami wav... | 2024 Jun 11:13:102791. | MethodsX | Didit Adytia; Dede Tarwidi; Deni Saepudin; Semeidi Husrin; Abdul Rahman Mohd Kasim; Mohd Fakhizan Romlie; Dafrizal Samsudin; Didit Adytia; Didit Adytia; Dede Tarwidi; Deni Saepudin; Semeidi Husrin; Abdul Rahman Mohd Kasim; Mohd Fakhizan Romlie; D... | DOI:\n \n \n\n 10.1016/j.mex.2024.102791 | https://pubmed.ncbi.nlm.nih.gov//38975289/ |
| 177 | An Empirical Evaluation of a Novel Ensemble Deep Neural Network Model and Explainable AI for Accurate Segmentation and Classification of Ovarian Tumors Using CT Images | Ovarian cancer is one of the leading causes of death worldwide among the female population. Early diagnosis is crucial for patient treatment. In this work, our main objective is to accurately detect and classify ovarian cancer. To achieve this, t... | 2024 Mar 4;14(5):543. | Diagnostics (Basel) | Ashwini Kodipalli; Steven L Fernandes; Santosh Dasar; Ashwini Kodipalli; Ashwini Kodipalli; Steven L Fernandes; Santosh Dasar | DOI:\n \n \n\n 10.3390/diagnostics14050543 | https://pubmed.ncbi.nlm.nih.gov//38473015/ |
| 178 | ABCModeller: an automatic data mining tool based on a consistent voting method with a user-friendly graphical interface | In order to extract useful information from a huge amount of biological data nowadays, simple and convenient tools are urgently needed for data analysis and modeling. In this paper, an automatic data mining tool, termed as ABCModeller (Automatic ... | 2021 Jul 20;22(4):bbaa247. | Brief Bioinform | Pengyi Zhang; Jiangpeng Wu; Honglin Zhai; Shuyan Li; Pengyi Zhang; Pengyi Zhang; Jiangpeng Wu; Honglin Zhai; Shuyan Li | DOI:\n \n \n\n 10.1093/bib/bbaa247 | https://pubmed.ncbi.nlm.nih.gov//33057581/ |
| 179 | Quantitative image features from radiomic biopsy differentiate oncocytoma from chromophobe renal cell carcinoma | Purpose: To differentiate oncocytoma and chromophobe renal cell carcinoma (RCC) using radiomics features computed from spherical samples of image regions of interest, "radiomic biopsies" (RBs). Approach: In a retrospective cohort study of 102 CT ... | 2021 Sep;8(5):054501. | J Med Imaging (Bellingham) | Akshay Jaggi; Domenico Mastrodicasa; Gregory W Charville; R Brooke Jeffrey Jr; Sandy Napel; Bhavik Patel; Akshay Jaggi; Akshay Jaggi; Domenico Mastrodicasa; Gregory W Charville; R Brooke Jeffrey Jr; Sandy Napel; Bhavik Patel | DOI:\n \n \n\n 10.1117/1.JMI.8.5.054501 | https://pubmed.ncbi.nlm.nih.gov//34514033/ |
| 180 | Neonatal Heart and Lung Sound Quality Assessment for Robust Heart and Breathing Rate Estimation for Telehealth Applications | With advances in digital stethoscopes, internet of things, signal processing and machine learning, chest sounds can be easily collected and transmitted to the cloud for remote monitoring and diagnosis. However, low quality of recordings complicat... | 2021 Dec;25(12):4255-4266. | IEEE J Biomed Health Inform | Ethan Grooby; Jinyuan He; Julie Kiewsky; Davood Fattahi; Lindsay Zhou; Arrabella King; Ashwin Ramanathan; Atul Malhotra; Guy A Dumont; Faezeh Marzbanrad; Ethan Grooby; Ethan Grooby; Jinyuan He; Julie Kiewsky; Davood Fattahi; Lindsay Zhou; Arrabel... | DOI:\n \n \n\n 10.1109/JBHI.2020.3047602 | https://pubmed.ncbi.nlm.nih.gov//33370240/ |
| 181 | AI-based disease category prediction model using symptoms from low-resource Ethiopian language: Afaan Oromo text | Automated disease diagnosis and prediction, powered by AI, play a crucial role in enabling medical professionals to deliver effective care to patients. While such predictive tools have been extensively explored in resource-rich languages like Eng... | 2024 May 16;14(1):11233. | Sci Rep | Etana Fikadu Dinsa; Mrinal Das; Teklu Urgessa Abebe; Etana Fikadu Dinsa; Etana Fikadu Dinsa; Mrinal Das; Teklu Urgessa Abebe | DOI:\n \n \n\n 10.1038/s41598-024-62278-7 | https://pubmed.ncbi.nlm.nih.gov//38755269/ |
| 182 | Optimizing PGRs for in vitro shoot proliferation of pomegranate with bayesian-tuned ensemble stacking regression and NSGA-II: a comparative evaluation of machine learning models | Background:\n \n \n The process of optimizing in vitro shoot proliferation is a complicated task, as it is influenced by interactions of many factors as well as genotype. This study investigated the role of various concentrations... | 2024 May 31;20(1):82. | Plant Methods | Saeedeh Zarbakhsh; Ali Reza Shahsavar; Mohammad Soltani; Saeedeh Zarbakhsh; Saeedeh Zarbakhsh; Ali Reza Shahsavar; Mohammad Soltani | DOI:\n \n \n\n 10.1186/s13007-024-01211-5 | https://pubmed.ncbi.nlm.nih.gov//38822411/ |
| 183 | A deep learning-based interpretable decision tool for predicting high risk of chemotherapy-induced nausea and vomiting in cancer patients prescribed highly emetogenic chemotherapy | Objective:\n \n \n This study aims to develop a risk prediction model for chemotherapy-induced nausea and vomiting (CINV) in cancer patients receiving highly emetogenic chemotherapy (HEC) and identify the variables that have the ... | 2023 Sep;12(17):18306-18316. | Cancer Med | Jingyue Zhang; Xudong Cui; Chong Yang; Diansheng Zhong; Yinjuan Sun; Xiaoxiong Yue; Gaoshuang Lan; Linlin Zhang; Liangfu Lu; Hengjie Yuan; Jingyue Zhang; Jingyue Zhang; Xudong Cui; Chong Yang; Diansheng Zhong; Yinjuan Sun; Xiaoxiong Yue; Gaoshuan... | DOI:\n \n \n\n 10.1002/cam4.6428 | https://pubmed.ncbi.nlm.nih.gov//37609808/ |
| 184 | Epidemiology and risk factors of Clonorchis sinensis infection in the mountainous areas of Longsheng County, Guangxi: insights from automated machine learning | Clonorchis sinensis (C. sinensis) is mainly prevalent in Northeast and South China, with Guangxi being the most severely affected region. This study aimed to evaluate the prevalence and identify the risk factors of C. sinensis infection in Longsh... | 2025 Mar 5;124(3):26. | Parasitol Res | Xiaowen Li; Yu Chen; Guoyang Huang; Xuerong Sun; Gang Mo; Xiaohong Peng; Xiaowen Li; Xiaowen Li; Yu Chen; Guoyang Huang; Xuerong Sun; Gang Mo; Xiaohong Peng | DOI:\n \n \n\n 10.1007/s00436-025-08470-8 | https://pubmed.ncbi.nlm.nih.gov//40038107/ |
| 185 | iCpG-Pos: an accurate computational approach for identification of CpG sites using positional features on single-cell whole genome sequence data | Motivation:\n \n \n The investigation of DNA methylation can shed light on the processes underlying human well-being and help determine overall human health. However, insufficient coverage makes it challenging to implement single... | 2023 Aug 1;39(8):btad474. | Bioinformatics | Sehi Park; Mobeen Ur Rehman; Farman Ullah; Hilal Tayara; Kil To Chong; Sehi Park; Sehi Park; Mobeen Ur Rehman; Farman Ullah; Hilal Tayara; Kil To Chong | DOI:\n \n \n\n 10.1093/bioinformatics/btad474 | https://pubmed.ncbi.nlm.nih.gov//37555812/ |
| 186 | Siamese model for collateral score prediction from computed tomography angiography images in acute ischemic stroke | Introduction:\n \n \n Imaging biomarkers, such as the collateral score as determined from Computed Tomography Angiography (CTA) images, play a role in treatment decision making for acute stroke patients. In this manuscript, we pr... | 2024 Jan 11:2:1239703. | Front Neuroimaging | Valerio Fortunati; Jiahang Su; Lennard Wolff; Pieter-Jan van Doormaal; Jeanette Hofmeijer; Jasper Martens; Reinoud P H Bokkers; Wim H van Zwam; Aad van der Lugt; Theo van Walsum; Valerio Fortunati; Valerio Fortunati; Jiahang Su; Lennard Wolff; Pi... | DOI:\n \n \n\n 10.3389/fnimg.2023.1239703 | https://pubmed.ncbi.nlm.nih.gov//38274412/ |
| 187 | Design of optimal Elman Recurrent Neural Network based prediction approach for biofuel production | Renewable sources like biofuels have gained significant attention to meet the rising demands of energy supply. Biofuels find useful in several domains of energy generation such as electricity, power, or transportation. Due to the environmental be... | 2023 May 26;13(1):8565. | Sci Rep | N Paramesh Kumar; S Vijayabaskar; L Murali; Krishnaraj Ramaswamy; N Paramesh Kumar; N Paramesh Kumar; S Vijayabaskar; L Murali; Krishnaraj Ramaswamy | DOI:\n \n \n\n 10.1038/s41598-023-34764-x | https://pubmed.ncbi.nlm.nih.gov//37237033/ |
| 188 | FIT calculator: a multi-risk prediction framework for medical outcomes using cardiorespiratory fitness data | Accurately predicting patients' risk for specific medical outcomes is paramount for effective healthcare management and personalized medicine. While a substantial body of literature addresses the prediction of diverse medical conditions, existing... | 2024 Apr 16;14(1):8745. | Sci Rep | Radwa Elshawi; Sherif Sakr; Mouaz H Al-Mallah; Steven J Keteyian; Clinton A Brawner; Jonathan K Ehrman; Radwa Elshawi; Radwa Elshawi; Sherif Sakr; Mouaz H Al-Mallah; Steven J Keteyian; Clinton A Brawner; Jonathan K Ehrman | DOI:\n \n \n\n 10.1038/s41598-024-59401-z | https://pubmed.ncbi.nlm.nih.gov//38627439/ |
| 189 | Application of deep learning upon spinal radiographs to predict progression in adolescent idiopathic scoliosis at first clinic visit | Background:\n \n \n Prediction of curve progression risk in adolescent idiopathic scoliosis (AIS) remains elusive. Prior studies have revealed the potential for three-dimensional (3D) morphological parameters to prognosticate pro... | 2021 Nov 29:42:101220. | EClinicalMedicine | Hongfei Wang; Teng Zhang; Kenneth Man-Chee Cheung; Graham Ka-Hon Shea; Hongfei Wang; Hongfei Wang; Teng Zhang; Kenneth Man-Chee Cheung; Graham Ka-Hon Shea | DOI:\n \n \n\n 10.1016/j.eclinm.2021.101220 | https://pubmed.ncbi.nlm.nih.gov//34901796/ |
| 190 | Efficient Pneumonia Detection in Chest Xray Images Using Deep Transfer Learning | Pneumonia causes the death of around 700,000 children every year and affects 7% of the global population. Chest X-rays are primarily used for the diagnosis of this disease. However, even for a trained radiologist, it is a challenging task to exam... | 2020 Jun 19;10(6):417. | Diagnostics (Basel) | Mohammad Farukh Hashmi; Satyarth Katiyar; Avinash G Keskar; Neeraj Dhanraj Bokde; Zong Woo Geem; Mohammad Farukh Hashmi; Mohammad Farukh Hashmi; Satyarth Katiyar; Avinash G Keskar; Neeraj Dhanraj Bokde; Zong Woo Geem | DOI:\n \n \n\n 10.3390/diagnostics10060417 | https://pubmed.ncbi.nlm.nih.gov//32575475/ |
| 191 | Advancing Interstitial Cystitis/Bladder Pain Syndrome (IC/BPS) Diagnosis: A Comparative Analysis of Machine Learning Methodologies | Background/Objectives. This study aimed to improve machine learning models for diagnosing interstitial cystitis/bladder pain syndrome (IC/BPS) by comparing classical machine learning methods with newer AutoML approaches, utilizing biomarker data ... | 2024 Dec 5;14(23):2734. | Diagnostics (Basel) | Joseph J Janicki; Bernadette M M Zwaans; Sarah N Bartolone; Elijah P Ward; Michael B Chancellor; Joseph J Janicki; Joseph J Janicki; Bernadette M M Zwaans; Sarah N Bartolone; Elijah P Ward; Michael B Chancellor | DOI:\n \n \n\n 10.3390/diagnostics14232734 | https://pubmed.ncbi.nlm.nih.gov//39682641/ |
| 192 | The application of machine learning for treatment selection of unruptured brain arteriovenous malformations: A secondary analysis of the ARUBA trial data | Objective:\n \n \n To build a supervised machine learning (ML) model that selects the best first-line treatment strategy for unruptured bAVMs.\n \n\n\n Methods:\n \n \n A Randomized Trial of Unrupture... | 2025 Feb:249:108681. | Clin Neurol Neurosurg | Tejas Venkataram; Shreyas Kashyap; Mandara M Harikar; Francesco Inserra; Fabio Barone; Mario Travali; Valeriox Da Ros; Giuseppe E Umana; Oluseye A Ogunbayo; Benjamin Aribisala; Tejas Venkataram; Tejas Venkataram; Shreyas Kashyap; Mandara M Harika... | DOI:\n \n \n\n 10.1016/j.clineuro.2024.108681 | https://pubmed.ncbi.nlm.nih.gov//39673942/ |
| 193 | Treatment effect analysis of the Frailty Care Bundle (FCB) in a cohort of patients in acute care settings | Purpose:\n \n \n The aim of this study is to explore the feasibility of using machine learning approaches to objectively differentiate the mobilization patterns, measured via accelerometer sensors, of patients pre- and post-inter... | 2024 Sep 10;36(1):187. | Aging Clin Exp Res | Colum Crowe; Corina Naughton; Marguerite de Foubert; Helen Cummins; Ruth McCullagh; Dawn A Skelton; Darren Dahly; Brendan Palmer; Brendan O'Flynn; Salvatore Tedesco; Colum Crowe; Colum Crowe; Corina Naughton; Marguerite de Foubert; Helen Cummins;... | DOI:\n \n \n\n 10.1007/s40520-024-02840-5 | https://pubmed.ncbi.nlm.nih.gov//39254891/ |
| 194 | Combined Input Deep Learning Pipeline for Embryo Selection for In Vitro Fertilization Using Light Microscopic Images and Additional Features | The current process of embryo selection in in vitro fertilization is based on morphological criteria; embryos are manually evaluated by embryologists under subjective assessment. In this study, a deep learning-based pipeline was developed to clas... | 2025 Jan 7;11(1):13. | J Imaging | Krittapat Onthuam; Norrawee Charnpinyo; Kornrapee Suthicharoenpanich; Supphaset Engphaiboon; Punnarai Siricharoen; Ronnapee Chaichaowarat; Chanakarn Suebthawinkul; Krittapat Onthuam; Krittapat Onthuam; Norrawee Charnpinyo; Kornrapee Suthicharoenp... | DOI:\n \n \n\n 10.3390/jimaging11010013 | https://pubmed.ncbi.nlm.nih.gov//39852326/ |
| 195 | Two-Year Hypertension Incidence Risk Prediction in Populations in the Desert Regions of Northwest China: Prospective Cohort Study | Background:\n \n \n Hypertension is a major global health issue and a significant modifiable risk factor for cardiovascular diseases, contributing to a substantial socioeconomic burden due to its high prevalence. In China, partic... | 2025 Mar 12:27:e68442. | J Med Internet Res | Yinlin Cheng; Kuiying Gu; Weidong Ji; Zhensheng Hu; Yining Yang; Yi Zhou; Yinlin Cheng; Yinlin Cheng; Kuiying Gu; Weidong Ji; Zhensheng Hu; Yining Yang; Yi Zhou | DOI:\n \n \n\n 10.2196/68442 | https://pubmed.ncbi.nlm.nih.gov//40072485/ |
| 196 | Predicting adverse birth outcome among childbearing women in Sub-Saharan Africa: employing innovative machine learning techniques | Background:\n \n \n Adverse birth outcomes, including preterm birth, low birth weight, and stillbirth, remain a major global health challenge, particularly in developing regions. Understanding the possible risk factors is crucial... | 2024 Jul 29;24(1):2029. | BMC Public Health | Habtamu Setegn Ngusie; Shegaw Anagaw Mengiste; Alemu Birara Zemariam; Bogale Molla; Getanew Aschalew Tesfa; Binyam Tariku Seboka; Tilahun Dessie Alene; Jing Sun; Habtamu Setegn Ngusie; Habtamu Setegn Ngusie; Shegaw Anagaw Mengiste; Alemu Birara Z... | DOI:\n \n \n\n 10.1186/s12889-024-19566-8 | https://pubmed.ncbi.nlm.nih.gov//39075434/ |
| 197 | Optimized neural network-based model to predict the shear strength of trapezoidal-corrugated steel webs | Beam-like members use corrugated webs to increase their shear strength, stability, and efficiency. The corrugation positively affects the members' structural characteristics, especially those governed by the web parameters, such as the shear stre... | 2024 Aug 3;10(15):e35778. | Heliyon | Mazen Shrif; Samer Barakat; Zaid Al-Sadoon; Omar Mostafa; Raghad Awad; Mazen Shrif; Mazen Shrif; Samer Barakat; Zaid Al-Sadoon; Omar Mostafa; Raghad Awad | DOI:\n \n \n\n 10.1016/j.heliyon.2024.e35778 | https://pubmed.ncbi.nlm.nih.gov//39170161/ |
| 198 | Feasibility Study of Convolutional Long ShortTerm Memory Network for Pulmonary Movement Prediction in CT Images | Background:\n \n \n During X-ray imaging, pulmonary movements can cause many image artifacts. To tackle this issue, several studies, including mathematical algorithms and 2D-3D image registration methods, have been presented. Rec... | 2024 Feb 1;14(1):55-66. | J Biomed Phys Eng | Zahra Ghasemi; Payam Samadi Miandoab; Zahra Ghasemi; Zahra Ghasemi; Payam Samadi Miandoab | DOI:\n \n \n\n 10.31661/jbpe.v0i0.2105-1339 | https://pubmed.ncbi.nlm.nih.gov//38357602/ |
| 199 | Unlocking the link: predicting cardiovascular disease risk with a focus on airflow obstruction using machine learning | Background:\n \n \n Respiratory diseases and Cardiovascular Diseases (CVD) often coexist, with airflow obstruction (AO) severity closely linked to CVD incidence and mortality. As both conditions rise, early identification and int... | 2025 Feb 3;25(1):50. | BMC Med Inform Decis Mak | Xiyu Cao; Jianli Ma; Xiaoyi He; Yufei Liu; Yang Yang; Yaqi Wang; Chuantao Zhang; Xiyu Cao; Xiyu Cao; Jianli Ma; Xiaoyi He; Yufei Liu; Yang Yang; Yaqi Wang; Chuantao Zhang | DOI:\n \n \n\n 10.1186/s12911-025-02885-0 | https://pubmed.ncbi.nlm.nih.gov//39901185/ |
| 200 | An Efficient Prediction Model on the Operation Quality of Medical Equipment Based on Improved Sparrow Search Algorithm-Temporal Convolutional Network-BiLSTM | Combining medical IoT and artificial intelligence technology is an effective approach to achieve the intelligence of medical equipment. This integration can address issues such as low image quality caused by fluctuations in power quality and pote... | 2024 Aug 29;24(17):5589. | Sensors (Basel) | Zicong Lin; Zhiyong Ji; Zicong Lin; Zicong Lin; Zhiyong Ji | DOI:\n \n \n\n 10.3390/s24175589 | https://pubmed.ncbi.nlm.nih.gov//39275500/ |
| 201 | Forecasting Network Interface Flow Using a Broad Learning System Based on the Sparrow Search Algorithm | In this paper, we propose a broad learning system based on the sparrow search algorithm. Firstly, in order to avoid the complicated manual parameter tuning process and obtain the best combination of hyperparameters, the sparrow search algorithm i... | 2022 Mar 29;24(4):478. | Entropy (Basel) | Xiaoyu Li; Shaobo Li; Peng Zhou; Guanglin Chen; Xiaoyu Li; Xiaoyu Li; Shaobo Li; Peng Zhou; Guanglin Chen | DOI:\n \n \n\n 10.3390/e24040478 | https://pubmed.ncbi.nlm.nih.gov//35455141/ |
| 202 | Cortical myelin and thickness mapping provide insights into whole-brain tumor burden in diffuse midline glioma | Systemic infiltration is a hallmark of diffuse midline glioma pathogenesis, which can trigger distant disturbances in cortical structure. However, the existence and effects of these changes have been underexamined. This study aimed to investigate... | 2024 Jan 14;34(1):bhad491. | Cereb Cortex | Simin Zhang; Xibiao Yang; Qiaoyue Tan; Huaiqiang Sun; Di Chen; Yinying Chen; Hongjing Zhang; Yuan Yang; Qiyong Gong; Qiang Yue; Simin Zhang; Simin Zhang; Xibiao Yang; Qiaoyue Tan; Huaiqiang Sun; Di Chen; Yinying Chen; Hongjing Zhang; Yuan Yang; Q... | DOI:\n \n \n\n 10.1093/cercor/bhad491 | https://pubmed.ncbi.nlm.nih.gov//38112602/ |
| 203 | A Practical Approach for Targeting Structural Variants Genome-wide in Plasma Cell-free DNA | Interrogating plasma cell-free DNA (cfDNA) to detect cancer offers promise; however, no current tests scan structural variants (SVs) throughout the genome. Here, we report a simple molecular workflow to enrich a tumorigenic SV (DNA palindromes/fo... | 2024 Jan 5:rs.3.rs-3492157. | Res Sq | Hisashi Tanaka; Michael Murata; Fumie Igari; Ryan Urbanowicz; Lila Mouakkad; Sungjin Kim; Zijing Chen; Dolores Di Vizio; Edwin Posadas; Armando Giuliano; Hisashi Tanaka; Hisashi Tanaka; Michael Murata; Fumie Igari; Ryan Urbanowicz; Lila Mouakkad;... | DOI:\n \n \n\n 10.21203/rs.3.rs-3492157/v1 | https://pubmed.ncbi.nlm.nih.gov//38260372/ |
| 204 | System for Predicting Neurological Outcomes Following Cardiac Arrest Based on Clinical Predictors Using a Machine Learning Method: The Neurological Outcomes After Cardiac Arrest Method | Background:\n \n \n A multimodal approach may prove effective for predicting clinical outcomes following cardiac arrest (CA). We aimed to develop a practical predictive model that incorporates clinical factors related to CA and m... | 2025 Feb 20. | Neurocrit Care | Tae Jung Kim; Jungyo Suh; Soo-Hyun Park; Youngjoon Kim; Sang-Bae Ko; Tae Jung Kim; Tae Jung Kim; Jungyo Suh; Soo-Hyun Park; Youngjoon Kim; Sang-Bae Ko | DOI:\n \n \n\n 10.1007/s12028-025-02222-3 | https://pubmed.ncbi.nlm.nih.gov//39979708/ |
| 205 | Robust optimization of convolutional neural networks with a uniform experiment design method: a case of phonocardiogram testing in patients with heart diseases | Background:\n \n \n Heart sound measurement is crucial for analyzing and diagnosing patients with heart diseases. This study employed phonocardiogram signals as the input signal for heart disease analysis due to the accessibility... | 2021 Nov 8;22(Suppl 5):92. | BMC Bioinformatics | Wen-Hsien Ho; Tian-Hsiang Huang; Po-Yuan Yang; Jyh-Horng Chou; Jin-Yi Qu; Po-Chih Chang; Fu-I Chou; Jinn-Tsong Tsai; Wen-Hsien Ho; Wen-Hsien Ho; Tian-Hsiang Huang; Po-Yuan Yang; Jyh-Horng Chou; Jin-Yi Qu; Po-Chih Chang; Fu-I Chou; Jinn-Tsong Tsai | DOI:\n \n \n\n 10.1186/s12859-021-04032-8 | https://pubmed.ncbi.nlm.nih.gov//34749632/ |
| 206 | From fault detection to one-class severity discrimination of 3D printers with one-class support vector machine | The lack of faulty condition data reduces the feasibility of supervised learning for fault detection or fault severity discrimination in new manufacturing technologies. To deal with this issue, one-class learning arises for building binary discri... | 2021 Apr:110:357-367. | ISA Trans | Chuan Li; Diego Cabrera; Fernando Sancho; Mariela Cerrada; René-Vinicio Sánchez; Edgar Estupinan; Chuan Li; Chuan Li; Diego Cabrera; Fernando Sancho; Mariela Cerrada; René-Vinicio Sánchez; Edgar Estupinan | DOI:\n \n \n\n 10.1016/j.isatra.2020.10.036 | https://pubmed.ncbi.nlm.nih.gov//33081986/ |
| 207 | Prediction of cold region dew volume based on an ECOA-BiTCN-BiLSTM hybrid model | This paper presents a hybrid prediction model, ECOA-BiTCN-BiLSTM, for predicting dew in cold areas. The model integrates BiTCN and BiLSTM neural networks to enhance performance. An enhanced Crayfish optimization algorithm (ECOA) with four mixed s... | 2025 Feb 4;15(1):4265. | Sci Rep | Yi Zhang; Pengtao Liu; Yingying Xu; Meng Zhang; Yi Zhang; Yi Zhang; Pengtao Liu; Yingying Xu; Meng Zhang | DOI:\n \n \n\n 10.1038/s41598-024-74097-x | https://pubmed.ncbi.nlm.nih.gov//39905008/ |

In [48]:

```
## Save curated bibliometric database to CSV
df_path = '/pubmed_df.csv'
df.to_csv(df_path, index=False)
```

In [ ]:

```

```

In [ ]:

```

```

In [ ]:

```

```

In [ ]:

```

```

In [49]:

```
#######################################################
## Get corpora of documents corresponding to Pubmed searches --- total_articles2 and total_articles2_xgboost
#######################################################
```

In [50]:

```
## Second/narrower PubMed search query
query2_xgboost = '''("supervised ml" OR "supervised machine learning" OR "supervised learning" OR "binary classifier" OR "binary classification" OR "predictive model" OR "prediction model") 
AND ("hyperparameter optimization" OR "hyper-parameter optimization" OR "hyper parameter tuning" OR "hyper-parameter tuning" OR "automl" OR "automated machine learning" OR "hyperopt" OR "optuna" OR "ray-tune" OR "ray tune" OR "skopt" OR "optunity" OR "smac3")
AND ("2020/01/01"[PDAT] : "2025/03/31"[PDAT])
AND ("xgboost" OR "extreme gradient boosting")
'''
query2_xgboost
```

Out[50]:

```
'("supervised ml" OR "supervised machine learning" OR "supervised learning" OR "binary classifier" OR "binary classification" OR "predictive model" OR "prediction model") \nAND ("hyperparameter optimization" OR "hyper-parameter optimization" OR "hyper parameter tuning" OR "hyper-parameter tuning" OR "automl" OR "automated machine learning" OR "hyperopt" OR "optuna" OR "ray-tune" OR "ray tune" OR "skopt" OR "optunity" OR "smac3")\nAND ("2020/01/01"[PDAT] : "2025/03/31"[PDAT])\nAND ("xgboost" OR "extreme gradient boosting")\n'
```

In [51]:

```
## Construct base URL to PubMed (based on search query2 defined above)
pubmed_url_xgboost = "https://pubmed.ncbi.nlm.nih.gov/"
search_url_xgboost = f"{pubmed_url}?term={query2_xgboost.replace(' ', '+')}&size=100"
search_url_xgboost
```

Out[51]:

```
'https://pubmed.ncbi.nlm.nih.gov/?term=("supervised+ml"+OR+"supervised+machine+learning"+OR+"supervised+learning"+OR+"binary+classifier"+OR+"binary+classification"+OR+"predictive+model"+OR+"prediction+model")+\nAND+("hyperparameter+optimization"+OR+"hyper-parameter+optimization"+OR+"hyper+parameter+tuning"+OR+"hyper-parameter+tuning"+OR+"automl"+OR+"automated+machine+learning"+OR+"hyperopt"+OR+"optuna"+OR+"ray-tune"+OR+"ray+tune"+OR+"skopt"+OR+"optunity"+OR+"smac3")\nAND+("2020/01/01"[PDAT]+:+"2025/03/31"[PDAT])\nAND+("xgboost"+OR+"extreme+gradient+boosting")\n&size=100'
```

In [52]:

```
## Gather articles for scraping
article_urls_xgboost = get_article_urls(search_url=search_url_xgboost)
len(article_urls_xgboost)
```

Out[52]:

```
25
```

In [53]:

```
## Print articles URLs to console
article_urls_xgboost
```

Out[53]:

```
['https://pubmed.ncbi.nlm.nih.gov//37698911/',
 'https://pubmed.ncbi.nlm.nih.gov//37789305/',
 'https://pubmed.ncbi.nlm.nih.gov//36362493/',
 'https://pubmed.ncbi.nlm.nih.gov//36553069/',
 'https://pubmed.ncbi.nlm.nih.gov//31165141/',
 'https://pubmed.ncbi.nlm.nih.gov//36279027/',
 'https://pubmed.ncbi.nlm.nih.gov//35122132/',
 'https://pubmed.ncbi.nlm.nih.gov//39928609/',
 'https://pubmed.ncbi.nlm.nih.gov//35368915/',
 'https://pubmed.ncbi.nlm.nih.gov//39644793/',
 'https://pubmed.ncbi.nlm.nih.gov//37918734/',
 'https://pubmed.ncbi.nlm.nih.gov//39889299/',
 'https://pubmed.ncbi.nlm.nih.gov//38264719/',
 'https://pubmed.ncbi.nlm.nih.gov//38997304/',
 'https://pubmed.ncbi.nlm.nih.gov//39070227/',
 'https://pubmed.ncbi.nlm.nih.gov//38133687/',
 'https://pubmed.ncbi.nlm.nih.gov//34184998/',
 'https://pubmed.ncbi.nlm.nih.gov//39633817/',
 'https://pubmed.ncbi.nlm.nih.gov//38975289/',
 'https://pubmed.ncbi.nlm.nih.gov//39856375/',
 'https://pubmed.ncbi.nlm.nih.gov//38822411/',
 'https://pubmed.ncbi.nlm.nih.gov//36131178/',
 'https://pubmed.ncbi.nlm.nih.gov//40072485/',
 'https://pubmed.ncbi.nlm.nih.gov//39901185/',
 'https://pubmed.ncbi.nlm.nih.gov//39979708/']
```

In [54]:

```
## Instantiate list to hold bibliometric data from each article
articles_data_xgboost = []
```

In [55]:

```
## Loop over article URL in list and generate bibliometric data
t0 = time.time()

for url in article_urls_xgboost:
    ## Grab article info
    article_info = scrape_article(url)
    ## Append to list if available
    if article_info:
        articles_data_xgboost.append(article_info)
    ## Random sleep to respect pubMed scraping rate limits
    time.sleep(random.uniform(0, 1))  

t1 = time.time()
t1-t0
```

Out[55]:

```
37.27662944793701
```

In [56]:

```
## Convert list of Dicts object above into a pandas DataFrame
df_xgboost = pd.DataFrame(articles_data_xgboost)
df_xgboost.columns = ["title","abstract","publication_date","journal","authors","doi","url"]
df_xgboost
```

Out[56]:

|  | title | abstract | publication\_date | journal | authors | doi | url |
| --- | --- | --- | --- | --- | --- | --- | --- |
| 0 | Predicting the 5-Year Risk of Nonalcoholic Fatty Liver Disease Using Machine Learning Models: Prospective Cohort Study | Background:\n \n \n Nonalcoholic fatty liver disease (NAFLD) has emerged as a worldwide public health issue. Identifying and targeting populations at a heightened risk of developing NAFLD over a 5-year period can help reduce and ... | 2023 Sep 12:25:e46891. | J Med Internet Res | Guoqing Huang; Qiankai Jin; Yushan Mao; Guoqing Huang; Guoqing Huang; Qiankai Jin; Yushan Mao | DOI:\n \n \n\n 10.2196/46891 | https://pubmed.ncbi.nlm.nih.gov//37698911/ |
| 1 | Machine learning-based prediction model of acute kidney injury in patients with acute respiratory distress syndrome | Background:\n \n \n Acute kidney injury (AKI) can make cases of acute respiratory distress syndrome (ARDS) more complex, and the combination of the two can significantly worsen the prognosis. Our objective is to utilize machine l... | 2023 Oct 3;23(1):370. | BMC Pulm Med | Shuxing Wei; Yongsheng Zhang; Hongmeng Dong; Ying Chen; Xiya Wang; Xiaomei Zhu; Guang Zhang; Shubin Guo; Shuxing Wei; Shuxing Wei; Yongsheng Zhang; Hongmeng Dong; Ying Chen; Xiya Wang; Xiaomei Zhu; Guang Zhang; Shubin Guo | DOI:\n \n \n\n 10.1186/s12890-023-02663-6 | https://pubmed.ncbi.nlm.nih.gov//37789305/ |
| 2 | Explainable Preoperative Automated Machine Learning Prediction Model for Cardiac Surgery-Associated Acute Kidney Injury | Background:\n \n \n We aimed to develop and validate an automated machine learning (autoML) prediction model for cardiac surgery-associated acute kidney injury (CSA-AKI).\n \n\n\n Methods:\n \n \n Usi... | 2022 Oct 24;11(21):6264. | J Clin Med | Charat Thongprayoon; Pattharawin Pattharanitima; Andrea G Kattah; Michael A Mao; Mira T Keddis; John J Dillon; Wisit Kaewput; Supawit Tangpanithandee; Pajaree Krisanapan; Fawad Qureshi; Wisit Cheungpasitporn; Charat Thongprayoon; Charat Thongpray... | DOI:\n \n \n\n 10.3390/jcm11216264 | https://pubmed.ncbi.nlm.nih.gov//36362493/ |
| 3 | Blood Glucose Prediction Method Based on Particle Swarm Optimization and Model Fusion | Blood glucose stability in diabetic patients determines the degree of health, and changes in blood glucose levels are related to the outcome of diabetic patients. Therefore, accurate monitoring of blood glucose has a crucial role in controlling d... | 2022 Dec 6;12(12):3062. | Diagnostics (Basel) | He Xu; Shanjun Bao; Xiaoyu Zhang; Shangdong Liu; Wei Jing; Yimu Ji; He Xu; He Xu; Shanjun Bao; Xiaoyu Zhang; Shangdong Liu; Wei Jing; Yimu Ji | DOI:\n \n \n\n 10.3390/diagnostics12123062 | https://pubmed.ncbi.nlm.nih.gov//36553069/ |
| 4 | Scaling tree-based automated machine learning to biomedical big data with a feature set selector | Motivation:\n \n \n Automated machine learning (AutoML) systems are helpful data science assistants designed to scan data for novel features, select appropriate supervised learning models and optimize their parameters. For this p... | 2020 Jan 1;36(1):250-256. | Bioinformatics | Trang T Le; Weixuan Fu; Jason H Moore; Trang T Le; Trang T Le; Weixuan Fu; Jason H Moore | DOI:\n \n \n\n 10.1093/bioinformatics/btz470 | https://pubmed.ncbi.nlm.nih.gov//31165141/ |
| 5 | Automated Multimodal Machine Learning for Esophageal Variceal Bleeding Prediction Based on Endoscopy and Structured Data | Esophageal variceal (EV) bleeding is a severe medical emergency related to cirrhosis. Early identification of cirrhotic patients with at a high risk of EV bleeding is key to improving outcomes and optimizing medical resources. This study aimed to... | 2023 Feb;36(1):326-338. | J Digit Imaging | Yu Wang; Yu Hong; Yue Wang; Xin Zhou; Xin Gao; Chenyan Yu; Jiaxi Lin; Lu Liu; Jingwen Gao; Minyue Yin; Guoting Xu; Xiaolin Liu; Jinzhou Zhu; Yu Wang; Yu Wang; Yu Hong; Yue Wang; Xin Zhou; Xin Gao; Chenyan Yu; Jiaxi Lin; Lu Liu; Jingwen Gao; Minyu... | DOI:\n \n \n\n 10.1007/s10278-022-00724-6 | https://pubmed.ncbi.nlm.nih.gov//36279027/ |
| 6 | Evaluating an automated machine learning model that predicts visual acuity outcomes in patients with neovascular age-related macular degeneration | Purpose:\n \n \n Neovascular age-related macular degeneration (nAMD) is a major global cause of blindness. Whilst anti-vascular endothelial growth factor (anti-VEGF) treatment is effective, response varies considerably between in... | 2022 Aug;260(8):2461-2473. | Graefes Arch Clin Exp Ophthalmol | Abdallah Abbas; Ciara O'Byrne; Dun Jack Fu; Gabriella Moraes; Konstantinos Balaskas; Robbert Struyven; Sara Beqiri; Siegfried K Wagner; Edward Korot; Pearse A Keane; Abdallah Abbas; Abdallah Abbas; Ciara O'Byrne; Dun Jack Fu; Gabriella Moraes; Ko... | DOI:\n \n \n\n 10.1007/s00417-021-05544-y | https://pubmed.ncbi.nlm.nih.gov//35122132/ |
| 7 | Enhancing machine learning performance in cardiac surgery ICU: Hyperparameter optimization with metaheuristic algorithm | The healthcare industry is generating a massive volume of data, promising a potential goldmine of information that can be extracted through machine learning (ML) techniques. The Intensive Care Unit (ICU) stands out as a focal point within hospita... | 2025 Feb 10;20(2):e0311250. | PLoS One | Ali Bahrami; Morteza Rakhshaninejad; Rouzbeh Ghousi; Alireza Atashi; Ali Bahrami; Ali Bahrami; Morteza Rakhshaninejad; Rouzbeh Ghousi; Alireza Atashi | DOI:\n \n \n\n 10.1371/journal.pone.0311250 | https://pubmed.ncbi.nlm.nih.gov//39928609/ |
| 8 | COVID-19 Risk Prediction for Diabetic Patients Using Fuzzy Inference System and Machine Learning Approaches | Individuals with pre-existing diabetes seem to be vulnerable to the COVID-19 due to changes in blood sugar levels and diabetes complications. As observed globally, around 20-50% of individuals affected by coronavirus had diabetes. However, there ... | 2022 Apr 1:2022:4096950. | J Healthc Eng | Alok Aggarwal; Madam Chakradar; Manpreet Singh Bhatia; Manoj Kumar; Thompson Stephan; Sachin Kumar Gupta; S H Alsamhi; Hatem Al-Dois; Alok Aggarwal; Alok Aggarwal; Madam Chakradar; Manpreet Singh Bhatia; Manoj Kumar; Thompson Stephan; Sachin Kuma... | DOI:\n \n \n\n 10.1155/2022/4096950 | https://pubmed.ncbi.nlm.nih.gov//35368915/ |
| 9 | Construction and evaluation of prediction model for postoperative re-fractures in elderly patients with hip fractures | Objective:\n \n \n The aim of study was to construct a postoperative re-fracture prediction model for elderly hip fracture patients using an automated machine learning algorithm to provide a basis for early identification of pati... | 2025 Mar:195:105738. | Int J Med Inform | Jingjing Wu; Qingqing Zeng; Sijie Gui; Zhuolan Li; Wanyu Miao; Mi Zeng; Manyi Wang; Li Hu; Guqing Zeng; Jingjing Wu; Jingjing Wu; Qingqing Zeng; Sijie Gui; Zhuolan Li; Wanyu Miao; Mi Zeng; Manyi Wang; Li Hu; Guqing Zeng | DOI:\n \n \n\n 10.1016/j.ijmedinf.2024.105738 | https://pubmed.ncbi.nlm.nih.gov//39644793/ |
| 10 | Application of interpretable machine learning models to improve the prediction performance of ionic liquids toxicity | With the wide application prospect of ionic liquids (ILs) as solvent in the future industry, in order to promote green and sustainable chemical engineering, the toxicity problem of common concern has been systematically modeled. Machine learning ... | 2024 Jan 15:908:168168. | Sci Total Environ | Dingchao Fan; Ke Xue; Runqi Zhang; Wenguang Zhu; Hongru Zhang; Jianguang Qi; Zhaoyou Zhu; Yinglong Wang; Peizhe Cui; Dingchao Fan; Dingchao Fan; Ke Xue; Runqi Zhang; Wenguang Zhu; Hongru Zhang; Jianguang Qi; Zhaoyou Zhu; Yinglong Wang; Peizhe Cui | DOI:\n \n \n\n 10.1016/j.scitotenv.2023.168168 | https://pubmed.ncbi.nlm.nih.gov//37918734/ |
| 11 | Machine Learning-Based Risk Factor Analysis and Prediction Model Construction for the Occurrence of Chronic Heart Failure: Health Ecologic Study | Background:\n \n \n Chronic heart failure (CHF) is a serious threat to human health, with high morbidity and mortality rates, imposing a heavy burden on the health care system and society. With the abundance of medical data and t... | 2025 Jan 31:13:e64972. | JMIR Med Inform | Qian Xu; Xue Cai; Ruicong Yu; Yueyue Zheng; Guanjie Chen; Hui Sun; Tianyun Gao; Cuirong Xu; Jing Sun; Qian Xu; Qian Xu; Xue Cai; Ruicong Yu; Yueyue Zheng; Guanjie Chen; Hui Sun; Tianyun Gao; Cuirong Xu; Jing Sun | DOI:\n \n \n\n 10.2196/64972 | https://pubmed.ncbi.nlm.nih.gov//39889299/ |
| 12 | AlphaML: A clear, legible, explainable, transparent, and elucidative binary classification platform for tabular data | Leveraging the potential of machine learning and recognizing the broad applications of binary classification, it becomes essential to develop platforms that are not only powerful but also transparent, interpretable, and user friendly. We introduc... | 2023 Dec 13;5(1):100897. | Patterns (N Y) | Ahmad Nasimian; Saleena Younus; Özge Tatli; Emma U Hammarlund; Kenneth J Pienta; Lars Rönnstrand; Julhash U Kazi; Ahmad Nasimian; Ahmad Nasimian; Saleena Younus; Özge Tatli; Emma U Hammarlund; Kenneth J Pienta; Lars Rönnstrand; Julhash U Kazi | DOI:\n \n \n\n 10.1016/j.patter.2023.100897 | https://pubmed.ncbi.nlm.nih.gov//38264719/ |
| 13 | Long-term prediction modeling of shallow rockburst with small dataset based on machine learning | Rockburst present substantial hazards in both deep underground construction and shallow depths, underscoring the critical need for accurate prediction methods. This study addressed this need by collecting and analyzing 69 real datasets of rockbur... | 2024 Jul 12;14(1):16131. | Sci Rep | Guozhu Rao; Yunzhang Rao; Jiazheng Wan; Qiang Huang; Yangjun Xie; Qiande Lai; Zhihua Yang; Run Xiang; Laiye Zhang; Guozhu Rao; Guozhu Rao; Yunzhang Rao; Jiazheng Wan; Qiang Huang; Yangjun Xie; Qiande Lai; Zhihua Yang; Run Xiang; Laiye Zhang | DOI:\n \n \n\n 10.1038/s41598-024-64107-3 | https://pubmed.ncbi.nlm.nih.gov//38997304/ |
| 14 | Development and Validation of an ICU-Venous Thromboembolism Prediction Model Using Machine Learning Approaches: A Multicenter Study | Purpose:\n \n \n The purpose of this study was to establish and validate machine learning-based models for predicting the risk of venous thromboembolism (VTE) in intensive care unit (ICU) patients.\n \n\n\n Patients a... | 2024 Jul 24:17:3279-3292. | Int J Gen Med | Jie Jin; Jie Lu; Xinyang Su; Yinhuan Xiong; Shasha Ma; Yang Kong; Hongmei Xu; Jie Jin; Jie Jin; Jie Lu; Xinyang Su; Yinhuan Xiong; Shasha Ma; Yang Kong; Hongmei Xu | DOI:\n \n \n\n 10.2147/IJGM.S467374 | https://pubmed.ncbi.nlm.nih.gov//39070227/ |
| 15 | Prediction of therapy response of breast cancer patients with machine learning based on clinical data and imaging data derived from breast [18F]FDG-PET/MRI | Purpose:\n \n \n To evaluate if a machine learning prediction model based on clinical and easily assessable imaging features derived from baseline breast [18F]FDG-PET/MRI staging can predict pathologic complete response (pCR) in ... | 2024 Apr;51(5):1451-1461. | Eur J Nucl Med Mol Imaging | Kai Jannusch; Frederic Dietzel; Nils Martin Bruckmann; Janna Morawitz; Matthias Boschheidgen; Peter Minko; Ann-Kathrin Bittner; Svjetlana Mohrmann; Harald H Quick; Ken Herrmann; Lale Umutlu; Gerald Antoch; Christian Rubbert; Julian Kirchner; Juli... | DOI:\n \n \n\n 10.1007/s00259-023-06513-9 | https://pubmed.ncbi.nlm.nih.gov//38133687/ |
| 16 | Discovery of Depression-Associated Factors From a Nationwide Population-Based Survey: Epidemiological Study Using Machine Learning and Network Analysis | Background:\n \n \n In epidemiological studies, finding the best subset of factors is challenging when the number of explanatory variables is large.\n \n\n\n Objective:\n \n \n Our study had two aims.... | 2021 Jun 24;23(6):e27344. | J Med Internet Res | Sang Min Nam; Thomas A Peterson; Kyoung Yul Seo; Hyun Wook Han; Jee In Kang; Sang Min Nam; Sang Min Nam; Thomas A Peterson; Kyoung Yul Seo; Hyun Wook Han; Jee In Kang | DOI:\n \n \n\n 10.2196/27344 | https://pubmed.ncbi.nlm.nih.gov//34184998/ |
| 17 | Exploring machine learning algorithms to predict acute respiratory tract infection and identify its determinants among children under five in Sub-Saharan Africa | Background:\n \n \n The primary cause of death for children under the age of five is acute respiratory infections (ARI). Early predicting acute respiratory tract infections (ARI) and identifying their predictors using supervised ... | 2024 Nov 20:12:1388820. | Front Pediatr | Tirualem Zeleke Yehuala; Bezawit Melak Fente; Sisay Maru Wubante; Nebiyu Mekonnen Derseh; Tirualem Zeleke Yehuala; Tirualem Zeleke Yehuala; Bezawit Melak Fente; Sisay Maru Wubante; Nebiyu Mekonnen Derseh | DOI:\n \n \n\n 10.3389/fped.2024.1388820 | https://pubmed.ncbi.nlm.nih.gov//39633817/ |
| 18 | Deep neural network-based prediction of tsunami wave attenuation by mangrove forests | The goal of this research is to develop a model employing deep neural networks (DNNs) to predict the effectiveness of mangrove forests in attenuating the impact of tsunami waves. The dataset for the DNN model is obtained by simulating tsunami wav... | 2024 Jun 11:13:102791. | MethodsX | Didit Adytia; Dede Tarwidi; Deni Saepudin; Semeidi Husrin; Abdul Rahman Mohd Kasim; Mohd Fakhizan Romlie; Dafrizal Samsudin; Didit Adytia; Didit Adytia; Dede Tarwidi; Deni Saepudin; Semeidi Husrin; Abdul Rahman Mohd Kasim; Mohd Fakhizan Romlie; D... | DOI:\n \n \n\n 10.1016/j.mex.2024.102791 | https://pubmed.ncbi.nlm.nih.gov//38975289/ |
| 19 | Prediction of microvascular obstruction from angio-based microvascular resistance and available clinical data in percutaneous coronary intervention: an explainable machine learning model | Angio-based microvascular resistance (AMR) as a potential alternative to the index of microcirculatory resistance (IMR) and its relationship with microvascular obstruction (MVO) and other cardiac magnetic resonance (CMR) parameters still lacks co... | 2025 Jan 24;15(1):3045. | Sci Rep | Zhe Zhang; Yang Dai; Peng Xue; Xue Bao; Xinbo Bai; Shiyang Qiao; Yuan Gao; Xuemei Guo; Yanan Xue; Qing Dai; Biao Xu; Lina Kang; Zhe Zhang; Zhe Zhang; Yang Dai; Peng Xue; Xue Bao; Xinbo Bai; Shiyang Qiao; Yuan Gao; Xuemei Guo; Yanan Xue; Qing Dai;... | DOI:\n \n \n\n 10.1038/s41598-025-87828-5 | https://pubmed.ncbi.nlm.nih.gov//39856375/ |
| 20 | Optimizing PGRs for in vitro shoot proliferation of pomegranate with bayesian-tuned ensemble stacking regression and NSGA-II: a comparative evaluation of machine learning models | Background:\n \n \n The process of optimizing in vitro shoot proliferation is a complicated task, as it is influenced by interactions of many factors as well as genotype. This study investigated the role of various concentrations... | 2024 May 31;20(1):82. | Plant Methods | Saeedeh Zarbakhsh; Ali Reza Shahsavar; Mohammad Soltani; Saeedeh Zarbakhsh; Saeedeh Zarbakhsh; Ali Reza Shahsavar; Mohammad Soltani | DOI:\n \n \n\n 10.1186/s13007-024-01211-5 | https://pubmed.ncbi.nlm.nih.gov//38822411/ |
| 21 | A data-driven interpretable ensemble framework based on tree models for forecasting the occurrence of COVID-19 in the USA | This prevalence of coronavirus disease 2019 (COVID-19) has become one of the most serious public health crises. Tree-based machine learning methods, with the advantages of high efficiency, and strong interpretability, have been widely used in pre... | 2023 Jan;30(5):13648-13659. | Environ Sci Pollut Res Int | Hu-Li Zheng; Shu-Yi An; Bao-Jun Qiao; Peng Guan; De-Sheng Huang; Wei Wu; Hu-Li Zheng; Hu-Li Zheng; Shu-Yi An; Bao-Jun Qiao; Peng Guan; De-Sheng Huang; Wei Wu | DOI:\n \n \n\n 10.1007/s11356-022-23132-3 | https://pubmed.ncbi.nlm.nih.gov//36131178/ |
| 22 | Two-Year Hypertension Incidence Risk Prediction in Populations in the Desert Regions of Northwest China: Prospective Cohort Study | Background:\n \n \n Hypertension is a major global health issue and a significant modifiable risk factor for cardiovascular diseases, contributing to a substantial socioeconomic burden due to its high prevalence. In China, partic... | 2025 Mar 12:27:e68442. | J Med Internet Res | Yinlin Cheng; Kuiying Gu; Weidong Ji; Zhensheng Hu; Yining Yang; Yi Zhou; Yinlin Cheng; Yinlin Cheng; Kuiying Gu; Weidong Ji; Zhensheng Hu; Yining Yang; Yi Zhou | DOI:\n \n \n\n 10.2196/68442 | https://pubmed.ncbi.nlm.nih.gov//40072485/ |
| 23 | Unlocking the link: predicting cardiovascular disease risk with a focus on airflow obstruction using machine learning | Background:\n \n \n Respiratory diseases and Cardiovascular Diseases (CVD) often coexist, with airflow obstruction (AO) severity closely linked to CVD incidence and mortality. As both conditions rise, early identification and int... | 2025 Feb 3;25(1):50. | BMC Med Inform Decis Mak | Xiyu Cao; Jianli Ma; Xiaoyi He; Yufei Liu; Yang Yang; Yaqi Wang; Chuantao Zhang; Xiyu Cao; Xiyu Cao; Jianli Ma; Xiaoyi He; Yufei Liu; Yang Yang; Yaqi Wang; Chuantao Zhang | DOI:\n \n \n\n 10.1186/s12911-025-02885-0 | https://pubmed.ncbi.nlm.nih.gov//39901185/ |
| 24 | System for Predicting Neurological Outcomes Following Cardiac Arrest Based on Clinical Predictors Using a Machine Learning Method: The Neurological Outcomes After Cardiac Arrest Method | Background:\n \n \n A multimodal approach may prove effective for predicting clinical outcomes following cardiac arrest (CA). We aimed to develop a practical predictive model that incorporates clinical factors related to CA and m... | 2025 Feb 20. | Neurocrit Care | Tae Jung Kim; Jungyo Suh; Soo-Hyun Park; Youngjoon Kim; Sang-Bae Ko; Tae Jung Kim; Tae Jung Kim; Jungyo Suh; Soo-Hyun Park; Youngjoon Kim; Sang-Bae Ko | DOI:\n \n \n\n 10.1007/s12028-025-02222-3 | https://pubmed.ncbi.nlm.nih.gov//39979708/ |

In [57]:

```
## Save curated bibliometric database to CSV
df_path_xgboost = '/pubmed_df_xgboost.csv'
df_xgboost.to_csv(df_path_xgboost, index=False)
```

In [ ]:

```

```

In [ ]:

```

```

In [ ]:

```

```

In [ ]:

```

```

In [58]:

```
#############################################
## Execution date
#############################################
from datetime import datetime
datetime.today().strftime('%Y-%m-%d')
```

Out[58]:

```
'2025-04-07'
```

In [59]:

```
#############################################
## Python version
#############################################
import sys
sys.version
```

Out[59]:

```
'3.13.1 | packaged by conda-forge | (main, Jan 13 2025, 09:37:28) [MSC v.1942 64 bit (AMD64)]'
```

In [60]:

```
#############################################
## Jupyter version
#############################################
import jupyter
```

In [61]:

```
#############################################
## Modules/dependencies
#############################################
import sys

module_list = set([m.split(".")[0] for m in sys.modules])
module_list
```

Out[61]:

```
{'IPython',
 '__future__',
 '__main__',
 '_abc',
 '_ast',
 '_asyncio',
 '_bisect',
 '_blake2',
 '_bz2',
 '_codecs',
 '_collections',
 '_collections_abc',
 '_colorize',
 '_compat_pickle',
 '_compression',
 '_contextvars',
 '_csv',
 '_ctypes',
 '_cython_3_0_11',
 '_datetime',
 '_decimal',
 '_distutils_hack',
 '_frozen_importlib',
 '_frozen_importlib_external',
 '_functools',
 '_hashlib',
 '_heapq',
 '_imp',
 '_io',
 '_json',
 '_locale',
 '_lsprof',
 '_lzma',
 '_markupbase',
 '_multibytecodec',
 '_opcode',
 '_opcode_metadata',
 '_operator',
 '_overlapped',
 '_pickle',
 '_pydev_bundle',
 '_pydev_runfiles',
 '_pydevd_bundle',
 '_pydevd_frame_eval',
 '_pydevd_sys_monitoring',
 '_pydevd_sys_monitoring_cython',
 '_pyrepl',
 '_queue',
 '_random',
 '_signal',
 '_sitebuiltins',
 '_socket',
 '_sqlite3',
 '_sre',
 '_ssl',
 '_stat',
 '_string',
 '_strptime',
 '_struct',
 '_sysconfig',
 '_thread',
 '_tokenize',
 '_typing',
 '_uuid',
 '_warnings',
 '_weakref',
 '_weakrefset',
 '_winapi',
 '_wmi',
 '_zoneinfo',
 'abc',
 'argparse',
 'array',
 'ast',
 'asttokens',
 'asyncio',
 'atexit',
 'base64',
 'bdb',
 'binascii',
 'bisect',
 'bs4',
 'builtins',
 'bz2',
 'cProfile',
 'calendar',
 'certifi',
 'charset_normalizer',
 'cmath',
 'cmd',
 'code',
 'codecs',
 'codeop',
 'collections',
 'colorama',
 'colorsys',
 'comm',
 'concurrent',
 'contextlib',
 'contextvars',
 'copy',
 'copyreg',
 'csv',
 'ctypes',
 'cython_runtime',
 'dataclasses',
 'datetime',
 'dateutil',
 'debugpy',
 'decimal',
 'decorator',
 'difflib',
 'dis',
 'email',
 'encodings',
 'enum',
 'errno',
 'executing',
 'faulthandler',
 'filecmp',
 'fnmatch',
 'fractions',
 'functools',
 'gc',
 'genericpath',
 'getopt',
 'getpass',
 'gettext',
 'glob',
 'gzip',
 'hashlib',
 'heapq',
 'hmac',
 'html',
 'http',
 'idna',
 'importlib',
 'inspect',
 'io',
 'ipaddress',
 'ipykernel',
 'itertools',
 'jedi',
 'json',
 'jupyter',
 'jupyter_client',
 'jupyter_core',
 'keyword',
 'linecache',
 'locale',
 'logging',
 'lzma',
 'marshal',
 'math',
 'mimetypes',
 'mmap',
 'msvcrt',
 'netrc',
 'nt',
 'ntpath',
 'nturl2path',
 'numbers',
 'numpy',
 'opcode',
 'operator',
 'os',
 'packaging',
 'pandas',
 'parso',
 'pathlib',
 'pdb',
 'pickle',
 'pkgutil',
 'platform',
 'platformdirs',
 'posixpath',
 'pprint',
 'profile',
 'prompt_toolkit',
 'pstats',
 'psutil',
 'pure_eval',
 'pydev_ipython',
 'pydevconsole',
 'pydevd',
 'pydevd_file_utils',
 'pydevd_plugins',
 'pydevd_tracing',
 'pydoc',
 'pydoc_data',
 'pyexpat',
 'pygments',
 'pytz',
 'pywin32_bootstrap',
 'pywin32_system32',
 'queue',
 'quopri',
 'random',
 're',
 'reprlib',
 'requests',
 'rlcompleter',
 'runpy',
 'secrets',
 'select',
 'selectors',
 'shlex',
 'shutil',
 'signal',
 'site',
 'six',
 'socket',
 'socketserver',
 'soupsieve',
 'sqlite3',
 'ssl',
 'stack_data',
 'stat',
 'string',
 'stringprep',
 'struct',
 'subprocess',
 'sys',
 'sysconfig',
 'tarfile',
 'tempfile',
 'textwrap',
 'threading',
 'time',
 'timeit',
 'token',
 'tokenize',
 'tornado',
 'traceback',
 'traitlets',
 'types',
 'typing',
 'unicodedata',
 'urllib',
 'urllib3',
 'uuid',
 'warnings',
 'wcwidth',
 'weakref',
 'winreg',
 'xml',
 'xmlrpc',
 'zipfile',
 'zipimport',
 'zlib',
 'zmq',
 'zoneinfo'}
```

In [ ]:

```

```

In [ ]:

```

```

In [ ]:

```

```
